# Supplementary figures and images for: Inhibition of HER2 signaling and breast cancer cell growth with a novel antibody targeting HER2 ECD III/IV
Source: PLoS One. 2026 Jan 15;21(1):e0338127. doi: 10.1371/journal.pone.0338127 (PMC12806845; doi:10.1371/journal.pone.0338127)

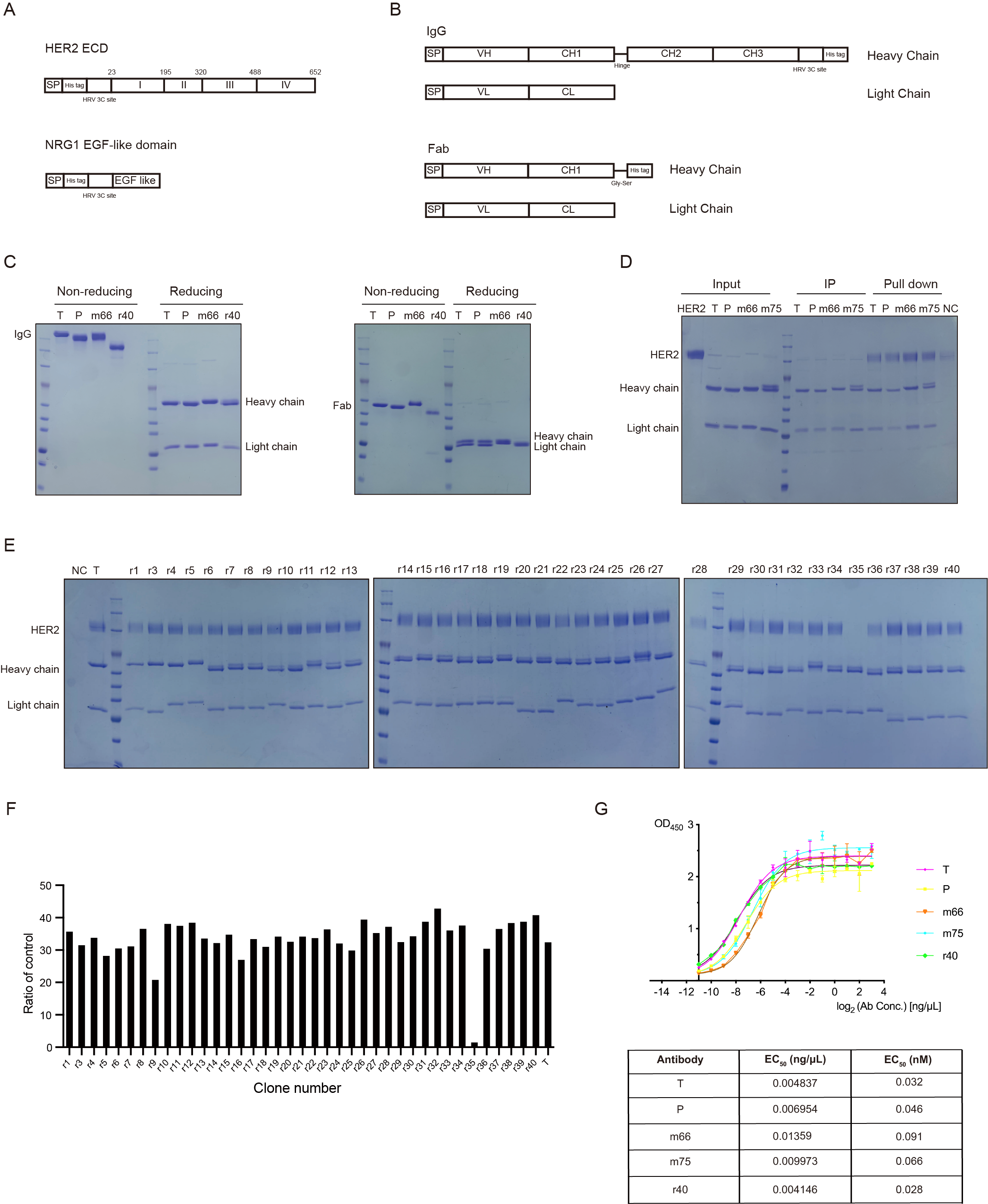

Supplement: S1 Fig — A. Schematic representation of the recombinant HER2 ECD and NGR1 EGF-like domain constructs. SP, signal peptide. B. Construct designs for the IgGs and Fabs used in this study. SP, signal peptide. C. SDS-PAGE analysis under both non-reducing and reducing conditions confirming the purity and integrity of the purified anti-HER2 IgGs and Fabs. D. Pull-down assay assessing the binding specificity of mouse antibodies. NC, negative control. E. Pull-down assay evaluating the reactivity of rabbit antibodies. F. ELISA for characterizing the affinity and specificity of rabbit monoclonal antibodies. G. Half-maximal effective concentration (EC50) values of the indicated antibodies, as determined by ELISA. (PNG) [file pone.0338127.s001.png]

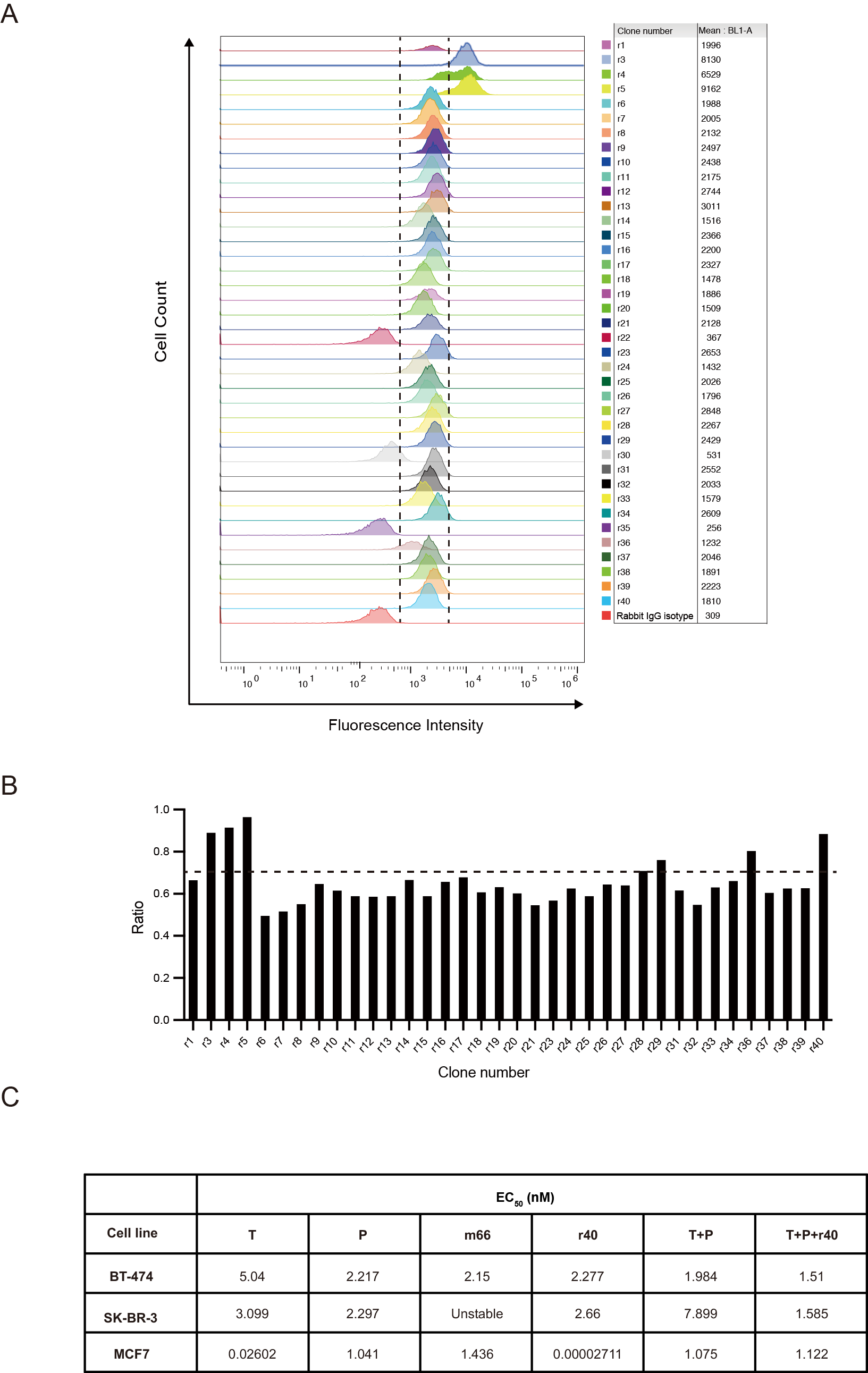

Supplement: S2 Fig — A. Binding of rabbit anti-HER2 antibodies (10 μg/mL) to Expi293 cells, compared to the rabbit isotype control IgG. B. Competitive binding profile of 36 rabbit antibodies on Expi293 cells, assessed by flow cytometry. The x-axis identifies each antibody clone; the y-axis shows the ratio of MFI in the presence versus absence of trastuzumab and pertuzumab (each at 10 μg/mL). C. EC50 values of individual antibodies, the trastuzumab-pertuzumab combination, and the triple combination, determined by CCK-8 assay in three breast cancer cell lines. (PNG) [file pone.0338127.s002.png]

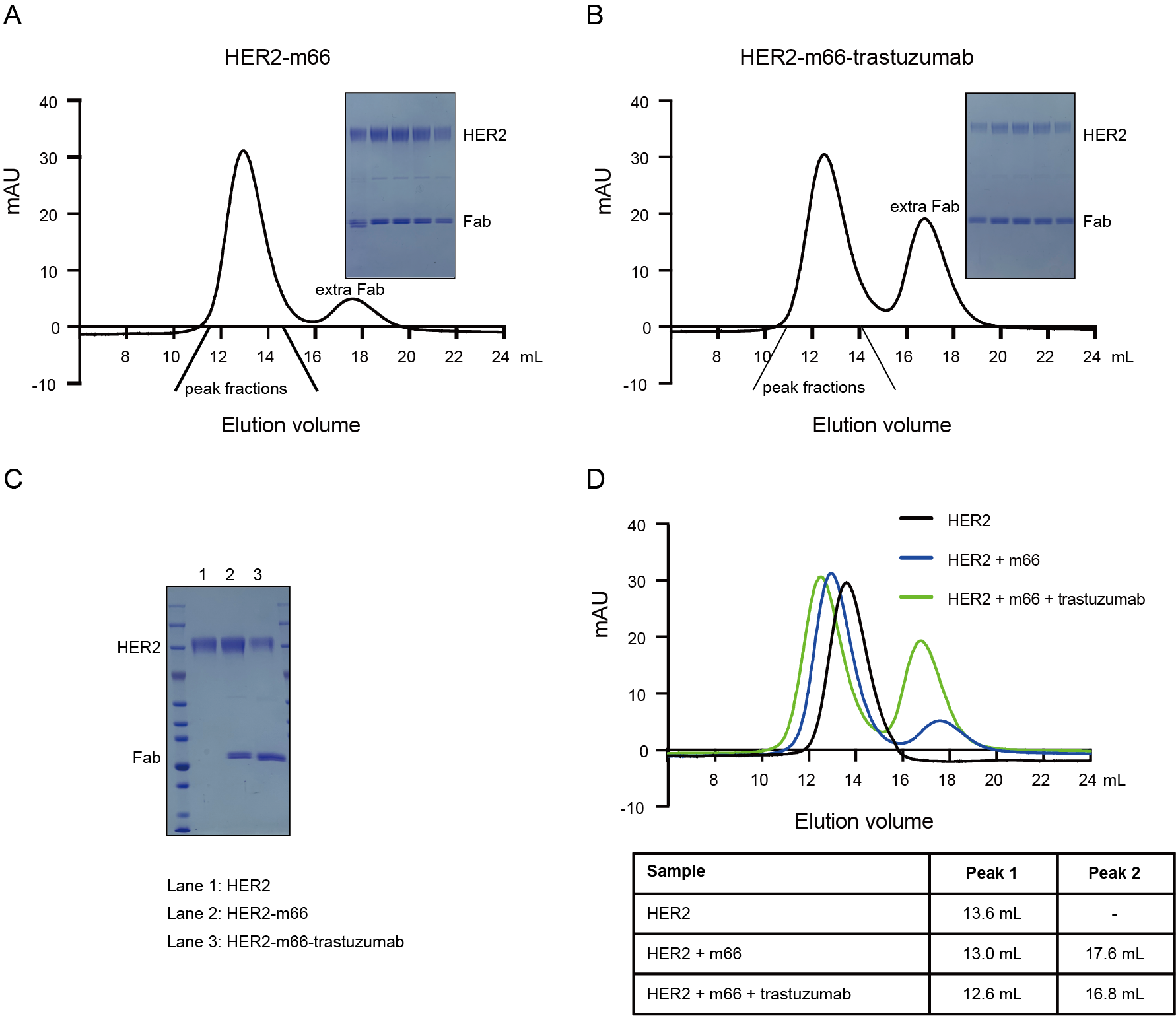

Supplement: S3 Fig — A. SEC profile of the HER2-m66 binary complex, showing elution ahead of unbound m66 Fab. B. SEC trace after incubating the purified HER2-m66 binary complex with trastuzumab Fab, confirming the formation of a ternary complex. C. SDS-PAGE analysis under reducing conditions validates the composition and purity of HER2, HER2-m66 binary complex and HER2-m66-trastuzumab ternary complex. D. Schematic representation of the complex assembly process: HER2 ECD first binds m66 Fab to form a binary complex, which further incorporates trastuzumab Fab to generate a ternary complex. (PNG) [file pone.0338127.s003.png]

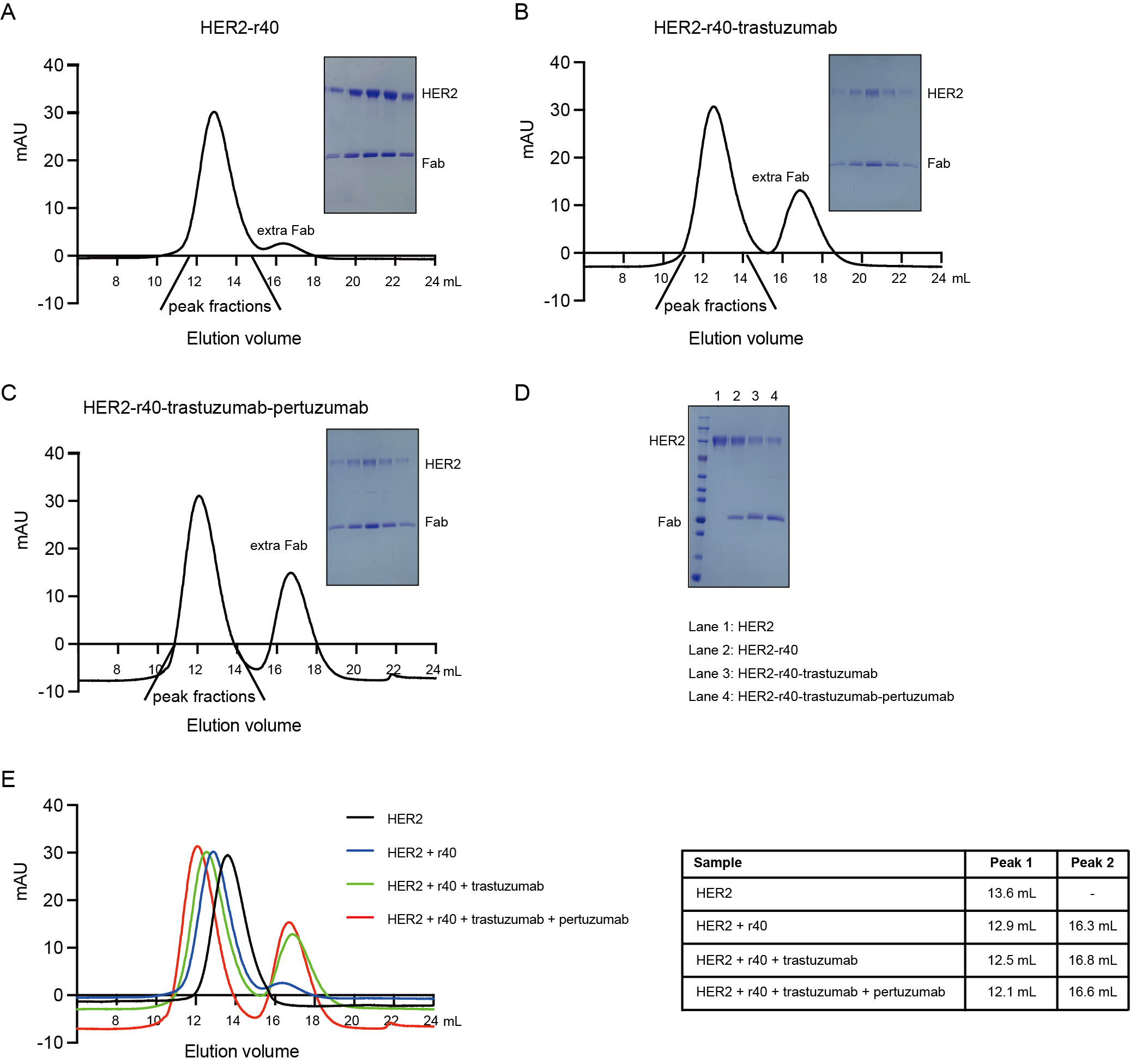

Supplement: S4 Fig — A. SEC profile showing the formation of the HER2-r40 Fab binary complex. B. SEC trace after incubation of the purified HER2-r40 complex with trastuzumab Fab. C. SEC profile of the final tetrameric complex following further incubation with pertuzumab Fab. D. SDS-PAGE analysis under reducing conditions of the purified HER2 ECD, HER2-r40, HER2-r40-trastuzumab and HER2-r40-trastuzumab-pertuzumab complexes. E. Schematic illustrating the sequential assembly: HER2 binds sequentially to r40 Fab, trastuzumab Fab, and pertuzumab Fab to form the tetrameric complex. (PNG) [file pone.0338127.s004.png]

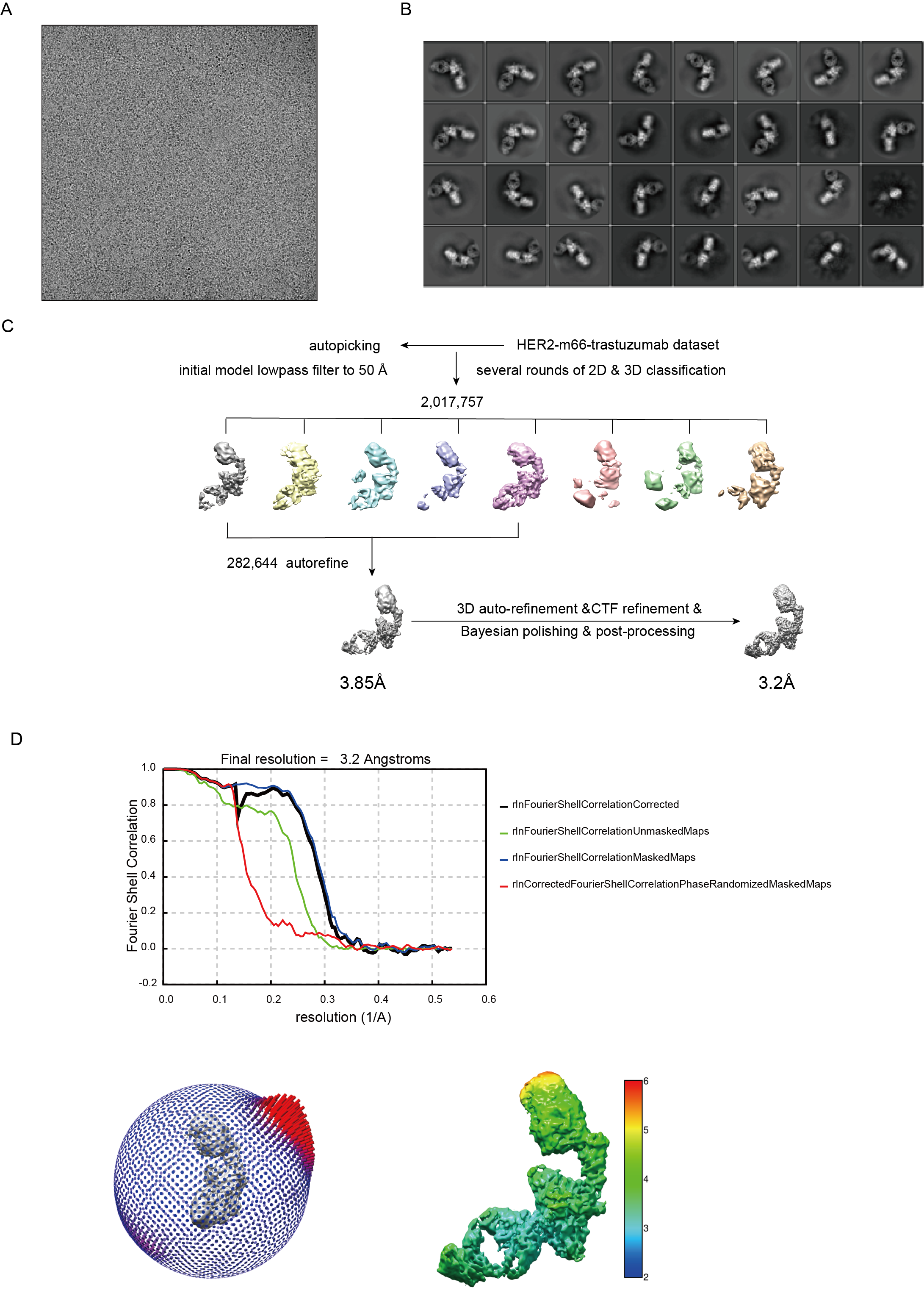

Supplement: S5 Fig — (A) Representative cryo-EM raw micrograph. (B) Results of 2D classification. (C) Schematic workflow of cryo-EM image processing and 3D reconstruction steps. (D) The upper panel displays the Fourier shell correlation (FSC) curve, while the lower left and right panels illustrate the distribution of cryo-EM map orientations and estimates of local resolution, respectively. (PNG) [file pone.0338127.s005.png]

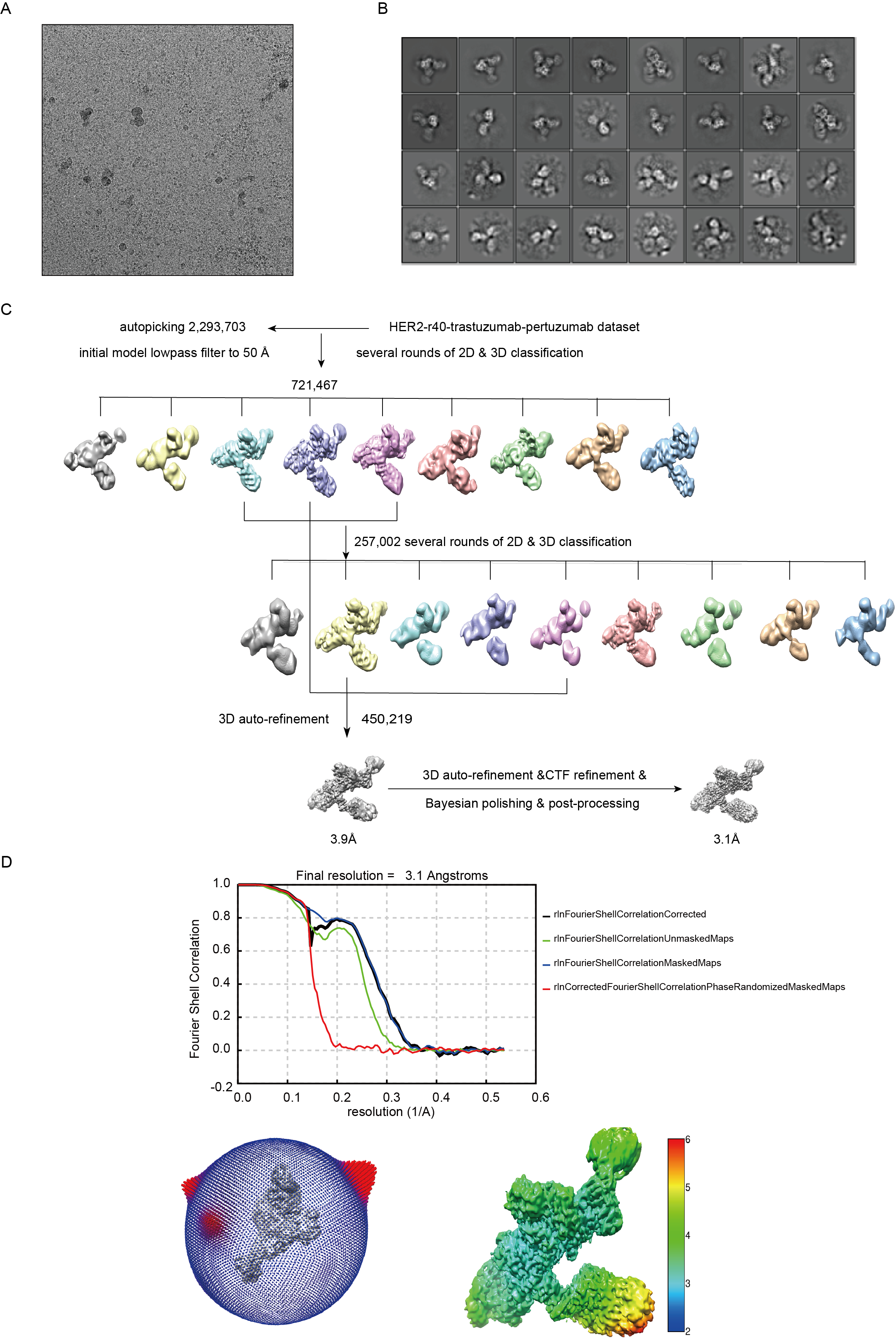

Supplement: S6 Fig — (A) Representative cryo-EM raw micrograph. (B) Results of 2D classification. (C) Schematic diagram for cryo-EM image processing and 3D reconstruction workflow. (D) The upper panel shows the FSC curve, while the lower left and right panels illustrate the distribution of cryo-EM map orientations and estimates of local resolution, respectively. (PNG) [file pone.0338127.s006.png]

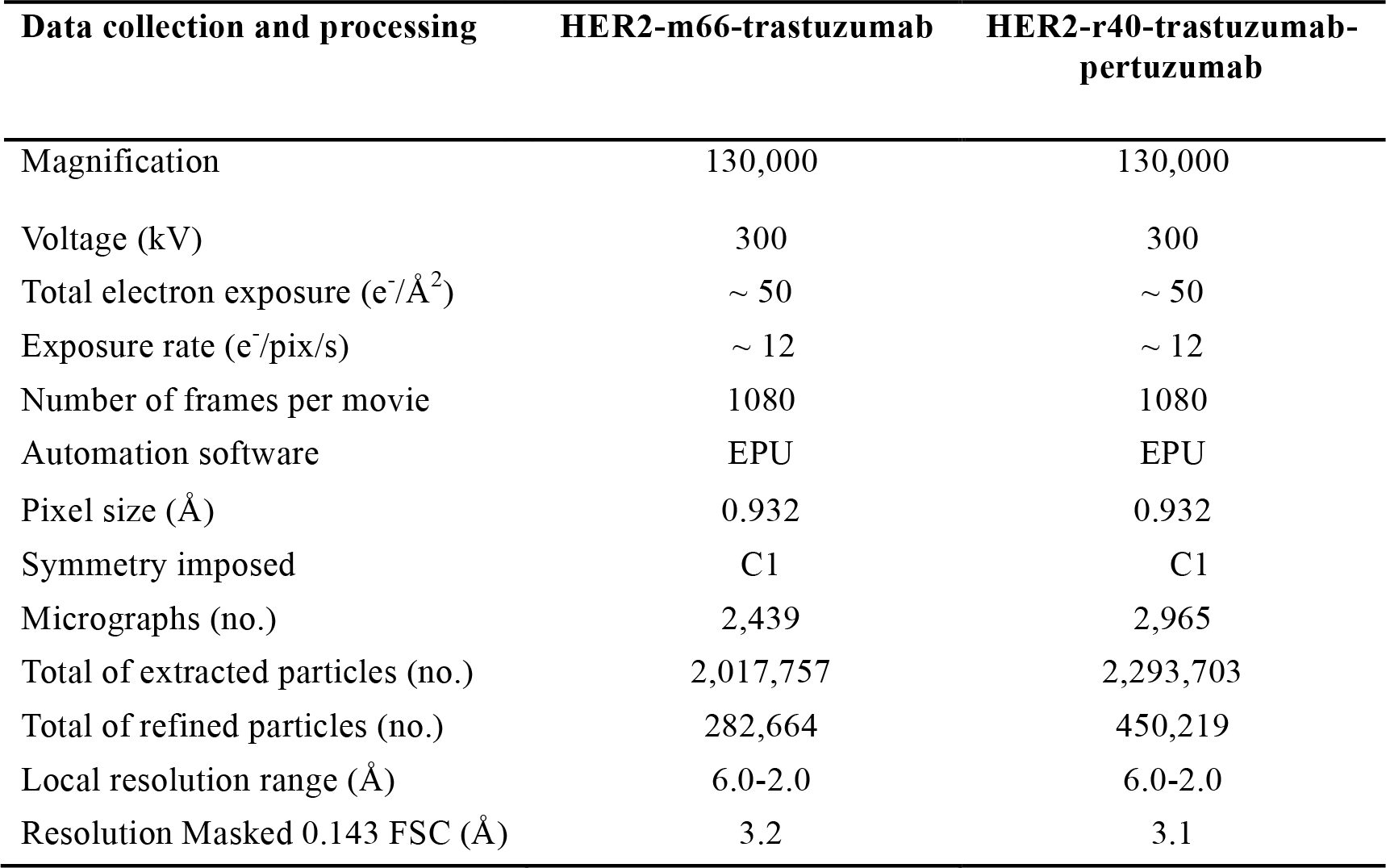

Supplement: S1 Table — (PNG) [file pone.0338127.s007.png]

Fig. 4A

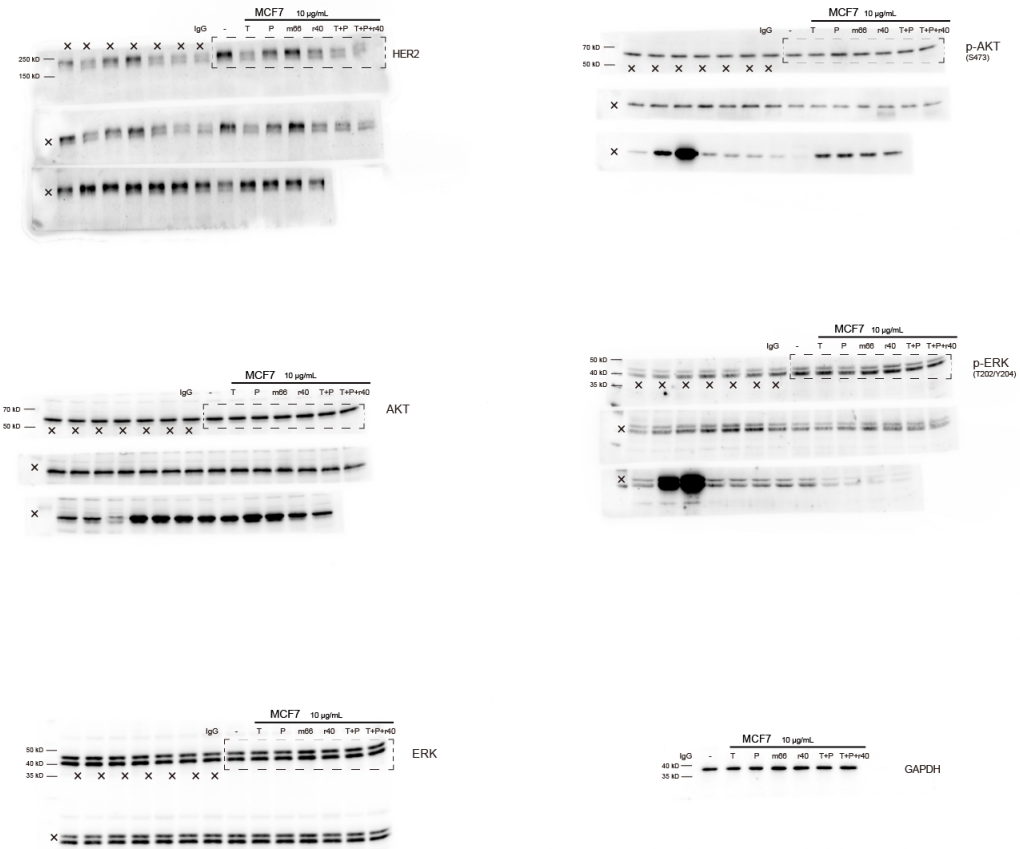

Fig. 4B

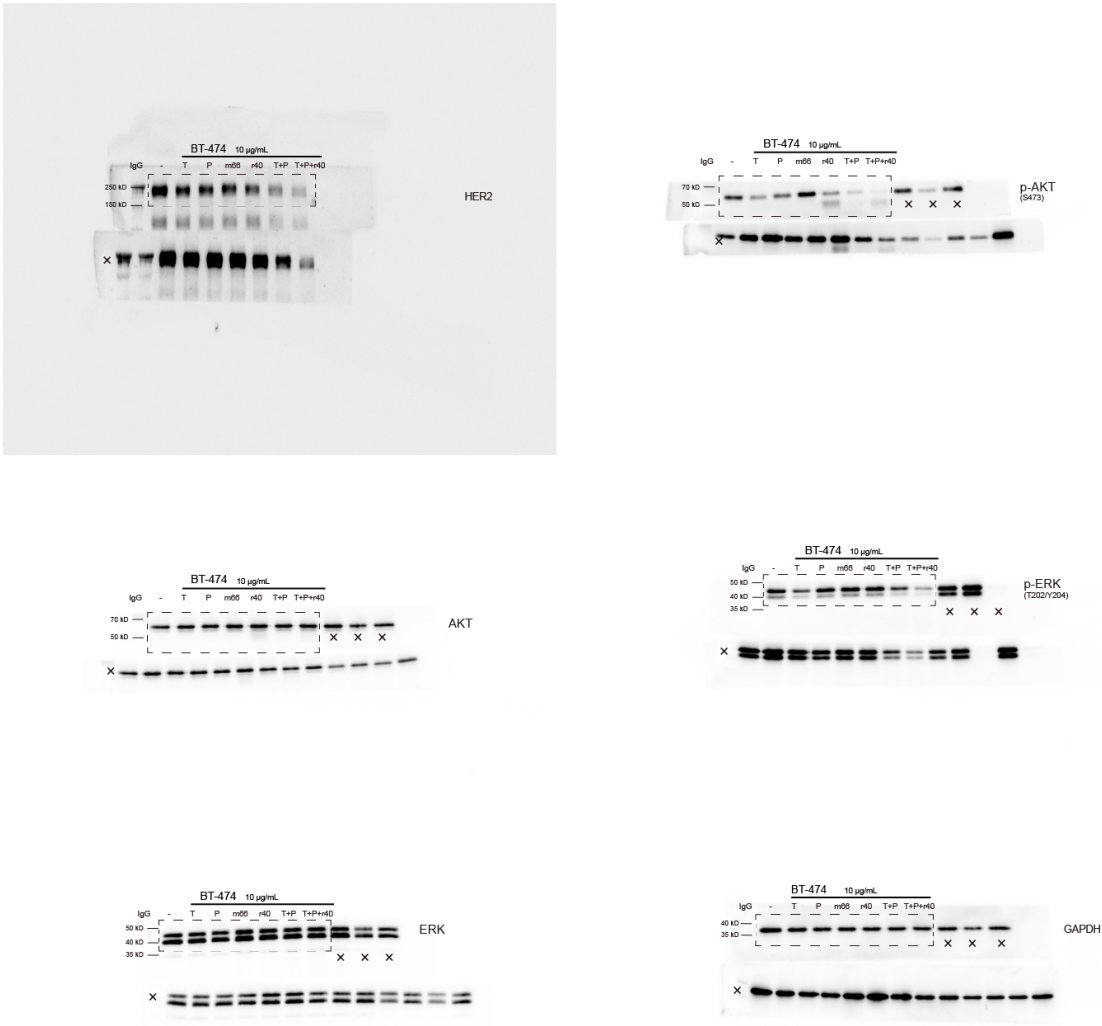

Fig. 4C

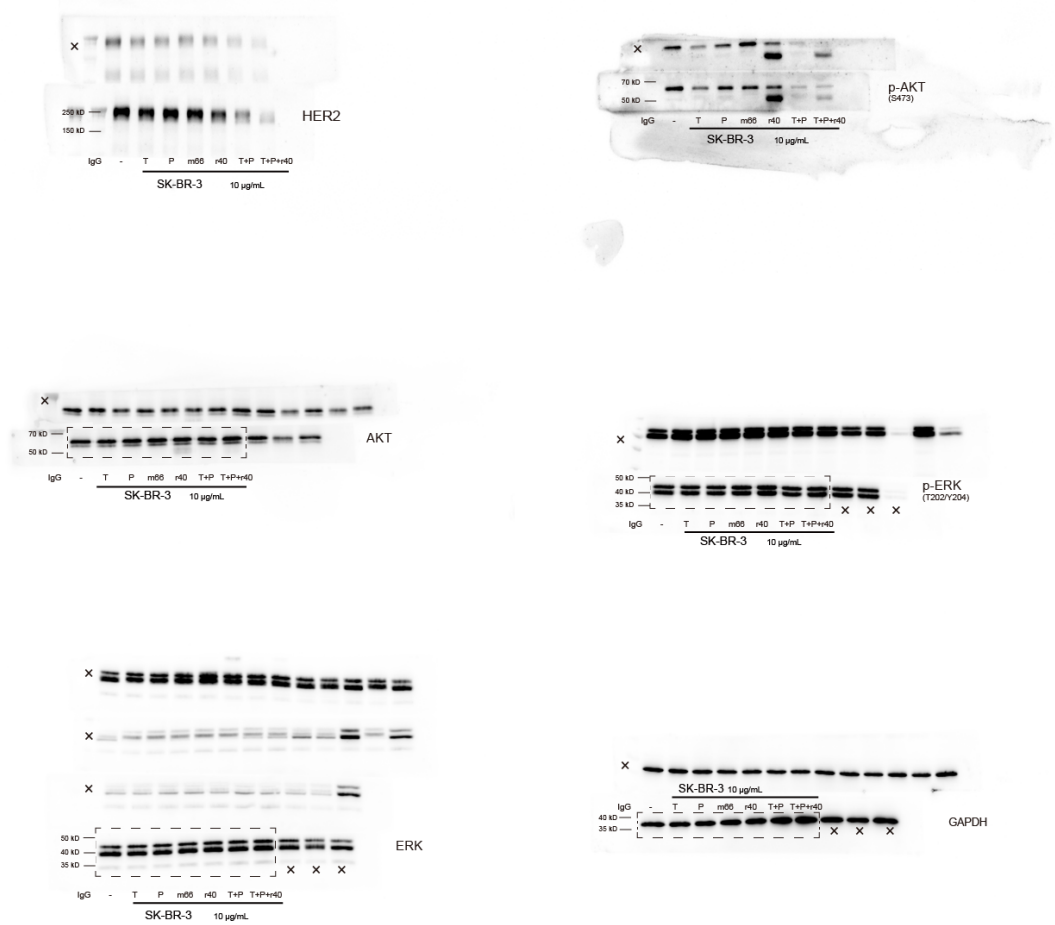

Fig. 5A

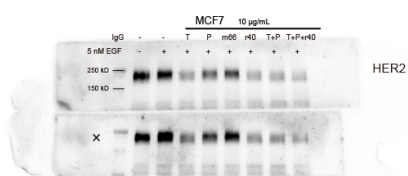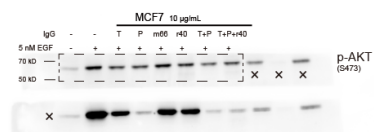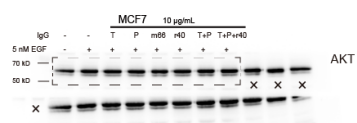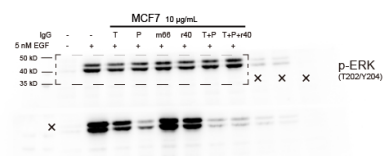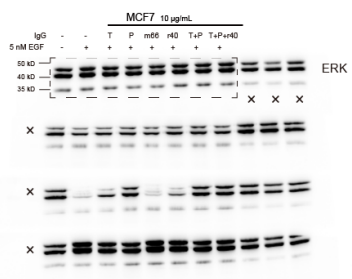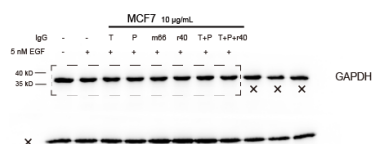

Fig. 5B

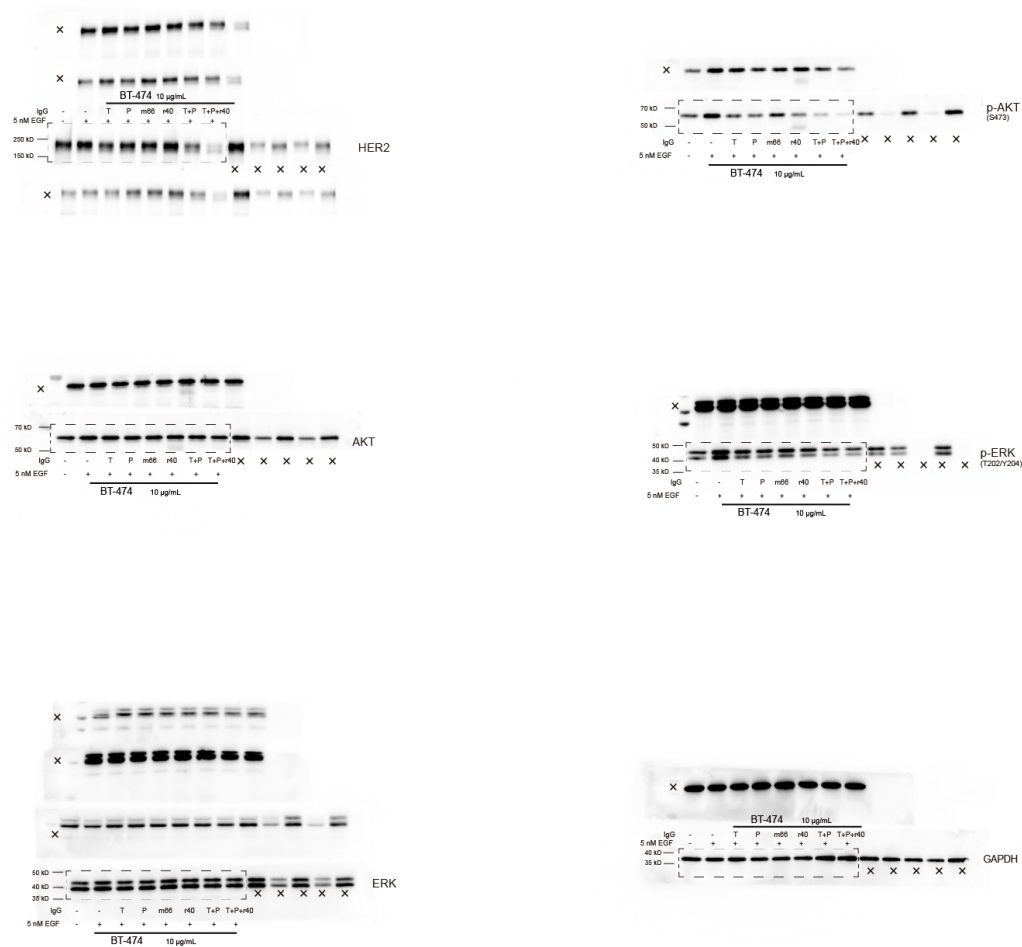

Fig. 5C

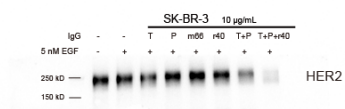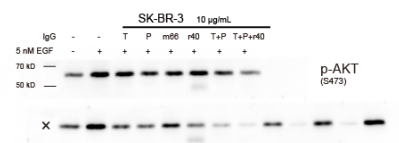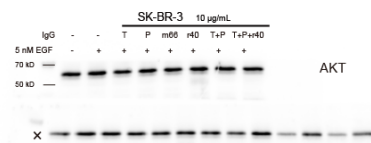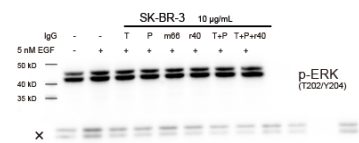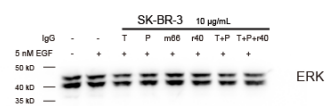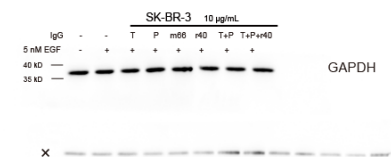

Fig. 5D

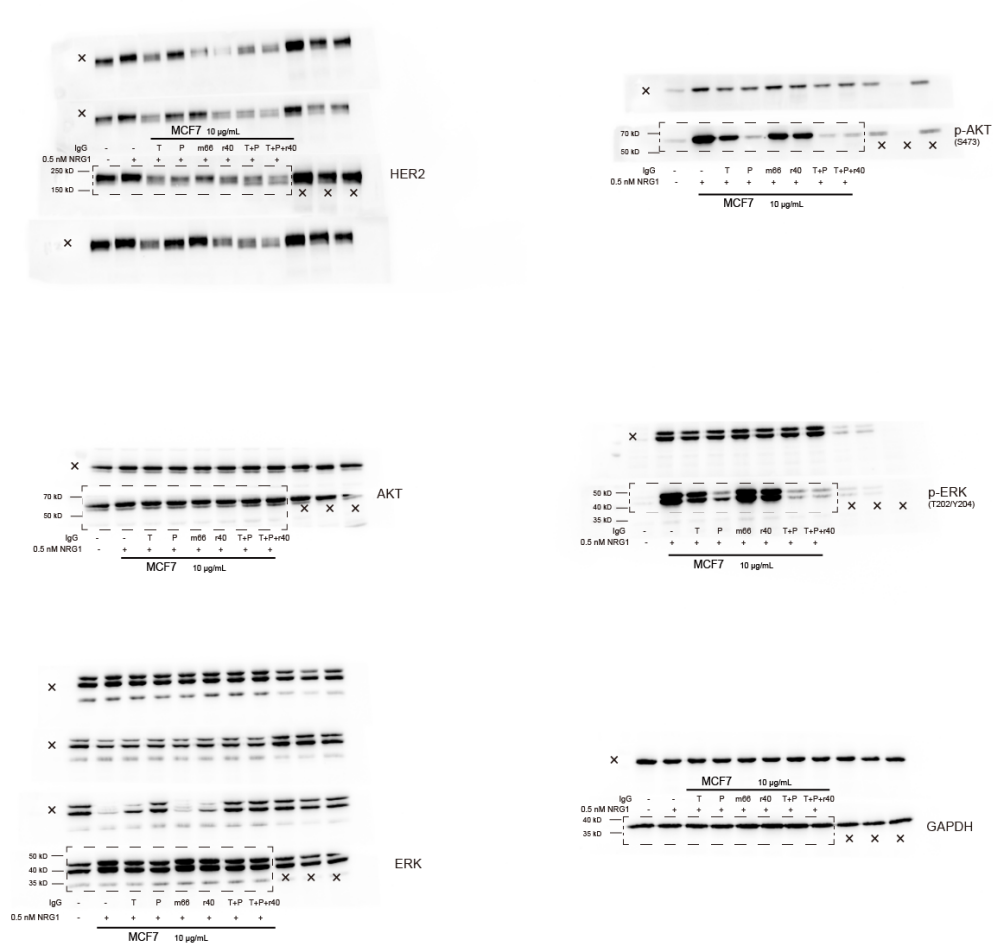

Fig. 5E

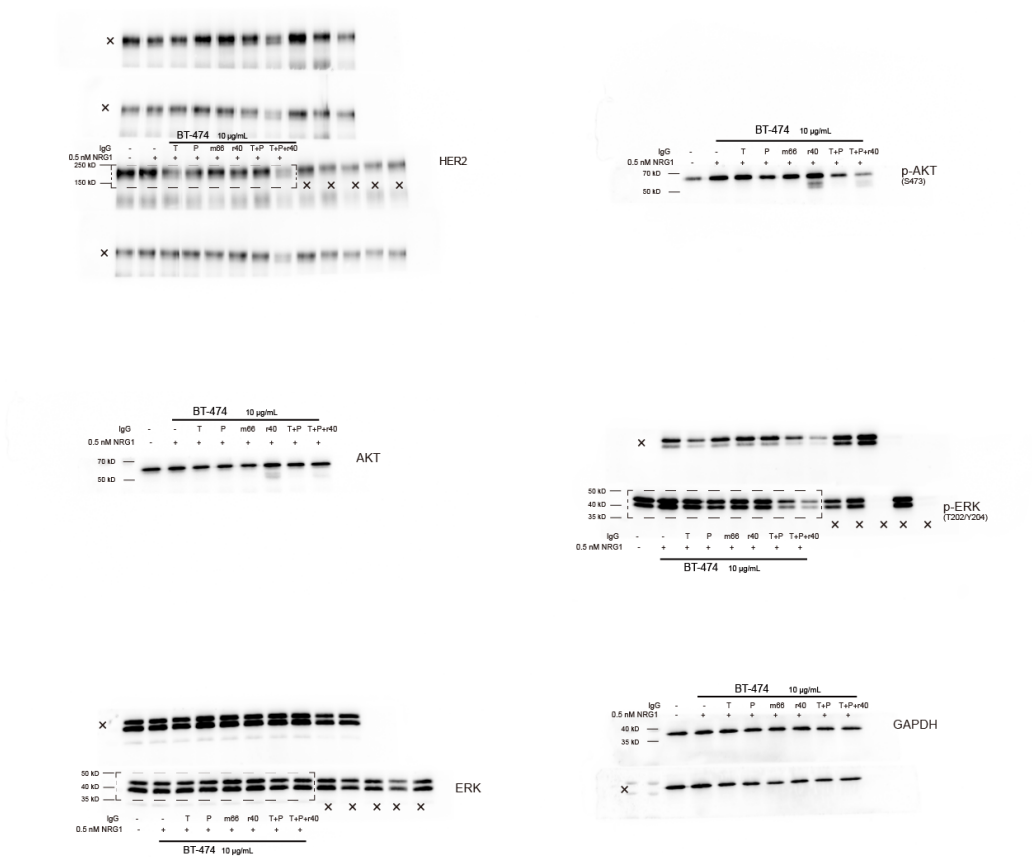

Fig. 5F

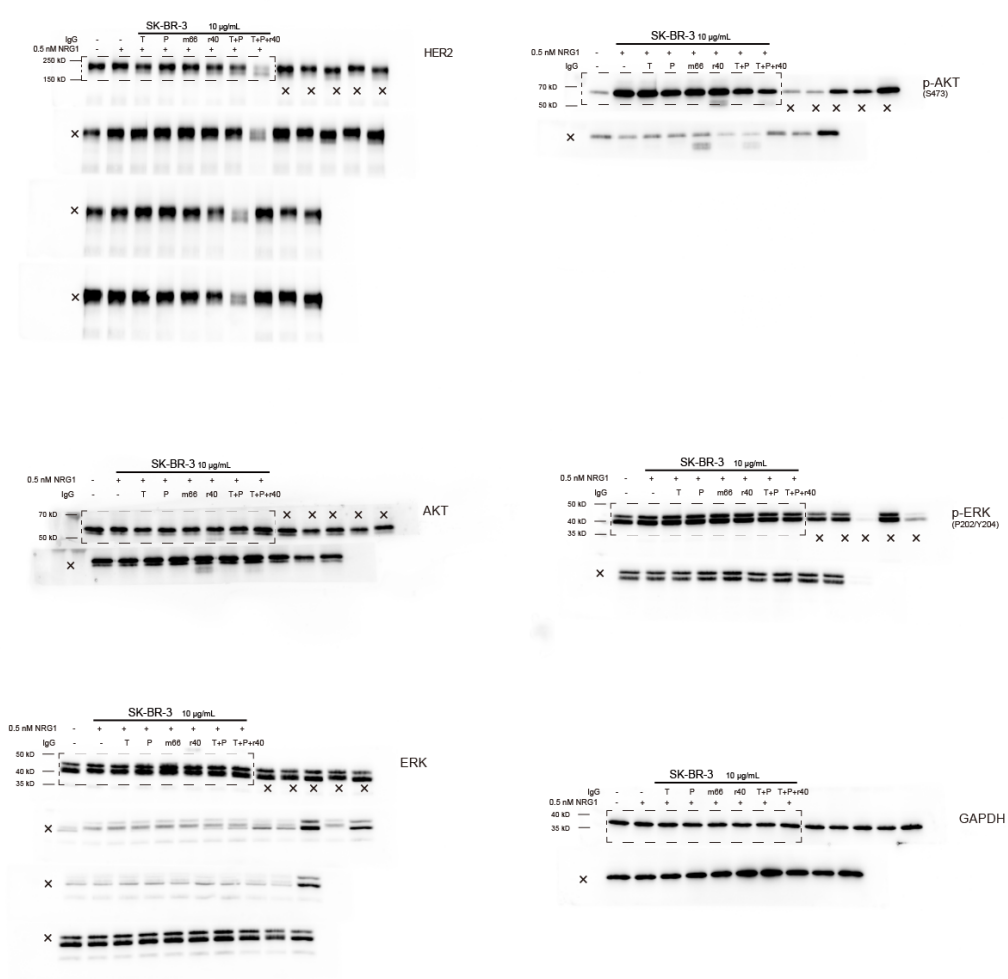

S1C Fig.

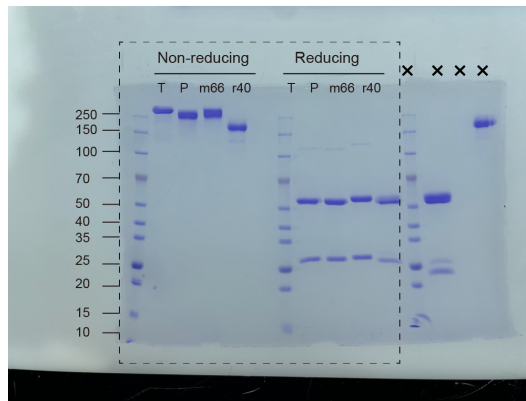

S1C Fig.

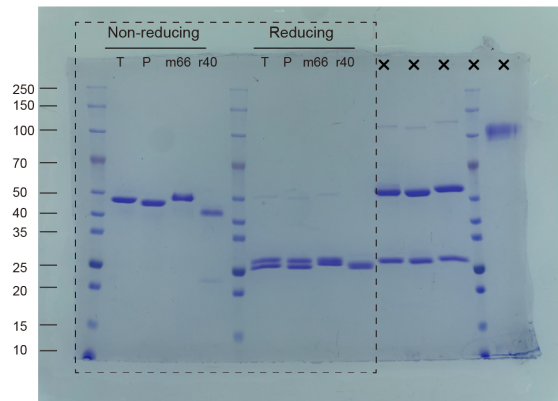

S1D Fig.

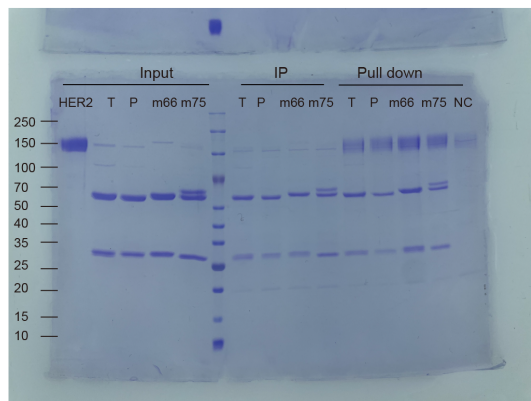

S1E Fig.

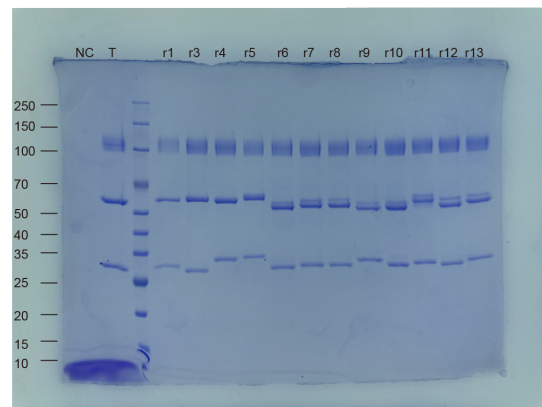

S1E Fig.

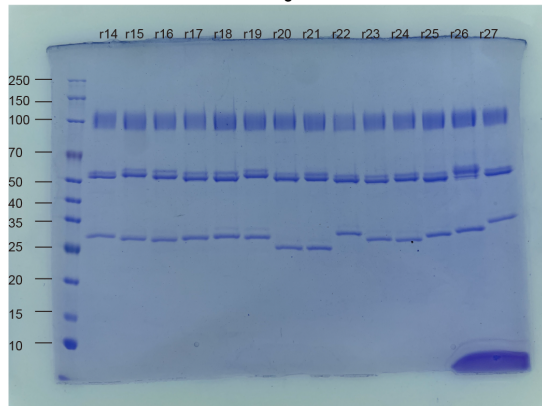

S1E Fig.

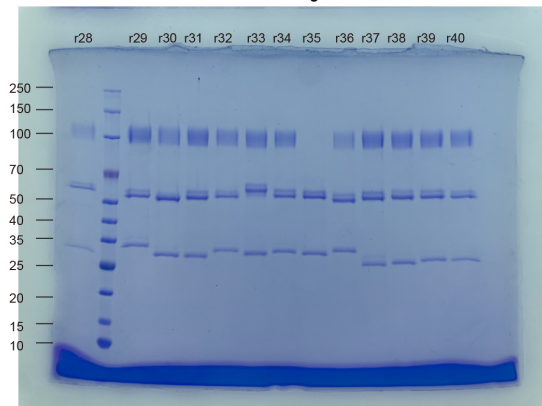

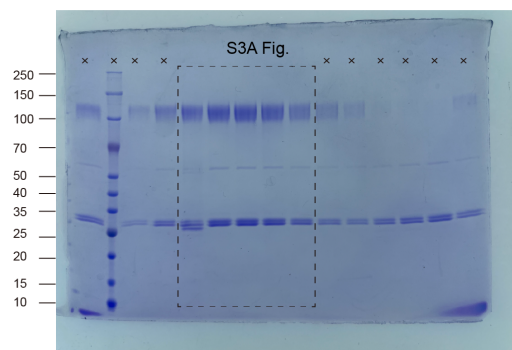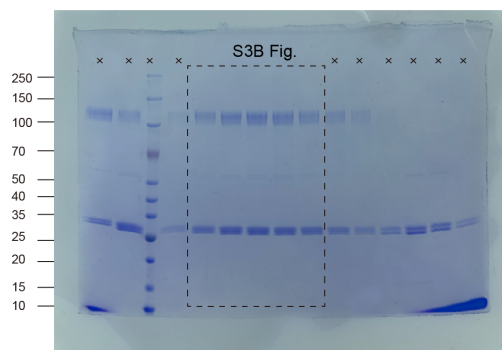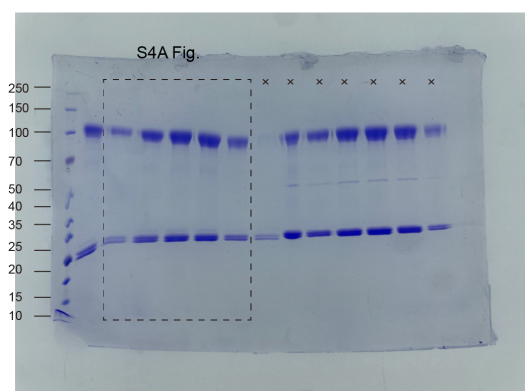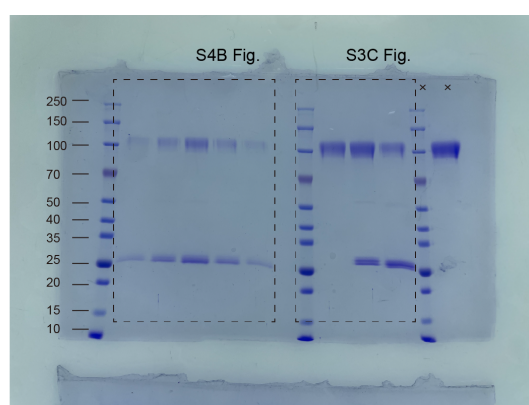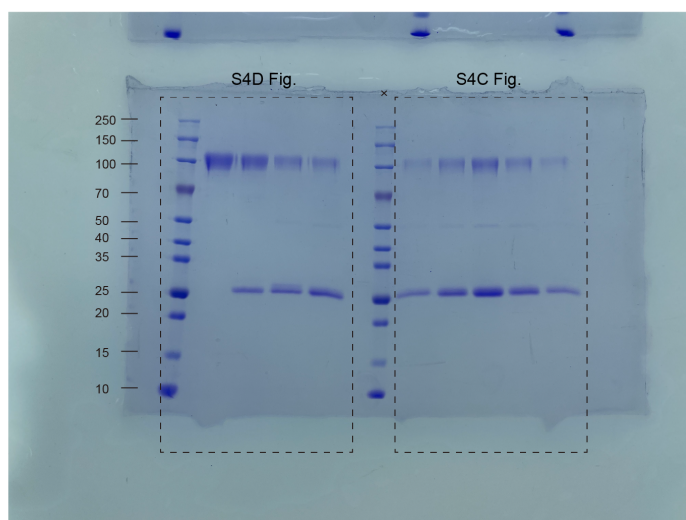

Supplement: S1 Raw Images — (PDF) [file pone.0338127.s009.pdf]
