# Supplementary material for: Inhibition of HER2 signaling and breast cancer cell growth with a novel antibody targeting HER2 ECD III/IV
Source: PLoS One. 2026 Jan 15;21(1):e0338127. doi: 10.1371/journal.pone.0338127 (PMC12806845; doi:10.1371/journal.pone.0338127)
Supplement: Attachment — Submitted filename: PONE-D-25-13465_R1_rev.pdf [file pone.0338127.s012.pdf]

# PLOS ONE

## Inhibition of HER2 signaling and breast cancer cell growth with a novel antibody targeting HER2 ECD III/IV

--Manuscript Draft--

| | |
|---|---|
| **Manuscript Number:** | PONE-D-25-13465R1 |
| **Article Type:** | Research Article |
| **Full Title:** | Inhibition of HER2 signaling and breast cancer cell growth with a novel antibody targeting HER2 ECD III/IV |
| **Short Title:** | Inhibition of breast cancer cell growth with a novel antibody |
| **Corresponding Author:** | Chunchun Liu<br>Fudan University Shanghai Cancer Center<br>Shanghai, CHINA |
| **Keywords:** | HER2, breast cancer, antibody, cryo-EM |
| **Abstract:** | Human epidermal growth factor receptor 2 (HER2), a ligand-independent tyrosine kinase receptor of the EGFR family, functions as a key oncogenic factor in breast, gastric and several other solid malignancies. Despite the huge survival benefits from anti-HER2 treatment, particularly in breast cancer and gastric cancer, the emergence of treatment resistance and cancer recurrence persists as a significant challenge. Thus, the creation of innovative antibodies exhibiting complementary effects to the current anti-HER2 therapies could offer clinical benefits. Here, we describe two novel HER2-targeting monoclonal antibodies, m66 (mouse-derived) and r40 (rabbit-derived), which inhibit breast cancer cell proliferation in vitro. Antibody r40 exhibits a more potent effect than m66 in suppressing the PI3K/AKT and MAPK signaling pathways. Compared with the combination of trastuzumab and pertuzumab, the addition of r40 significantly enhanced the inhibitory effect. Furthermore, we report the cryo-EM structure determination of HER2-m66-trastuzumab ternary complex and HER2-r40-trastuzumab-pertuzumab tetrameric complex to overall 3.2 Å and 3.1 Å resolution, respectively. The structure data indicate that the epitope of m66 overlaps with that of pertuzumab, whereas the epitope of r40 lies within the HRE2 ECD III/IV region, distinct from the epitopes of trastuzumab and pertuzumab. The findings of this study highlight that r40 is a promising candidate for combination therapy with trastuzumab and pertuzumab in breast cancer treatment. |
| **Order of Authors:** | Chunchun Liu |
| | Yulei Ren |
| | Xuan Luo |
| | Peiyun Zhou |
| | Xiangjuan Du |
| | Qianmin Wang |
| | Xue Han |
| | Yanhui Xu |
| | Ping Wang |
| | Dan Zhao |
| | Huirong Yang |
| **Response to Reviewers:** | Thanks for the reviewers’ and editors' constructive comments and suggestion, we have significantly improved the manuscript by performing critical experiments and refining the manuscript, resulting in a substantial enhancement in quality. The main point is: the p-AKT level and HER2 protein level is significantly decreased with triple combination (trastuzumab-pertuzumab-r40) use in breast cancer cells, which shows that r40 is a promising candidate to augment existing HER2-targeted therapies, potentially improving outcomes for HER2-overexpressing cancers. Future work should |

Powered by Editorial Manager® and ProduXion Manager® from Aries Systems Corporation

| | |
|---|---|
| | include detailed mechanistic analyses and humanization of r40. |
| **Additional Information:** | |
| **Question** | **Response** |
| **Financial Disclosure**<br><br>Enter a financial disclosure statement that describes the sources of funding for the work included in this submission. Review the submission guidelines for detailed requirements. View published research articles from *PLOS ONE* for specific examples.<br><br>This statement is required for submission and **will appear in the published article** if the submission is accepted. Please make sure it is accurate.<br><br>**Funded studies**<br>Enter a statement with the following details:<br>• Initials of the authors who received each award<br>• Grant numbers awarded to each author<br>• The full name of each funder<br>• URL of each funder website<br>• Did the sponsors or funders play any role in the study design, data collection and analysis, decision to publish, or preparation of the manuscript?<br><br>Did you receive funding for this work? | Yes |
| Please add funding details.<br>as follow-up to "**Financial Disclosure**<br><br>Enter a financial disclosure statement that describes the sources of funding for the work included in this submission. Review the submission guidelines for detailed requirements. View published research articles from *PLOS ONE* for specific examples.<br><br>This statement is required for submission and **will appear in the published article** if the submission is accepted. Please make sure it is accurate. | Shanghai Natural Science Foundation of China (21ZR1407900) |

| | |
|---|---|
| **Funded studies**<br>Enter a statement with the following details:<br>• Initials of the authors who received each award<br>• Grant numbers awarded to each author<br>• The full name of each funder<br>• URL of each funder website<br>• Did the sponsors or funders play any role in the study design, data collection and analysis, decision to publish, or preparation of the manuscript?<br><br>Did you receive funding for this work?" | |
| Please select the country of your main research funder (please select carefully as in some cases this is used in fee calculation).<br>as follow-up to "**Financial Disclosure**<br><br>Enter a financial disclosure statement that describes the sources of funding for the work included in this submission. Review the submission guidelines for detailed requirements. View published research articles from *PLOS ONE* for specific examples.<br><br>This statement is required for submission and **will appear in the published article** if the submission is accepted. Please make sure it is accurate.<br><br>**Funded studies**<br>Enter a statement with the following details:<br>• Initials of the authors who received each award<br>• Grant numbers awarded to each author<br>• The full name of each funder<br>• URL of each funder website<br>• Did the sponsors or funders play any role in the study design, data collection and analysis, decision to publish, or preparation of the manuscript?<br><br>Did you receive funding for this work?" | CHINA - CN |
| **Competing Interests** | The authors have declared that no competing interests exist. |

| | |
|---|---|
| Use the instructions below to enter a competing interest statement for this submission. On behalf of all authors, disclose any competing interests that could be perceived to bias this work—acknowledging all financial support and any other relevant financial or non-financial competing interests.<br><br>This statement is required for submission and **will appear in the published article** if the submission is accepted. Please make sure it is accurate and that any funding sources listed in your Funding Information later in the submission form are also declared in your Financial Disclosure statement.<br><br>View published research articles from *PLOS ONE* for specific examples.<br><br>**NO authors have competing interests**<br><br>Enter: *The authors have declared that no competing interests exist.*<br><br>**Authors with competing interests**<br><br>Enter competing interest details beginning with this statement:<br><br>*I have read the journal's policy and the authors of this manuscript have the following competing interests: [insert competing interests here]*<br><br>* typeset | |
| **Ethics Statement**<br><br>Enter an ethics statement for this submission. This statement is required if the study involved:<br><br>• Human participants<br>• Human specimens or tissue<br>• Vertebrate animals or cephalopods<br>• Vertebrate embryos or tissues | N/A |

Powered by Editorial Manager® and ProduXion Manager® from Aries Systems Corporation

| | |
|---|---|
| • Field research<br><br>Write "N/A" if the submission does not require an ethics statement.<br><br>General guidance is provided below. Consult the submission guidelines for detailed instructions. **Make sure that all information entered here is included in the Methods section of the manuscript.** | |

| | |
|---|---|
| **Format for specific study types**<br><br>**Human Subject Research (involving human participants and/or tissue)**<br>• Give the name of the institutional review board or ethics committee that approved the study<br>• Include the approval number and/or a statement indicating approval of this research<br>• Indicate the form of consent obtained (written/oral) or the reason that consent was not obtained (e.g. the data were analyzed anonymously)<br><br>**Animal Research (involving vertebrate animals, embryos or tissues)**<br>• Provide the name of the Institutional Animal Care and Use Committee (IACUC) or other relevant ethics board that reviewed the study protocol, and indicate whether they approved this research or granted a formal waiver of ethical approval<br>• Include an approval number if one was obtained<br>• If the study involved *non-human primates*, add *additional details* about animal welfare and steps taken to ameliorate suffering<br>• If anesthesia, euthanasia, or any kind of animal sacrifice is part of the study, include briefly which substances and/or methods were applied<br><br>**Field Research**<br><br>Include the following details if this study involves the collection of plant, animal, or other materials from a natural setting:<br>• Field permit number<br>• Name of the institution or relevant body that granted permission | |
| **Data Availability**<br><br>Authors are required to make all data underlying the findings described fully available, without restriction, and from the time of publication. PLOS allows rare exceptions to address legal and ethical concerns. See the PLOS Data Policy and FAQ for detailed information. | Yes - all data are fully available without restriction |

| | |
|---|---|
| A Data Availability Statement describing where the data can be found is required at submission. Your answers to this question constitute the Data Availability Statement and **will be published in the article**, if accepted.<br><br>**Important:** Stating 'data available on request from the author' is not sufficient. If your data are only available upon request, select 'No' for the first question and explain your exceptional situation in the text box.<br><br>Do the authors confirm that all data underlying the findings described in their manuscript are fully available without restriction? | |
| **Describe where the data may be found in full sentences. If you are copying our sample text, replace any instances of XXX with the appropriate details.**<br><br>• If the data are **held or will be held in a public repository**, include URLs, accession numbers or DOIs. If this information will only be available after acceptance, indicate this by ticking the box below. For example: *All XXX files are available from the XXX database (accession number(s) XXX, XXX.*).<br>• If the data are all contained **within the manuscript and/or Supporting Information files**, enter the following: *All relevant data are within the manuscript and its Supporting Information files.*<br>• If neither of these applies but you are able to provide **details of access elsewhere**, with or without limitations, please do so. For example:<br><br>*Data cannot be shared publicly because of [XXX]. Data are available from the XXX Institutional Data Access / Ethics Committee (contact via XXX) for researchers who meet the criteria for access to confidential data.*<br><br>*The data underlying the results presented in the study are available from (include the name of the third party* | All relevant data are within the manuscript and its Supporting Information files. |

| | |
|---|---|
| *and contact information or URL).*<br>• This text is appropriate if the data are owned by a third party and authors do not have permission to share the data.<br><br>* typeset | |
| Additional data availability information: | |

Powered by Editorial Manager® and ProduXion Manager® from Aries Systems Corporation

Manuscript Click here to access/download;Manuscript;Manuscript.docx

# Inhibition of HER2 signaling and breast cancer cell growth with a novel antibody targeting HER2 ECD III/IV

Chunchun Liu [1, ¶, *], Yulei Ren [1, ¶], Xuan Luo [3, 4, ¶], Peiyun Zhou [1], Xiangjuan Du [1], Qianmin Wang [1], Xue Han [1], Yanhui Xu [1, 2], Ping Wang [6], Dan Zhao [1, 5, *], Huirong Yang [1, 2, *]

[1] Fudan University Shanghai Cancer Center, Institutes of Biomedical Sciences, Shanghai Key Laboratory of Radiation Oncology, and Shanghai Key Laboratory of Medical Epigenetics, Shanghai Medical College of Fudan University, Shanghai, China.

[2] The International Co-laboratory of Medical Epigenetics and Metabolism, Ministry of Science and Technology, China, Department of Systems Biology for Medicine, School of Basic Medical Sciences, Shanghai Medical College of Fudan University, Shanghai, China.

[3] Key Laboratory of Breast Cancer in Shanghai, Department of Breast Surgery, Fudan University Shanghai Cancer Center, Shanghai Medical College, Fudan University, Shanghai, China

[4] Department of Oncology, Shanghai Medical College, Fudan University, Shanghai, China

[5] School of Life Sciences, Greater Bay Area Institute of Precision Medicine (Guangzhou), Fudan University, Guangzhou, China

[6] Molinsights Biotechnologies, Guangdong Medical Valley Life Science Park, Guangzhou, China.

* Corresponding author

E-mail: liuchun880616@yeah.net, danzhao5278@foxmail.com, yanghr@fudan.edu.cn

¶These authors contributed equally to this work.

## Abstract

Human epidermal growth factor receptor 2 (HER2), a ligand-independent tyrosine kinase receptor of the EGFR family, functions as a key oncogenic factor in breast, gastric and several other solid malignancies. Despite the huge survival benefits from anti-HER2 treatment, particularly in breast cancer and gastric cancer, the emergence of treatment resistance and cancer recurrence persists as a significant challenge. Thus, the creation of innovative antibodies exhibiting complementary effects to the current anti-HER2 therapies could offer clinical benefits. Here, we describe two novel HER2-targeting monoclonal antibodies, m66 (mouse-derived) and r40 (rabbit-derived), which inhibit breast cancer cell proliferation *in vitro*. Antibody r40 exhibits a more potent effect than m66 in suppressing the PI3K/AKT and MAPK signaling pathways. Compared with the combination of trastuzumab and pertuzumab, the addition of r40 significantly enhances the inhibitory effect. Furthermore, we report the cryo-EM structure determination of HER2-m66-trastuzumab ternary complex and HER2-r40-trastuzumab-pertuzumab tetrameric complex to overall 3.2 Å and 3.1 Å resolution, respectively. The structure data indicate that the epitope of m66 overlaps with that of pertuzumab, whereas the epitope of r40 lies within the HRE2 ECD III/IV region, distinct from the epitopes of trastuzumab and pertuzumab. The findings of this study highlight that r40 is a promising candidate for combination therapy with trastuzumab and pertuzumab in breast cancer treatment.

## Introduction

The human epidermal growth factor receptor (EGFR) family comprises four members: EGFR (ERBB1, HER1), HER2 (ERBB2), HER3 (ERBB3) and HER4 (ERBB4). All of these HER proteins are oncogenic drivers, with EGFR and HER2 highly associated with tumorigenesis. HER2, characterized as a single-pass type I transmembrane protein, consists of four extracellular domains (ECD I-IV), a transmembrane domain and an intracellular tyrosine kinase domain from N-to-C terminus. Unlike other EGFR family members, HER2 has the constitutive activity that can initiate signaling pathways without a bound activating ligand[1, 2]. Overexpression of HER2 results in ligand-independent homodimerization and ligand-dependent heterodimerization of EGFR/HER2, HER2/HER3, HER2/HER4, causing HER2 phosphorylation, specifically activating the PI3K/AKT and MAPK signaling pathways, thus triggering cell division and growth[1, 3].

Current clinical practice guidelines incorporate various anti-HER2 biologics, ranging from monoclonal antibodies to advanced antibody-drug conjugates (ADC) platforms, for managing cancers with amplified HER2 expression. Trastuzumab (Herceptin®) was approved for the therapeutic treatment of breast cancer and metastatic gastric cancer in patients with HER2 overexpression in 1998 and 2010, respectively. Trastuzumab binds to ECD IV of HER2, and triggers the internalization and degradation of HER2. This results in the inhibition of downstream signaling pathways and the elimination of cytotoxic effector T cells antibody-dependent cell-mediated cytotoxicity (ADCC) mechanisms[4-6]. Combined with chemotherapy, trastuzumab has demonstrated significant therapeutic benefits in the treatment of HER2-driven breast malignancies. However, therapeutic resistance and disease relapse inevitably occurs after trastuzumab treatment[7]. Pertuzumab (Perjeta®) is the second approved anti-HER2 monoclonal antibody drug that binds to ECD II of HER2[8, 9]. The binding epitopes of trastuzumab and pertuzumab are both situated at the dimerization interface of HER2[10]. Combinational use of trastuzumab and pertuzumab has been evaluated to exhibit enhanced antitumor effect for HER2 overexpressing breast cancer[11-13].

Other anti-HER2 agents, including small molecular drugs (lapatinib, neratinib), and ADC-based drugs (trastuzumab emtansine, trastuzumab deruxtecan) have been approved for HER2-overexpression breast cancers[14-18]. Trastuzumab and

trastuzumab deruxtecan (T-Dxd) are also approved for the therapy of HER2-positive gastric cancers[19-21]. Moreover, metastatic HER2-driven malignancies inevitably develop refractoriness to the current approved HER2 therapies, leading to disease progression over time[22].

The observed synergistic anticancer effect of trastuzumab and pertuzumab targeting topographically distinct HER2 epitopes has catalyzed intensive exploration of novel antibodies recognizing new epitopes[23]. The combination of three anti-HER2 antibodies with non-overlapping epitopes showed higher antitumor efficacy than trastuzumab and pertuzumab combination[24]. Also, the combination of a monoclonal antibody targeting HER2 ECD III with trastuzumab and pertuzumab provided blockade of HER2 heterodimerization and cell signaling[25]. Although neither ECD I nor III is directly involved in HER2 dimerization, ECD I is critical for forming a binding pocket for the dimerization arm of other EGFR family members. ECD III, situated in the center of the whole extracellular domain, serves as a pivotal connector between ECD II and ECD IV, and establishes contact with ECD I. Antibodies targeting ECD I and III can also modulate the conformational dynamics of the HER2 receptor, which in turn could impede the activation cascade of HER2 signaling[23].

In this study, we report two novel HER2 targeted antibodies, m66 (mouse-derived) and r40 (rabbit-derived). The HER2 ECD III/IV specific monoclonal rabbit antibody r40 suppresses breast cancer cell growth by inhibiting the PI3K/AKT and MAPK signaling pathways. Furthermore, r40 enhances the efficacy of trastuzumab-pertuzumab combination against breast cancer cell proliferation and HER2 signaling inhibition.

# Results

## Discovery of antibodies targeting HER2 ECD

HER2 ECD specific antibodies were obtained by single cell VDJ sequencing (scVDJ-seq) from the spleens of mice and rabbits immunized with HER2 ECD proteins (S1A Fig). The antibodies selected from B cells were synthesized and expressed *in vitro* and assessed for antigen binding affinity by ELISA and pull-down assays. Finally, we acquired two mouse antibodies (termed m66, m75) and thirty-eight rabbit antibodies among r1-r40 specifically targeting HER2 ECD.

## Antibody m66 competes with pertuzumab for binding to HER2

To characterize the epitopes of the two mouse antibodies, we purified trastuzumab, pertuzumab, m66, and m75 in two human IgG1 formats, His-tagged and non-His-tagged forms (S1B Fig). We prepared these two formats by performing the purification procedure without or with HRV 3C protease cleavage. Blocking ELISA was performed to assess the binding epitopes by incubating immobilized HER2 with supersaturating non-His-tagged IgGs followed by detecting the binding of His-tagged IgGs. As expected, each non-His-tagged IgG significantly blocked the binding of its own His-tagged IgG (Fig 1A). In addition, supersaturation of m66 and m75 did not influence the binding of trastuzumab, indicating that m66 and m75 target epitopes distinct from that of trastuzumab. The binding of antibody m66 was blocked by pertuzumab but not vice versa, indicating that the binding affinity of m66 was weaker than that of pertuzumab. While antibody m75 blocked pertuzumab and pertuzumab blocked m75 (Fig 1A). These results indicated that m66 and m75 bound to distinct epitopes from that of trastuzumab, while they likely bind to epitopes overlapping with pertuzumab.

To map antibody epitope by flow cytometry, we selected the low-HER2-expressing breast cancer cell line MCF7. This model allowed us to determine whether the identified mouse and rabbit antibodies retained binding to HER2 in the presence of saturating concentrations of trastuzumab and pertuzumab. To determine the saturation concentrations of trastuzumab and pertuzumab in MCF7 cells, we incubated the cells with 0–400 nM of each antibody individually. After incubation, cells were stained with an AF647-conjugated goat anti-human secondary antibody (Invitrogen, A21445) and

analyzed by flow cytometry. The saturation concentration for both antibodies was determined to be about 100 nM (Fig 1B).

Subsequently, trastuzumab, pertuzumab, m66 and m75 were conjugated with NHS-AF647 dye. As expected, binding of AF647-conjugated trastuzumab and pertuzumab was precluded in MCF7 cells saturated with unlabeled trastuzumab and pertuzumab (Fig 1C). Binding of both m66-AF647 and m75-AF647 was blocked exclusively by pertuzumab saturation, with minimal inhibition from trastuzumab saturation (Fig 1D). This also indicated that m66 and m75 bound to epitopes overlapping with pertuzumab, which was consistent with ELISA results (Fig 1A). Enhanced binding affinity of m66 to trastuzumab-saturated MCF7 cells suggested that trastuzumab binding altered HER2 conformation, thus increasing m66 affinity (Fig 1D). While in the purification of antibodies, we found that m75 was unstable and prone to precipitation, antibody m66 was selected for further investigation.

## Antibody r40 binds to a different epitope from that of trastuzumab and pertuzumab

To characterize rabbit antibody epitopes through competitive binding, we pre-incubated Expi 293 cells with 10 μg/mL (66.7 nM) trastuzumab and pertuzumab. Then the cells were incubated with rabbit antibodies and analyzed by flow cytometry assay to assess simultaneous binding capacity. Primarily, we tested the binding ability of 39 rabbit antibodies in Expi 293 cells, among which r22, r30, r35 could not recognize cell surface HER2 (S2A Fig). When cells were pre-incubated with trastuzumab and pertuzumab, the mean fluorescence intensity (MFI) of the rabbit monoclonal antibody remained comparable to control cells incubated with the rabbit antibody alone. This indicated that the rabbit antibody bound HER2 without competing for the epitopes of trastuzumab and pertuzumab. We therefore determined that the six rabbit antibodies (r3, r4, r5, r29, r36 and r40) recognized epitopes distinct from those of trastuzumab and pertuzumab (S2B Fig).

We further comfirmed the distinct epitope binding of rabbit monoclonal antibodies (r3, r4, r5, r29, r35, r36, r40) relative to trastuzumab and pertuzumab in low-HER2-expressing MCF7 cells (Fig 1E, S2C Fig). Saturation of HER2 in MCF7 cells with

trastuzumab and pertuzumab did not inhibit subsequent binding of r40, comfirming its epitope was distinct from those targeted by these two therapeutic antibodies (Fig 1E). Similarly, binding of r3, r4, r5 and r29 remained unaffected, consistent with their epitope divergence from those of trastuzumab and pertuzumab (S2C Fig). In contrast, r36 exhibited increased MFI on trastuzumab-pertuzumab-saturated cells, indicating the binding of the two therapeutic antibodies altered HER2 conformation to enhance r36 affinity (S2C Fig).

## Antibody r40 inhibits breast cancer cell proliferation

Next, we evaluated the anti-proliferative activity of six rabbit antibodies (r3, r4, r5, r29, r36, r40) and the mouse antibody m66 in HER2-overexpressing BT-474 breast cancer cells (Fig 2A). Among the tested antibodies, r40 showed concentration-dependent suppression of cell viability, exceeding the potency of both trastuzumab and pertuzumab (Fig 2A). The triple antibody combination (trastuzumab, pertuzumab and r40) enhanced inhibition at concentrations below 10 nM compared to the dual combination (trastuzumab and pertuzumab) or single agents (Fig 2A). While the other five rabbit antibodies and the mouse antibody m66 exhibited weak effects on proliferation (Fig 2A). Based on these findings, r40 and m66 were selected for further characterization.

In another two breast cancer cell lines, HER2-overexpressing SK-BR-3 and low-HER2-expressing MCF7, r40 demonstrated comparable growth inhibition to trastuzumab and pertuzumab (Fig 2B). The triple combination exhibited enhanced or comparable efficacy relative to the dual combination (Fig 2B).

The binding kinetics of trastuzumab, pertuzumab, m66 and r40 with HER2 were evaluated by Surface Plasmon Resonance Imaging (SPRi), which showed high affinity for r40 ($K_D$ = 0.279 nM) and trastuzumab ($K_D$ = 0.283 nM), slightly lower binding affinity for pertuzumab ($K_D$ = 2.3 nM), and modest affinity for m66 ($K_D$ = 86.4 nM) (Fig 2C).

## Antibody r40 and m66 could bind and detect cell-surface HER2 of breast cancer cells

Next, we evaluated the binding capacity of m66 and r40 to the endogenous HER2 protein in breast cancer cells by flow cytometry. Both antibodies detected HER2 at

levels comparable to trastuzumab and pertuzumab as measured by MFI (Fig 3A-3C).

**Antibody r40 effectively blocks HER2-dependent signaling pathways**

PI3K/AKT and MAPK are the main signaling pathways downstream of the EGFR family[1, 23]. Both trastuzumab and pertuzumab have been reported to inhibit these two signaling pathways in the breast cancer cells[12, 26-28]. We evaluated some key downstream signaling molecules level upon treatment with antibodies for 24 h. We first assessed HER2 signaling pathway modulation in MCF7 cells after 24 h antibody treatment (5, 10, 25, 50 μg/mL) (Fig 4A). While trastuzumab, r40, trastuzumab-pertuzumab dual therapy, and triple combination all significantly reduced HER2 protein levels, neither p-AKT (S473) nor p-ERK (T202/Y204) showed significant changes (Fig 4A). Consistent with literatures, evaluating PI3K/AKT and MAPK pathways in MCF7 cells typically requires ligand stimulation (e.g., EGF or NRG1) [26, 29, 30]. Since antibodies at concentrations ranging from 5 to 50 μg/mL yielded comparable effects in reducing HER2 protein levels, we selected the concentration of 10 μg/mL (66.7 nM) for treating cells in subsequent experiments.

In BT-474 cells, trastuzumab, pertuzumab and r40 treatment reduced p-AKT (S473) levels (Fig 4B). Neither m66 nor r40 affected p-ERK (T202/Y204), while the triple antibody combination exhibited the lowest levels of p-AKT (S473) and p-ERK (T202/Y204) (Fig 4B).

In SK-BR-3 cells, r40 reduced HER2 protein levels, and the triple antibody combination achieved the lowest HER2 protein levels and exhibited a p-AKT (S473) effect comparable to trastuzumab and pertuzumab dual combination (Fig 4C).

In conclusion, in the absence of ligand activation, r40 demonstrated efficacy comparable to trastuzumab in disrupting constitutive HER2 homodimerization and heterodimerization in breast cancer cell lines. Furthermore, adding r40 to trastuzumab and pertuzumab enhanced their effect.

We next measured EGF-induced EGFR/HER2 heterodimerization and downstream signaling following antibody treatment. Upon EGF activation, phosphorylation level of AKT and ERK were significantly increased compared to control cells (Fig 5A-5C). In MCF7 cells, r40 reduced HER2 protein levels and inhibited p-AKT (S473) (Fig 5A). In BT-474 cells, r40 showed no effect on HER2

protein levels but inhibited p-AKT (S473) and p-ERK (T202/Y204) to an extent comparable to trastuzumab and pertuzumab (Fig 5B). The triple antibody combination exhibited the most potent inhibiton of p-AKT (S473) and HER2 levels (Fig 5B). In SK-BR-3 cells, m66 inhibited p-AKT (S473) more potently than r40 (Fig 5C). Furthermore, the triple combination downregulated HER2 more effectively than the trastuzumab-pertuzumab dual combination (Fig 5C).

Furthermore, we investigated the effect of antibodies on NRG1-induced HER2/HER3 heterodimerization and downstream signaling in the three breast cancer cells. In MCF7 cells, pertuzumab completely blocked NRG1 activation (Fig 5D). Antibody r40 was as effective as trastuzumab in reducing p-AKT (S473) and p-ERK (T202/Y204) levels compared to NRG1 activated control cells (Fig 5D). In both BT-474 and SK-BR-3 cells, r40 and trastuzumab exerted minimal effects on p-AKT (S473), p-ERK (T202/Y204) and HER2 levels (Fig 5E, 5F). However, the triple antibody combination exhibited the most potent inhibitory effect on p-AKT (S473) and HER2 (Fig 5E, 5F). Additionally, the triple combination significantly reduced p-ERK (T202/Y204) levels in BT-474 cells (Fig 5E).

Taken together, r40 suppressed HER2 signaling in both ligand-independent and ligand-dependent contexts and enhanced the efficacy of trastuzumab and pertuzumab.

**Complex assembly and structure determination**

For the purposes of structural study, antibodies initially in the IgG format were converted to the fragment antigen-binding (Fab) format (S1B Fig, S1C Fig). To obtain the trimer comprising HER2, m66 and trastuzumab, size exclusion chromatography (SEC) was conducted to obtain the binary complex of HER2 and the m66 Fab. As indicated by the SEC profiles, HER2 protein fully engaged with m66 Fab to form a binary complex, with an additional peak corresponding to the unbound m66 Fab (S3A Fig). The peak of HER2-m66 binary complex was shifted forward with the addition of the trastuzumab Fab, indicating that HER2, m66 and trastuzumab formed a ternary complex (S3B-S3D Fig).

To obtain the tetrameric complex containing HER2, r40, trastuzumab, and pertuzumab, we first purified the binary complex containing HER2 and r40, followed

by the addition of trastuzumab (S4A Fig, S4B Fig). The detectable shift of the SEC peak was observed with the addition of the pertuzumab Fab, indicating the presence of the larger tetrameric complex (S4C-S4E Fig).

Single-particle cryo-EM reconstruction was used to determine the structures of the two HER2-antibodies complexes (S5 Fig, S6 Fig). The cryo-EM maps of HER2-m66-trastuzumab and HER2-r40-trastuzumab-pertuzumab were refined to overall resolutions of 3.2 Å and 3.1 Å, respectively. The final structural models were built with the HER2-trastuzumab-pertuzumab structure (PDB: 6OGE)[10] as a template and Alphafold2 structure prediction. For the HER2-m66-trastuzumab complex, the three components could be clearly identified in the cryo-EM map, and a final high-resolution model was built and refined (Fig 6A). Trastuzumab bound ECD IV and the epitope of m66 was located in ECD I of HER2. We compared HER2-trastuzumab-pertuzumab (PDB: 6OGE) and HER2-m66-trastuzumab we determined with HER2 superimposed (Fig 6B). The binding site of pertuzumab is located at the dimerization interface of HER2. There would be a steric clash if m66 and pertuzumab were both bound, which explained why m66 and pertuzumab didn’t not bind HER2 simultaneously. Superimposition of the HER2-HER3-NRG1 structure (PDB: 7MN5)[31] and HER2-m66-trastuzumab structure showed that m66 was unable to suppress HER2 homodimerization and heterodimerization (Fig 6B).

In the tetrameric structure of HER2-r40-trastuzumab-pertuzumab, r40 targeted the ECD III/IV of HER2, a site distinct from the dimerization interface of HER2 (Fig 6C). The superimposed structure of HER2-HER3-NRG1 (PDB: 7MN5) and HER2-r40-trastuzumab-pertuzumab structure showed that the epitope of antibody r40 was opposite to the dimerization interfaces of HER2, which indicated r40 did not directly disturb the dimerization of HER2 (Fig 6D). The large interface between r40 and HER2 established the high affinity and inhibitory activity of r40 on HER2.

## Discussion

Antibody drugs have revolutionized cancer treatment by specifically targeting tumor antigens, thereby enhancing therapeutic efficacy and reducing side effects, making them a cornerstone of modern oncology. Two approved anti-HER2 antibodies, trastuzumab and pertuzumab specifically target distinct regions of the HER2 protein: trastuzumab targets the juxtamembrane region of ECD IV, while pertuzumab binds near the core of ECD II[10]. Till now, no antibody therapeutics have been identified that selectively target the ECD I and ECD III of HER2. Nevertheless, ongoing studies are exploring new HER2 epitopes to develop more effective treatment strategies.

To obtain antibodies targeting distinct epitopes from those recognized by trastuzumab and pertuzumab, we performed ELISA and flow cytometry with saturated trastuzumab and pertuzumab. The data show that m66 competes with pertuzumab and r40 recognizes a different epitope from that of trastuzumab and pertuzmab. The Cryo-EM structures further confirm the epitopes of m66 and r40 antibodies.

In the absence of ligands, overexpressed HER2 can form homodimers[25]. EGF and NRG1 play pivotal roles in the activation of HER2 signaling. Upon binding to EGFR, EGF triggers a conformational change that extends EGFR from a bent form to an extended state, thereby revealing its dimerization arm. This arm of HER2 then engages with the binding pocket of EGFR, establishing a stable EGFR/HER2 heterodimer[32]. NRG1, through its EGF-like domain, binds to HER3 and HER4, thereby promoting the heterodimerization of HER2/HER3 and HER2/HER4[31, 33]. These three heterodimers induce activation of the downstream PI3K/AKT and MAPK signaling pathways, thereby drive the growth of cells. In our study, r40 effectively suppresses HER2 signaling in both ligand-independent and ligand-dependent contexts, whereas m66 exerts a weak inhibitory effect.

Trastuzumab has been shown to facilitate the internalization of HER2, directing it into endosomes, and subsequently routing it to lysosomes for degradation[34]. In both ligand-dependent and ligand-independent contexts, we observed that the r40 antibody, like trastuzumab, diminished the expression levels of HER2 protein in MCF7 and SK-BR-3 cells (Fig 4, Fig 5). Conversely, the m66 antibody, similar to pertuzumab, had a much relatively weak influence on HER2 protein levels (Fig 4, Fig 5). We hypothesize that r40 induces the internalization of HER2, whereas m66 does not. One potential

reason is that r40 exhibits higher affinity than m66, and the stable antigen-antibody complex structure triggers the internalization process. Another possible explanation is that the binding of m66 to ECD I does not induce conformational changes, however the epitope targeted by the r40 antibody is situated at the interface between ECD III and IV, and conformational shifts within these domains are more prone to trigger the internalization of HER2.

In summary, we screened a panel of human HER2 antibodies and identified r40 as an ECD III/IV-specific antibody that disrupted PI3K/AKT and MAPK signaling pathways and suppresses breast cancer cell growth. Critically, r40 enhanced the efficacy of the trastzumab-pertuzumab combination. These findings suggest r40 is a promising candidate to augment existing HER2-targeted therapies, potentially improving outcomes for HER2-overexpressing cancers. Future work should include detailed mechanistic analyses and humanization of r40.

## References

1. Citri A, Yarden Y. EGF-ERBB signalling: towards the systems level. Nat Rev Mol Cell Biol. 2006;7(7):505-16. doi: 10.1038/nrm1962. PubMed PMID: 16829981.

2. Weisser NE, Sanches M, Escobar-Cabrera E, O'Toole J, Whalen E, Chan PWY, et al. An anti-HER2 biparatopic antibody that induces unique HER2 clustering and complement-dependent cytotoxicity. Nature communications. 2023;14(1):1394-. doi: 10.1038/s41467-023-37029-3. PubMed PMID: 36914633.

3. Tzahar E, Waterman H, Chen X, Levkowitz G, Karunagaran D, Lavi S, et al. A hierarchical network of interreceptor interactions determines signal transduction by Neu differentiation factor/neuregulin and epidermal growth factor. Mol Cell Biol. 1996;16(10):5276-87. doi: 10.1128/MCB.16.10.5276. PubMed PMID: 8816440; PubMed Central PMCID: PMC231527.

4. Junttila TT, Akita RW, Parsons K, Fields C, Lewis Phillips GD, Friedman LS, et al. Ligand-independent HER2/HER3/PI3K complex is disrupted by trastuzumab and is effectively inhibited by the PI3K inhibitor GDC-0941. Cancer Cell. 2009;15(5):429-40. doi: 10.1016/j.ccr.2009.03.020. PubMed PMID: 19411071.

5. Lewis GD, Figari I, Fendly B, Wong WL, Carter P, Gorman C, et al. Differential responses of human tumor cell lines to anti-p185HER2 monoclonal antibodies. Cancer Immunol Immunother. 1993;37(4):255-63. doi: 10.1007/BF01518520. PubMed PMID: 8102322.

6. Clynes RA, Towers TL, Presta LG, Ravetch JV. Inhibitory Fc receptors modulate in vivo cytotoxicity against tumor targets. Nat Med. 2000;6(4):443-6. doi: 10.1038/74704. PubMed PMID: 10742152.

7. Nahta R, Esteva FJ. Herceptin: mechanisms of action and resistance. Cancer Lett. 2006;232(2):123-38. doi: 10.1016/j.canlet.2005.01.041. PubMed PMID: 16458110.

8. Franklin MC, Carey KD, Vajdos FF, Leahy DJ, de Vos AM, Sliwkowski MX. Insights into ErbB signaling from the structure of the ErbB2-pertuzumab complex. Cancer Cell. 2004;5(4):317-28. doi: 10.1016/s1535-6108(04)00083-2. PubMed PMID: 15093539.

9. Adams CW, Allison DE, Flagella K, Presta L, Clarke J, Dybdal N, et al. Humanization of a recombinant monoclonal antibody to produce a therapeutic HER dimerization inhibitor, pertuzumab. Cancer Immunol Immunother.

2006;55(6):717-27. doi: 10.1007/s00262-005-0058-x. PubMed PMID: 16151804.
10. Hao Y, Yu X, Bai Y, McBride HJ, Huang X. Cryo-EM Structure of HER2-trastuzumab-pertuzumab complex. PLoS One. 2019;14(5):e0216095. doi: 10.1371/journal.pone.0216095. PubMed PMID: 31042744; PubMed Central PMCID: PMC6493747.
11. Scheuer W, Friess T, Burtscher H, Bossenmaier B, Endl J, Hasmann M. Strongly enhanced antitumor activity of trastuzumab and pertuzumab combination treatment on HER2-positive human xenograft tumor models. Cancer Res. 2009;69(24):9330-6. doi: 10.1158/0008-5472.CAN-08-4597. PubMed PMID: 19934333.
12. Nahta R, Hung MC, Esteva FJ. The HER-2-targeting antibodies trastuzumab and pertuzumab synergistically inhibit the survival of breast cancer cells. Cancer Res. 2004;64(7):2343-6. doi: 10.1158/0008-5472.can-03-3856. PubMed PMID: 15059883.
13. von Minckwitz G, Procter M, de Azambuja E, Zardavas D, Benyunes M, Viale G, et al. Adjuvant Pertuzumab and Trastuzumab in Early HER2-Positive Breast Cancer. N Engl J Med. 2017;377(2):122-31. doi: 10.1056/NEJMoa1703643. PubMed PMID: 28581356; PubMed Central PMCID: PMC5538020.
14. Bilancia D, Rosati G, Dinota A, Germano D, Romano R, Manzione L. Lapatinib in breast cancer. Ann Oncol. 2007;18 Suppl 6:vi26-30. doi: 10.1093/annonc/mdm220. PubMed PMID: 17591827.
15. Sonpavde G. Lapatinib plus capecitabine in breast cancer. N Engl J Med. 2007;356(14):1471; author reply -2. doi: 10.1056/NEJMc070182. PubMed PMID: 17409332.
16. Lopez-Tarruella S, Jerez Y, Marquez-Rodas I, Martin M. Neratinib (HKI-272) in the treatment of breast cancer. Future Oncol. 2012;8(6):671-81. doi: 10.2217/fon.12.66. PubMed PMID: 22764764.
17. Verma S, Miles D, Gianni L, Krop IE, Welslau M, Baselga J, et al. Trastuzumab emtansine for HER2-positive advanced breast cancer. N Engl J Med. 2012;367(19):1783-91. doi: 10.1056/NEJMoa1209124. PubMed PMID: 23020162; PubMed Central PMCID: PMC5125250.
18. Cortes J, Hurvitz SA, Im SA, Iwata H, Curigliano G, Kim SB, et al. Trastuzumab deruxtecan versus trastuzumab emtansine in HER2-positive metastatic breast

cancer: long-term survival analysis of the DESTINY-Breast03 trial. Nat Med. 2024;30(8):2208-15. doi: 10.1038/s41591-024-03021-7. PubMed PMID: 38825627; PubMed Central PMCID: PMC11333275.
19. Oh DY, Bang YJ. HER2-targeted therapies - a role beyond breast cancer. Nature Reviews Clinical Oncology. 2020;17(1):33-48. doi: 10.1038/s41571-019-0268-3. PubMed PMID: 31548601.
20. Shitara K, Bang YJ, Iwasa S, Sugimoto N, Ryu MH, Sakai D, et al. Trastuzumab deruxtecan in HER2-positive advanced gastric cancer: exploratory biomarker analysis of the randomized, phase 2 DESTINY-Gastric01 trial. Nat Med. 2024;30(7):1933-42. doi: 10.1038/s41591-024-02992-x. PubMed PMID: 38745009; PubMed Central PMCID: PMC11271396.
21. Okines AF, Cunningham D. Trastuzumab: a novel standard option for patients with HER-2-positive advanced gastric or gastro-oesophageal junction cancer. Therap Adv Gastroenterol. 2012;5(5):301-18. doi: 10.1177/1756283X12450246. PubMed PMID: 22973416; PubMed Central PMCID: PMC3437538.
22. Rexer BN, Arteaga CL. Intrinsic and acquired resistance to HER2-targeted therapies in HER2 gene-amplified breast cancer: mechanisms and clinical implications. Crit Rev Oncog. 2012;17(1):1-16. doi: 10.1615/critrevoncog.v17.i1.20. PubMed PMID: 22471661; PubMed Central PMCID: PMC3394454.
23. Liu X, Song Y, Cheng P, Liang B, Xing D. Targeting HER2 in solid tumors: Unveiling the structure and novel epitopes. Cancer Treat Rev. 2024;130:102826. doi: 10.1016/j.ctrv.2024.102826. PubMed PMID: 39270365.
24. Pedersen MW, Jacobsen HJ, Koefoed K, Dahlman A, Kjaer I, Poulsen TT, et al. Targeting Three Distinct HER2 Domains with a Recombinant Antibody Mixture Overcomes Trastuzumab Resistance. Mol Cancer Ther. 2015;14(3):669-80. doi: 10.1158/1535-7163.MCT-14-0697. PubMed PMID: 25612619.
25. Meng Y, Zheng L, Yang Y, Wang H, Dong J, Wang C, et al. A monoclonal antibody targeting ErbB2 domain III inhibits ErbB2 signaling and suppresses the growth of ErbB2-overexpressing breast tumors. Oncogenesis. 2016;5(3):e211. doi: 10.1038/oncsis.2016.25. PubMed PMID: 26999718; PubMed Central PMCID: PMC4815051.
26. Fu W, Wang Y, Zhang Y, Xiong L, Takeda H, Ding L, et al. Insights into HER2 signaling from step-by-step optimization of anti-HER2 antibodies. MAbs.

2014;6(4):978-90. doi: 10.4161/mabs.28786. PubMed PMID: 24838231; PubMed Central PMCID: PMC4171031.
27. Tamaskovic R, Schwill M, Nagy-Davidescu G, Jost C, Schaefer DC, Verdurmen WP, et al. Intermolecular biparatopic trapping of ErbB2 prevents compensatory activation of PI3K/AKT via RAS-p110 crosstalk. Nat Commun. 2016;7:11672. doi: 10.1038/ncomms11672. PubMed PMID: 27255951; PubMed Central PMCID: PMC4895728.
28. Kast F, Schwill M, Stuber JC, Pfundstein S, Nagy-Davidescu G, Rodriguez JMM, et al. Engineering an anti-HER2 biparatopic antibody with a multimodal mechanism of action. Nat Commun. 2021;12(1):3790. doi: 10.1038/s41467-021-23948-6. PubMed PMID: 34145240; PubMed Central PMCID: PMC8213836.
29. Selitrennik M, Lev S. PYK2 integrates growth factor and cytokine receptors signaling and potentiates breast cancer invasion via a positive feedback loop. Oncotarget. 2015;6(26):22214-26. doi: 10.18632/oncotarget.4257. PubMed PMID: 26084289; PubMed Central PMCID: PMCPMC4673158.
30. Berdiel-Acer M, Maia A, Hristova Z, Borgoni S, Vetter M, Burmester S, et al. Stromal NRG1 in luminal breast cancer defines pro-fibrotic and migratory cancer-associated fibroblasts. Oncogene. 2021;40(15):2651-66. doi: 10.1038/s41388-021-01719-3. PubMed PMID: 33692466; PubMed Central PMCID: PMCPMC8049869.
31. Diwanji D, Trenker R, Thaker TM, Wang F, Agard DA, Verba KA, et al. Structures of the HER2-HER3-NRG1beta complex reveal a dynamic dimer interface. Nature. 2021;600(7888):339-43. doi: 10.1038/s41586-021-04084-z. PubMed PMID: 34759323; PubMed Central PMCID: PMC9298180.
32. Yu X, Wang L, Shen Y, Wang C, Zhang Y, Meng Y, et al. Targeting EGFR/HER2 heterodimerization with a novel anti-HER2 domain II/III antibody. Molecular Immunology. 2017;87:300-7. doi: 10.1016/j.molimm.2017.05.010. PubMed PMID: 28531814.
33. Trenker R, Diwanji D, Bingham T, Verba KA, Jura N. Structural dynamics of the active HER4 and HER2/HER4 complexes is finely tuned by different growth factors and glycosylation. Elife. 2024;12. doi: 10.7554/eLife.92873. PubMed PMID: 38498590; PubMed Central PMCID: PMC10948148.
34. Baselga J, Albanell J. Mechanism of action of anti-HER2 monoclonal

antibodies. Ann Oncol. 2001;12 Suppl 1:S35-41. doi: 10.1093/annonc/12.suppl_1.s35. PubMed PMID: 11521720.
35. Zheng SQ, Palovcak E, Armache JP, Verba KA, Cheng Y, Agard DA. MotionCor2: anisotropic correction of beam-induced motion for improved cryo-electron microscopy. Nat Methods. 2017;14(4):331-2. doi: 10.1038/nmeth.4193. PubMed PMID: 28250466; PubMed Central PMCID: PMC5494038.
36. Zhang K. Gctf: Real-time CTF determination and correction. J Struct Biol. 2016;193(1):1-12. doi: 10.1016/j.jsb.2015.11.003. PubMed PMID: 26592709; PubMed Central PMCID: PMC4711343.
37. Scheres SH. RELION: implementation of a Bayesian approach to cryo-EM structure determination. J Struct Biol. 2012;180(3):519-30. doi: 10.1016/j.jsb.2012.09.006. PubMed PMID: 23000701; PubMed Central PMCID: PMC3690530.
38. Kimanius D, Forsberg BO, Scheres SH, Lindahl E. Accelerated cryo-EM structure determination with parallelisation using GPUs in RELION-2. Elife. 2016;5. doi: 10.7554/eLife.18722. PubMed PMID: 27845625; PubMed Central PMCID: PMC5310839.
39. Punjani A, Rubinstein JL, Fleet DJ, Brubaker MA. cryoSPARC: algorithms for rapid unsupervised cryo-EM structure determination. Nat Methods. 2017;14(3):290-6. doi: 10.1038/nmeth.4169. PubMed PMID: 28165473.
40. Pettersen EF, Goddard TD, Huang CC, Couch GS, Greenblatt DM, Meng EC, et al. UCSF Chimera--a visualization system for exploratory research and analysis. J Comput Chem. 2004;25(13):1605-12. doi: 10.1002/jcc.20084. PubMed PMID: 15264254.
41. Tunyasuvunakool K, Adler J, Wu Z, Green T, Zielinski M, Zidek A, et al. Highly accurate protein structure prediction for the human proteome. Nature. 2021;596(7873):590-6. doi: 10.1038/s41586-021-03828-1. PubMed PMID: 34293799; PubMed Central PMCID: PMC8387240.
42. Emsley P, Cowtan K. Coot: model-building tools for molecular graphics. Acta Crystallogr D Biol Crystallogr. 2004;60(Pt 12 Pt 1):2126-32. doi: 10.1107/S0907444904019158. PubMed PMID: 15572765.

**Acknowledgments**

We thank the Center of Cryo-Electron Microscopy, Core Facility of Shanghai Medical College, Fudan University for the supports on cryo-EM data collection and data analyses.

**Author contributions**

H. Y. and Y. X. supervised the project; C. L. purified the antibodies and conducted the sample preparation for structural and biochemical studies; C. L. and X. L. performed the cell assay; Y. R. and Q. W. conducted cryo-EM data collection, data analyses and model building; D. Z., P. Z., X. D. screened the antibodies; X. H. helped the flow cytometry assay; C. L. and H. Y. performed data analysis, genetated the figures and drafted the manuscript with contributions from Y. X.

**Competing interests**

Authors declare no competing interests.

**Data and materials availability**

The cryo-EM density maps and corresponding atomic coordinates will be made available upon manuscript acceptance.

## Materials and Methods

### Cell culture

BT-474, SK-BR-3 and MCF7 were acquired from the American Type Culture Collection (ATCC). These cells were maintained in DMEM medium (Gibco) supplemented with 10% fetal bovine serum (NEWZERUM) and penicillin/streptomycin (Basalmedia) at the concentration of 100 μg/mL. Expi293 cells were cultured in Union 293 cell feed medium (Union) containing 100 μg/mL penicillin/streptomycin.

### Construction and expression

The DNA sequence encoding human HER2 ECD (residues 23-652) was amplified and inserted into a modified pCAG vector containing a signal peptide and an N-terminal 9× His tag, enabling the production of a secreted HER2 ECD fusion protein. There is a HRV 3C protease recognition site between the His tag and HER2 ECD sequence (S1A Fig). The DNA sequence encoding the 55 amino-acid EGF like domain of human NRG1 (GTSHLVKCAEKEKTFCVNGGECFMVKDLSNPSRYLCKCPNEFTGDRCQNYVMASF) was synthesized and cloned into the same modified pCAG vector (S1A Fig). The fusion proteins were purified from the culture medium of Expi293 cells using Ni-NTA Sepharose Fast Flow (GE Healthcare), and subsequently cleaved with HRV 3C protease to remove the His tag.

### Kinetics and affinity measurement

Binding kinetics of antibodies with HER2 were determined by SPRi using a PlexArray system (Plexera). IgG format antibodies (0.5 mg/mL) were immobilized on an 3D optical crosslinking biosensor, and then a of serial dilutions of HER2 (1-128 nM) in PBS were subjected to a process of 285 s association and 930 s dissociation.

$EC_{50}$ of m66 and r40 was measured with ELISA. In brief, ELISA plates (Corning) were coated with 0.2 ng/μL of HER2 solution overnight at 4 °C. After blocking, a serial of concentrations ($2^{-11}$-$2^{3}$ ng/μL) were added to the plate and incubated for another 1 h at 37 °C. Either HRP-conjugated goat anti-human IgG1 Fc antibody (Millipore, AP113P) or HRP-conjugated goat anti-rabbit IgG (H+L) antibody (Abclonal, AS014) was incubated for 30 min-1 h at 37 °C. After washing with PBST, TMB substrate

(Beyotime, P0206) was added. The reaction was quenched with 1 M HCl, followed by absorbance measurement at 450 nm using a microplate reader (Biotek). $EC_{50}$ estimates were obtained by fitting ELISA binding curves in Graphpad Prism using the log versus response-variable slope model.

**Epitope mapping**

To map epitopes of two mouse antibodies, HER2 ECD protein was diluted to 0.2 μg/mL in 1× PBS and incubated overnight at 4°C. After washing with PBST buffer, non-His-tagged IgG was diluted to 2 μg/mL in blocking buffer（PBST + 3% BSA）and incubated with the immobilized HER2 at 37°C for 1 h. Without discarding the non-His-tagged IgG, 2 μg/mL His-tagged antibodies was immediately added to the wells. Incubation continued at 37°C for 30 min with agitation. After washing, the subsequent binding of His-tagged IgG protein was measured with anti-his-HRP (Genscript, A00612) using ELISA.

To preliminarily characterize the epitopes of rabbit antibodies, Expi 293 cells were suspended in FACS buffer (DPBS + 2% FBS) at $1\times 10^7$ cells/mL with 1:50 Human TruStain FcX™ (Biolegend, 422302), then incubated with or without 10 μg/mL (66.7 nM) of trastuzumab and pertuzumab for 30 min. After washing, the cells were further incubated with 10 μg/mL rabbit monoclonal antibodies for additional 30 min. Cells were stained with AF488-conjugated goat anti-rabbit IgG secondary antibody (Invitrogen, A11008), and subsequently analyzed using a flow cytometer (Thermo Fisher Scientific) and the FlowJo 10.8.1 software.

To determine the saturation concentrations for the binding of trastuzumab and pertuzumab to HER2 receptors on the cell surface of MCF7, $1\times 10^5$ MCF7 cells were incubated with trastuzumab and pertuzumab (0-400 nM) for 30 min. Subsequently, cells were stained with AF647-conjugated goat anti-human secondary antibody (Invitrogen, A21445) and analyzed by flow cytometry.

The two mouse antibodies (m66, m75) and 7 rabbit antibodies (r3, r4, r5, r29, r35, r36, r40) were labeled by AF647 dye functionalized with an NHS Ester. The antibodis were incubated with 20 molar equivalents of AF647 NHS ester (Biotium, 30-3007) for 2 h at room temperature protected from light. The crude conjugation mixture was dialyzed against PBS (pH 7.4) to remove the excess dye. The 1:3 serial dilution of

AF647 labeled antibodies (0-300 nM) were incubated with or without 267 nM (40 μg/mL) trastuzumab and/or pertuzumab pre-saturated MCF7 cells.

**Tumor cell binding by flow cytometric analysis**

BT-474, SK-BR-3 and MCF7 were briefly trypsinized at 37°C, quenched with FBS-containing medium and removed by centrifugation. $1\times 10^5$ cells for each sample were suspended in FACS buffer with Human TruStain FcX™, followed by incubation with 267 nM (40 μg/mL) trastuzumab, pertuzumab, m66, r40 for 30 min at 4 °C. Human IgG isotype (Beyotime, A7001) and rabbit IgG isotype (Beyotime, A7016) were used as the isotype control. Then the cells were incubated for an additional 30 min with either AF647-conjugated goat anti-human secondary antibody (Invitrogen, A21445) or AF488-conjugated goat anti-rabbit secondary antibody (Invitrogen, A11008). Cells were analyzed via flow cytometry after staining, and histograms of anti-HER2 antibodies were compared with their respective isotype controls.

**Cell proliferation assay**

$1\text{-}2\times10^3$ BT-474, SK-BR-3 and MCF7 cells were plated in flatbottomed 96-well cell culture plates overnight and incubated with a 1:3 serial dilution of antibodies (0-100 nM) for 4 d. After treatment, antibody-mediated cell growth inhibition was quantitatively determined using Cell Counting Kit-8 (Selleck, B34302) with a microplate reader Synergy H1 (BioTek).

**Immunoblotting**

For ligand-independent HER2 signaling, MCF7 cells were treated with 5, 10, 25, 50 μg/mL trastuzumab, pertuzumab, m66, r40, the combination of trastuzumab and pertuzumab or the combination of trastuzumab, pertuzumab and r40 (each at 5, 10, 25, 50 μg/mL) for 24 h. BT-474 and SK-BR-3 cells were treated with 10 μg/mL trastuzumab, pertuzumab, m66, r40, the combination of trastuzumab and pertuzumab or the combination of trastuzumab, pertuzumab and r40 (each at 10 μg/mL) for 24 h. For ligand-dependent HER2 signaling, recombinant human NRG1 or EGF (Acro biosystems, EGF-H52H3) were supplemented at the final concentration of 0.5 nM and 5 nM respectively for 10 min after the 10 μg/mL antibodies treatment for 24 h. Following washing with ice-cold 1× PBS, the cells were lysed and the cell lysates were harvested and analyzed using reducing 4-20% gradient SDS-PAGE, followed by

immunoblotting with antibodies targeting GAPDH (Beyotime, AF0006), AKT (Cell Signaling Technology, 9272), phospho-AKT (Ser473) (Cell Signaling Technology, 4058), p44/42 MAPK (Erk1/2) (Cell Signaling Technology, 4695), phospho-p44/42 MAPK (Erk1/2) (Cell Signaling Technology, 4370) or HER2 (Cell Signaling Technology, 4290).

**Fab purification and complex formation**

The heavy chain and light chain expression cassettes of four Fabs (trastuzumab, pertuzumab, m66 and r40) were assembled into a redesigned pCAG vector. One 9× His tag was incorporated into the C-terminal of heavy chain constant domain. Fab fragments were obtained by co-transfection of light chain and heavy chain into suspension Expi 293 cells using PEI (Yeasen, 40816ES08). Following Ni-NTA purification, eluent was dialyzed to remove imidazole.

For HER2-m66-trastuzumab complex, HER2 was pretreated with an excess amount of m66 Fab. The resulting mixture was separated by SEC using a Superdex 200 10/300 column (GE Healthcare) in 20 mM HEPES pH 7.4, 150 mM NaCl. The HER2-m66 binary complex was collected and further incubated with excessive trastuzumab Fab to form the HER2-m66-trastuzumab complex. HER2-r40-trastuzumab-pertuzumab complex was also prepared using a similar procedure.

**Cryo-EM sample preparation and data collection**

For cryo-EM grid preparation, 3 μL of 1.5 mg/mL HER2-m66-trastuzumab and HER2-r40-trastuzumab-pertuzumab were loaded onto the freshly glow-discharged Quantifoil R1.2/1.3 Au grids. Samples were vitrified by a FEI Vitrobot Mark IV with one second blot time at 100% humidity. Subsequently, the grids were applied to a Titan Krios G3i or G4 transmission electron microscope (Thermo Fisher Scientific) with 300 kV accelerating voltage for data collection and characterization. In total, 992 micrographs of HER2-r40-trastuzumab-pertuzumab were automatically recorded via a Titan Krios G3i transmission electron microscope equipped with Gatan K3 direct electron detector for characterization. Micrographs of HER2-r40-trastuzumab-pertuzumab were captured with Serial-EM software in the super-resolution counting mode. The instrument was operated at a nominal magnification of 81,000× in the EFTEM mode, leading to a super-resolution pixel size of 0.532 Å on the image plane. The defocus

values spanned a range of -1.5 μm to -2.5 μm. The micrograph stack was dose-divided into 40 individual frames for dose-per-frame, with each frame receiving approximately 50 e-/ Å$^2$ and 2.3 s exposure time. The other images were captured automatically using a Titan Krios G4 transmission electron microscope equipped with a Falcon 4i direct electron detector. The micrographs were recorded in super-resolution counting mode using the EPU software at a nominal magnification of 130,000× in the NPTEM mode. This configuration achieved a super-resolution pixel size of 0.466 Å on the image plane. With defocus values spanning between -1.5 μm to -2.5 μm, each micrograph stack was dose-fractionated into 1080 frames, receiving approximately 50 e-/ Å$^2$ total electron dose, under 3.51 s exposure time. For further processing, 2,439 and 2,965 micrographs were systematically acquired for the datasets of HER2-m66-trastuzumab and HER2-r40-trastuzumab-pertuzumab, respectively.

**Cryo-EM data processing**

The processing of all cryo-EM datasets involved addressing and correcting drift and beam-induced motion corrections to achieve whole-frame movie alignment via MotionCor2[35]. The micrographs from the Titan Krios G3i were subjected to a binning process with a factor of 2 to achieve a calibrated pixel size of 1.064 Å/pixel, while the Titan Krios G4 datasets were binned similarly and down to 0.932 Å/pixel. Defocus values were determined using Gctf[36] on summed image data, with dose weighting not applied. Subsequent cryo-EM data were processed using RELION v3.1[37, 38] and cryoSPARC v2[39], employing dose-weighted images for analysis.

For HER2-m66-trastuzumab dataset, 2,017,757 particles were automatically picked in RELION with no reference and subsequently subjected to reference-free 2D classification in cryoSPARC. In total, 466,601 particles were selected from good 2D classes for the 3D initial model. In all, 584,245 were selected from high-quality 3D classes and utilized for heterogeneous refinement in cryoSPARC. The refined particles were loaded to RELION again for 2D and 3D classification. A total of 282,644 particles were extracted from well-defined 3D class assignments and subjected to heterogeneous refinement in cryoSPARC, with CTF correction, achieving the HER2-m66-trastuzumab complex at 3.2 Å resolution.

For the dataset of HER2-r40-trastuzumab-pertuzumab acquired using the Titan Krios G3i, an automatic particle selection process yielded 1,490,027 particles, followed

by 2D classification analysis using RELION. From the well-characterized 2D classes, 56,640 particles were chosen to initiate the 3D classification process. Ultimately, 24,229 particles were extracted from high-quality 3D classes and subjected to 3D auto-refinement, achieving a 9.1 Å resolution reconstruction of the HER2-r40-trastuzumab-pertuzumab complex.

For the dataset of HER2-r40-trastuzumab-pertuzumab from Titan Krios G4, leveraging the previously achieved 9.1 Å resolution structure as a template, 2,293,703 particles were automatically selected and underwent 2D classification using RELION. From the well-defined 2D classes, a selection of 1,973,712 particles was made to proceed with the preliminary 3D classification analysis. A total of 450,219 particles were extracted from high-quality 3D classes and subsequently utilized for 3D auto-refinement. After undergoing Bayesian polishing, post-processing and CTF refinement, this process achieved a 3.1 Å resolution reconstruction of the HER2-r40-trastuzumab-pertuzumab complex.

All observed resolutions were established in accordance with the gold-standard Fourier shell correlation (FSC) criterion of 0.143 and the gold-standard FSC curves were validated to address the effects of a soft mask with high-resolution noise substitution. The cryo-EM maps were processed within the cryoSPARC software using module-based tools for B-factor optimization. 3D volume mapping was visualized and evaluated using UCSF Chimera or UCSF ChimeraX[40], with local resolution variations determined via RELION analysis.

**Model building and structure refinement**

With the structure of HER2-trastuzumab-pertuzumab (PDB: 6OGE)[10] serving as the initial structural template, the cryo-EM maps were utilized for model fitting. The HER2-trastuzumab component and the m66 structure from AlphaFold prediction[41] were docked into corresponding cryo-EM maps of HER2-m66-trastuzumab via rigid-body fitting in UCSF Chimera[40]. The HER2-trastuzumab-pertuzumab structure (PDB: 6OGE) and the r40 structure predicted by AlphaFold were incorporated into the cryo-EM maps of HER2-r40-trastuzumab-pertuzumab using UCSF Chimera for rigid-body fitting. Structural models were generated using COOT[42]. Maps and models in the figures were created using PyMOL (https://pymol.org/), UCSF Chimera, or UCSF ChimeraX. Statistics of the map reconstruction and model refinement with detailed

parameters are available in S1 Table.

## Figure Legends

**Fig. 1. Relative epitopes were assessed by ELISA and flow cytometry.** A. Binding of His-tagged IgG to immobilized HER2 was assessed with anti-His-HRP via ELISA under conditions with or without saturating concentrations of non-His-tagged IgG. B. Saturating concentrations of trastuzumab and pertuzumab for binding to HER2 on live MCF7 cells were determined. MCF7 cells were incubated with trastuzumab and pertuzumab (0-400 nM) for 30 min, then stained with AF647-conjugated goat anti-human secondary antibody for flow cytometry analysis. C. MCF7 cells were pre-incubated with supersaturating concentration (267 nM) of trastuzumab and pertuzumab, and the binding of 1-300 nM trastuzumab-AF647 and pertuzumab-AF647 was evaluated by flow cytometry. D. Binding of m66-AF647 and m75-AF647 to MCF7 that were pre-incubated with supersaturating concentration (267 nM) of trastuzumab or pertuzumab was evaluated by flow cytometry. E. Binding of r35-AF647 and r40-AF647 to MCF7 that were pre-incubated with supersaturating concentration (267 nM) of trastuzumab or pertuzumab was evaluated by flow cytometry. T, trastuzumab; P, pertuzumab. Error bars, SD. *, $P < 0.05$; **, $P < 0.01$; ***, $P < 0.001$; ****, $P < 0.0001$, T-test.

**Fig. 2. HER2-expressing tumor cell proliferation inhibition and $K_D$ measurement of m66 and r40.** A. BT-474 cells were treated with a 1:3 serial dilution of antibodies (0-100 nM) for 4 d. Cell viability was determined by CCK8 assay. B. The inhibitory effects on cell proliferation induced by antibodies were conducted in SK-BR-3 and MCF7 cells with a 1:3 serial dilution of antibodies (0-100 nM) treatment for 4 d. Data are mean ± SEM from n = 5 (BT-474 and SK-BR-3) and n = 6 (SK-BR-3) independent experiments. C. Dissociation constant measurement of trastuzumab, pertuzumab, m66 and r40 against HER2 by SPRi assay. Trastuzumab, pertuzumab, m66 and r40 at the concentration of 0.5 mg/mL were immobilized on an 3D optical crosslinking biosensor (Plexera), and then a of serial dilutions of HER2 (1-128 nM) in PBS were subjected to a process of 285 s association and 930 s dissociation by PlexArray® HT system (Plexera).

**Fig. 3. Binding profiles of trastuzumab, pertuzumab, m66 and r40 to tumor cell lines expressing HER2.** BT-474 (A), SK-BR-3 (B) and MCF7 (C) were incubated with 267 nM (40 μg/mL) of the antibodies, followed by staining with fluorescence-labeled secondary antibodies. The samples were analyzed via flow cytometry.

**Fig. 4. Antibody r40 inhibits ligand-independent HER2 signaling in BT-474 and SK-BR-3.** A. Immunoblots assessing HER2 signaling upon treatment with 5, 10, 25, 50 μg/mL (33.3, 66.7, 166.7, 333.3 nM) antibodies for 24 h in MCF7. Cell lysates were immunoblotted to detect p-AKT (Ser473), total AKT, p-ERK (T202/Y204), total ERK, HER2, GAPDH. Immunoblots assessing HER2 signaling upon treatment with 10 μg/mL (66.7 nM) antibodies for 24 h in BT-474 (B) and SK-BR-3 (C). For the antibody combination, the final concentration of each antibody is 5, 10, 25, 50 μg/mL for MCF7 and 10 μg/mL for BT-474 and SK-BR-3.

**Fig. 5. Antibody r40 inhibits ligand-dependent signaling.** Immunoblots assessing HER2 downstream signaling in MCF7 (A), BT-474 (B) and SK-BR-3 (C) upon incubation with 10 μg/mL (66.7 nM) antibodies for 24 h under 5 nM EGF stimulation for 10 min. Immunoblots assessing HER2 downstream signaling in MCF7 (D), BT-474 (E) and SK-BR-3 (F) cells with 10 μg/mL (66.7 nM) antibodies treatment for 24 h under 0.5 nM NRG1 stimulation for 10 min.

**Fig. 6. Cryo-EM structures of HER2-m66-trastuzumab and HER2-r40-trastuzumab-pertuzumab are determined.** A. Cryo-EM map depicting the the complex composed of three components: HER2 (sky blue), m66 Fab (orange), and trastuzumab Fab (pink), and the final model (grey surface) incorporating the three components into the map. B. Cryo-EM map illustrated the tetrameric complex consisting of four components: HER2 (sky blue), r40 Fab (green), trastuzumab Fab (pink) and pertuzumab Fab (yellow), and the final construction (grey surface) with the four proteins fitted into the map. C. Superposition of HER2-trastuzumab-pertuzumab structure (PDB: 6OGE) and HER2-m66-trastuzumab structure. Superposition of HER2-HER3-NRG1 structure (PDB: 7MN5) and HER2-m66-trastuzumab structure. Overall structure of HER2-r40-trastuzumab-pertuzumab. Superposition of HER2-HER3-NRG1 structure (PDB: 7MN5) and HER2-r40-trastuzumab-pertuzumab complex.

Fig 1

Click here to access/download;Figure;Fig 1.tif

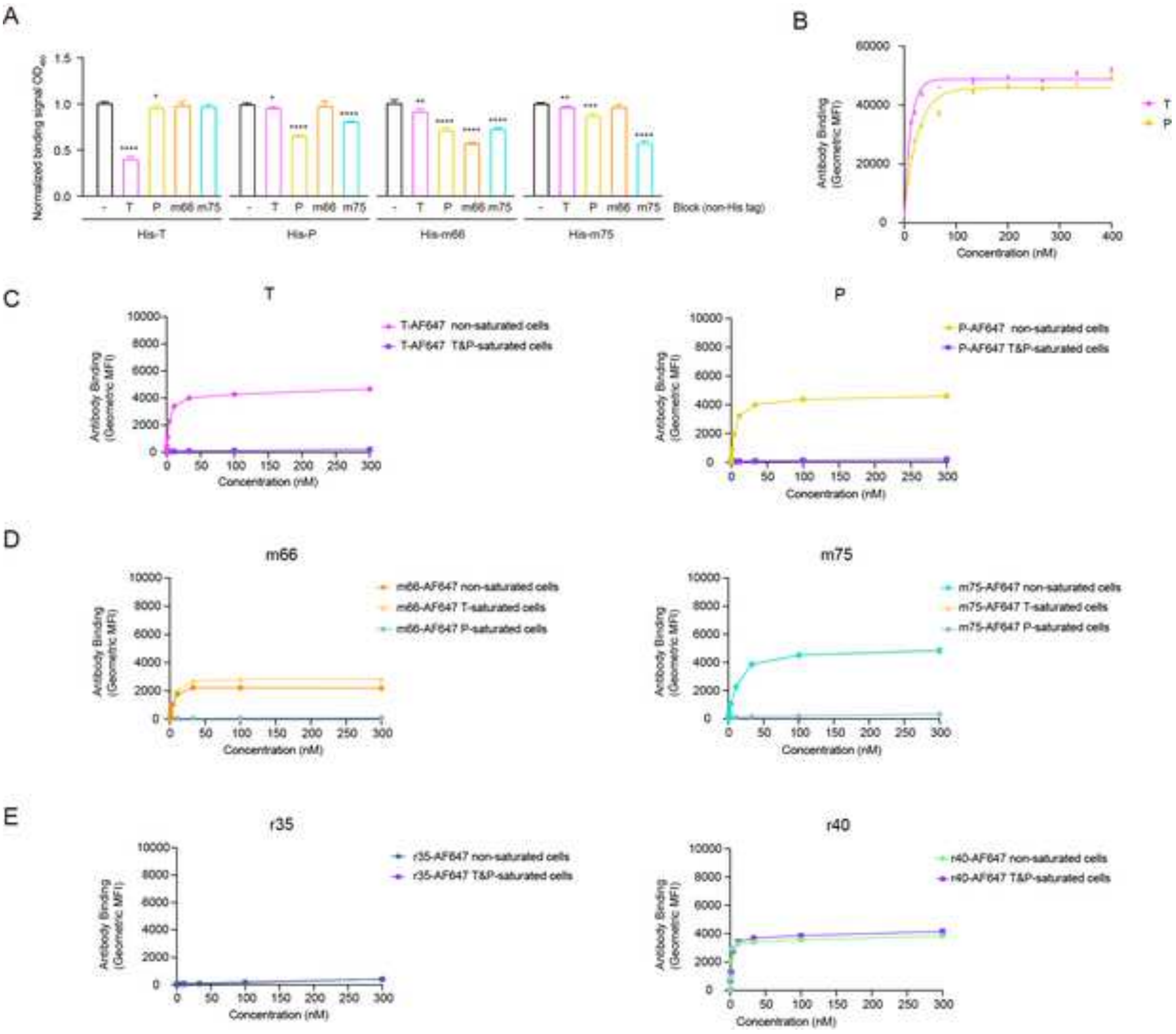

Fig 2

Click here to access/download;Figure;Fig 2.tif

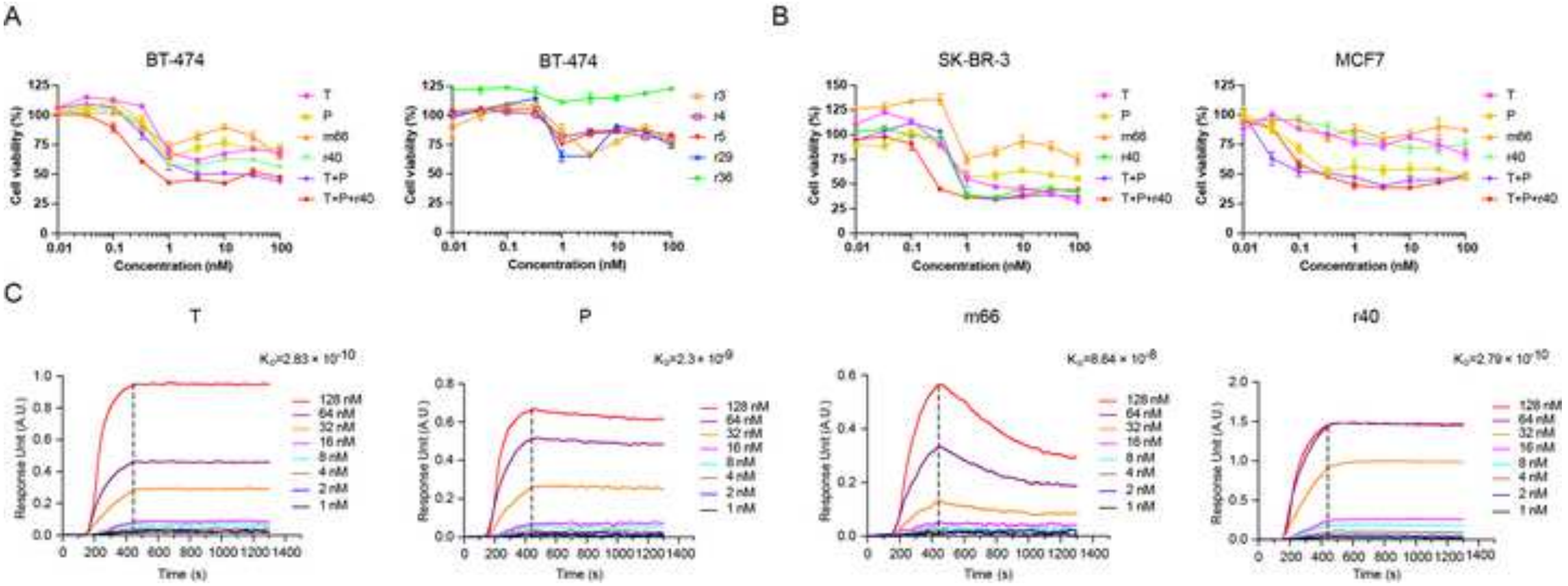

Fig 3

Click here to access/download;Figure;Fig 3.tif

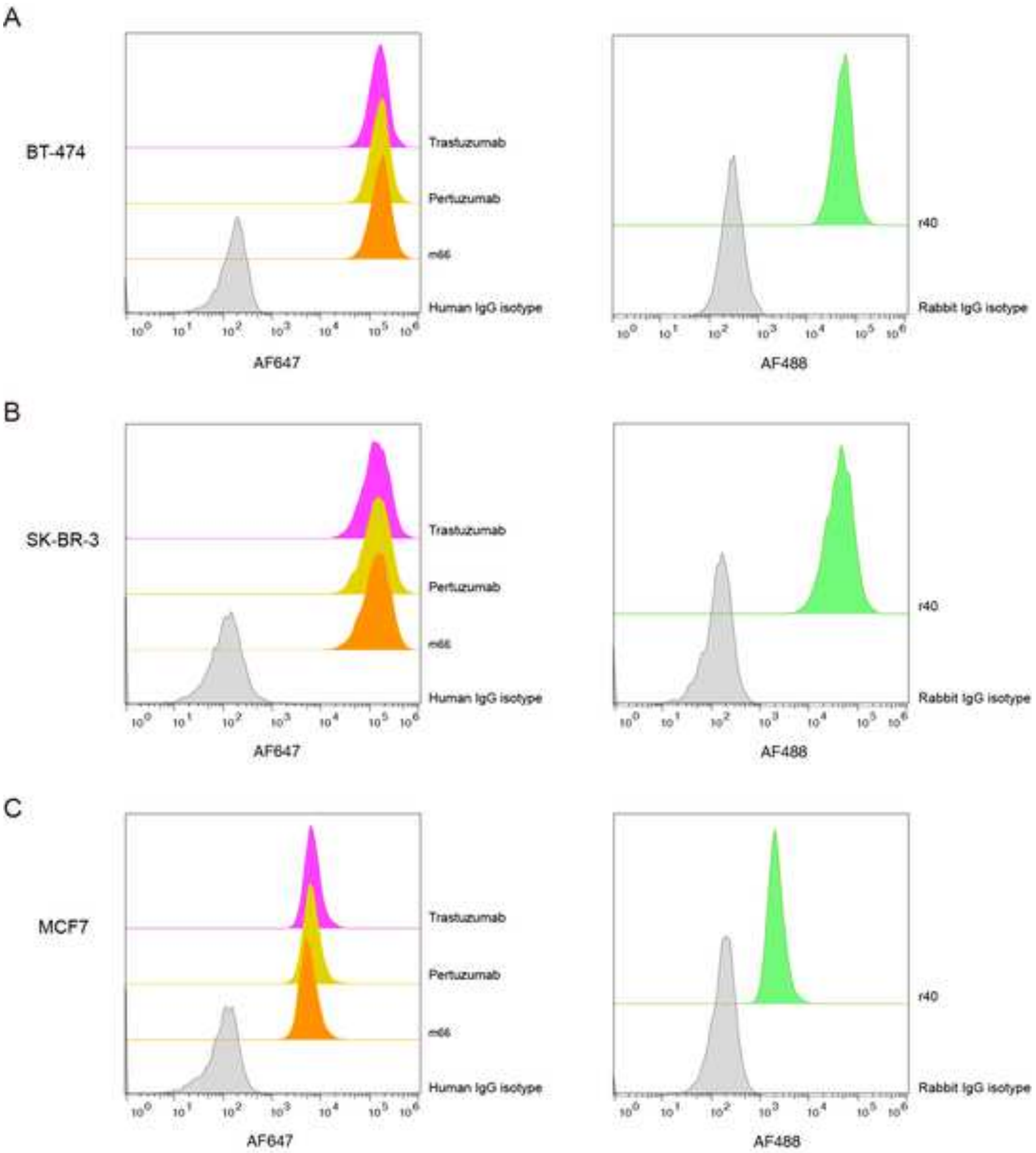

Fig 4

Click here to access/download;Figure;Fig 4.tif

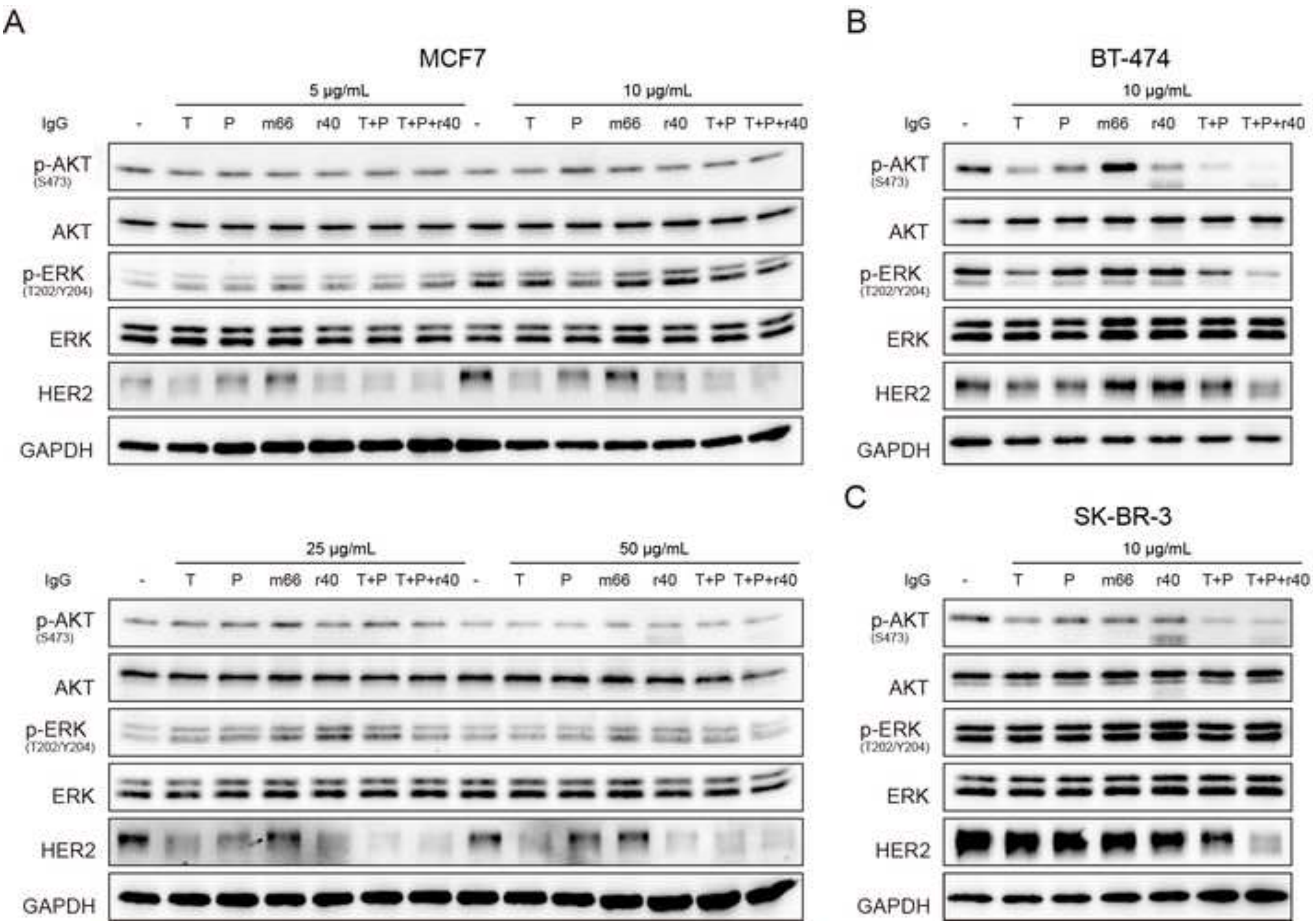

Fig 5

Click here to access/download;Figure;Fig 5.tif

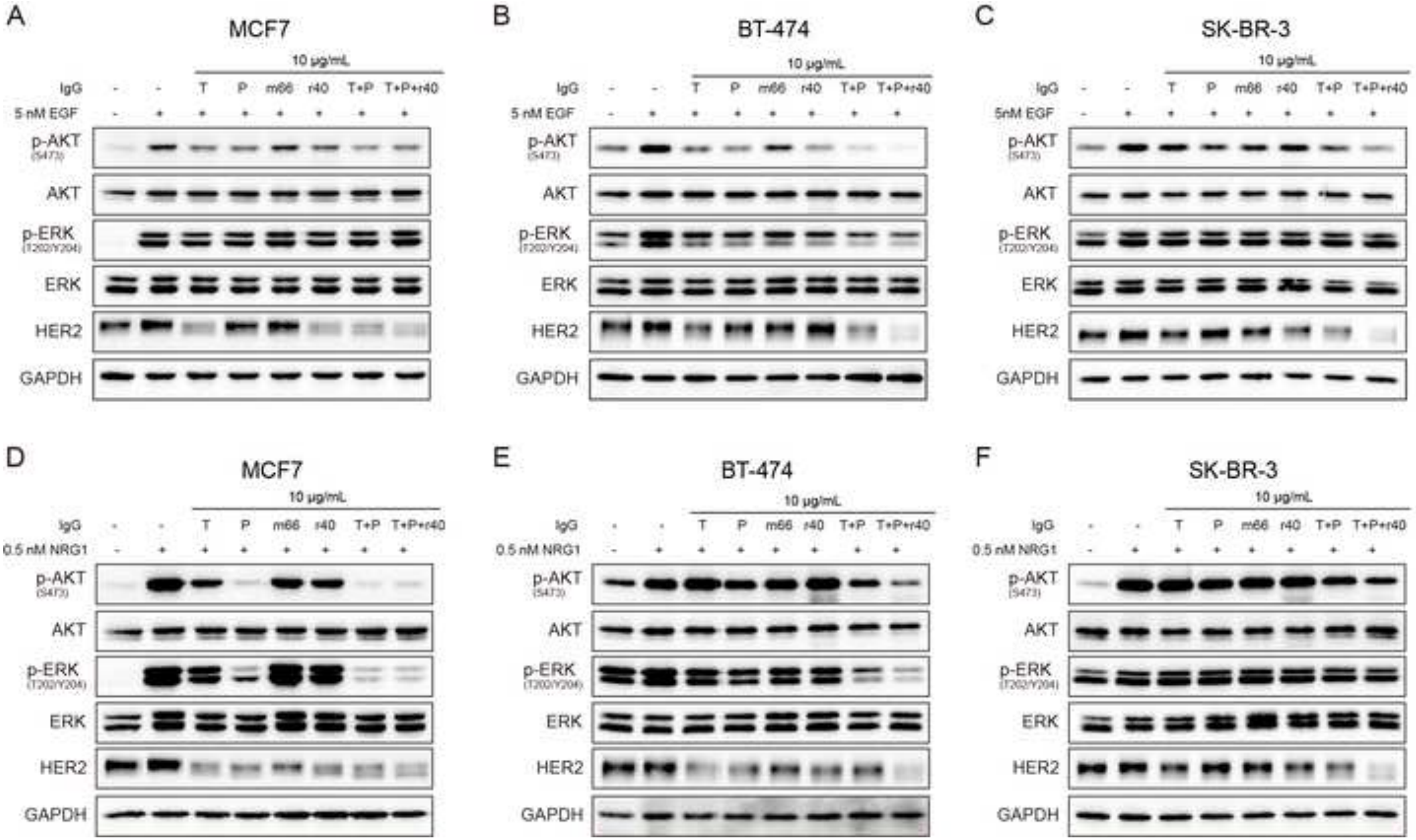

Fig 6

Click here to access/download;Figure;Fig 6.tif

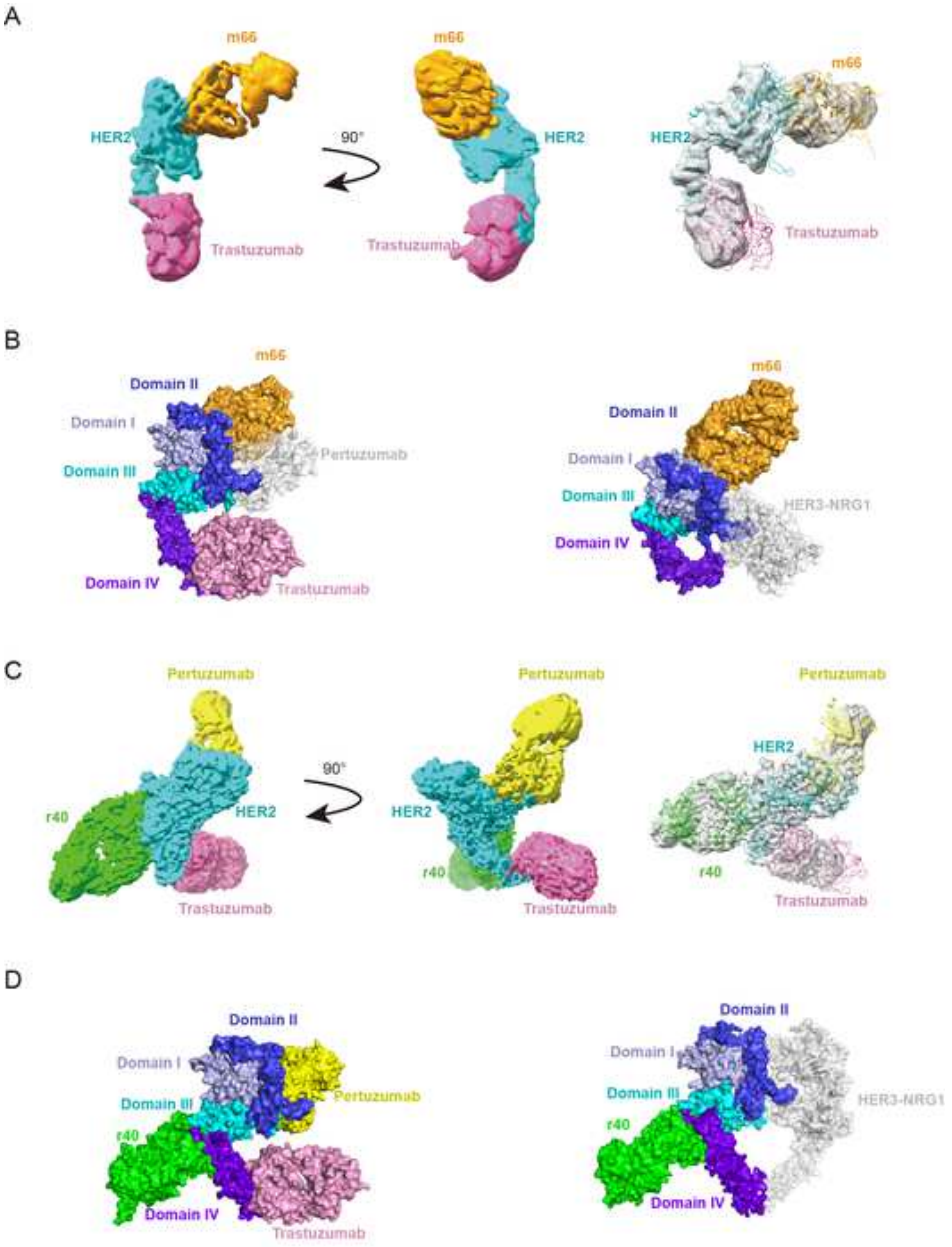

S1 Fig

Click here to access/download;Figure;S1 Fig.tif

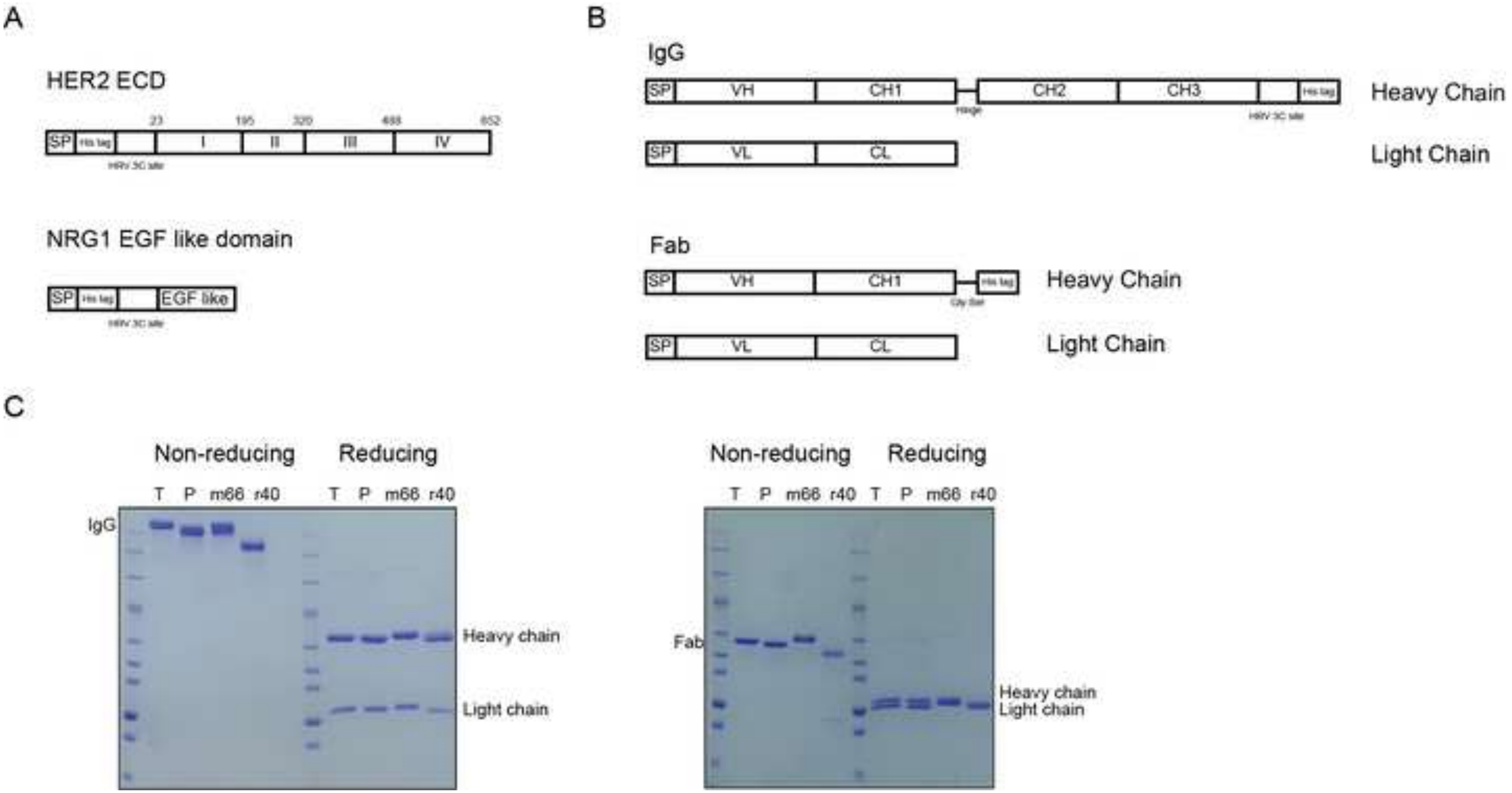

S2 Fig

Click here to access/download;Figure;S2 Fig.tif

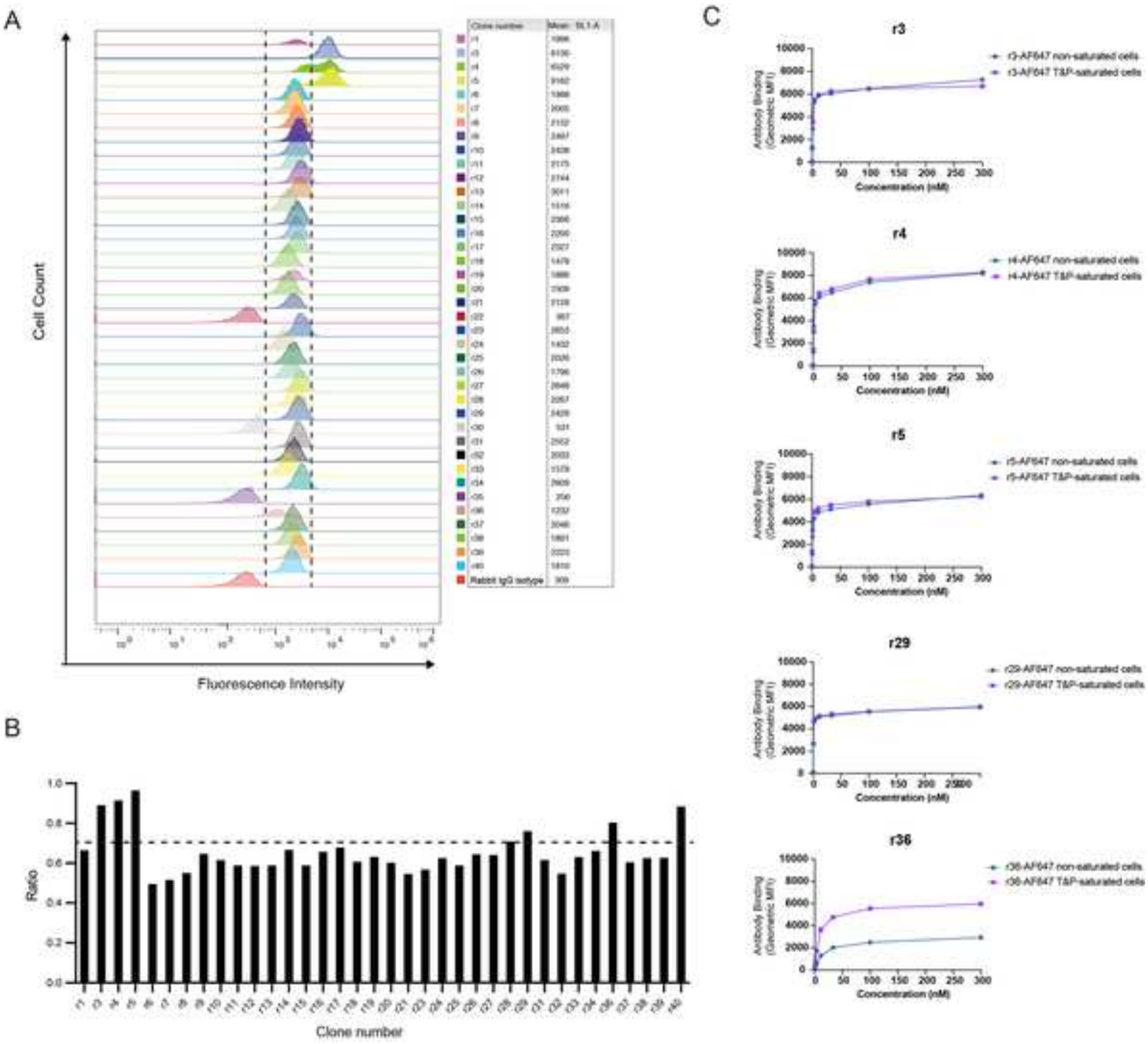

S3 Fig
Click here to access/download;Figure;S3 Fig.tif

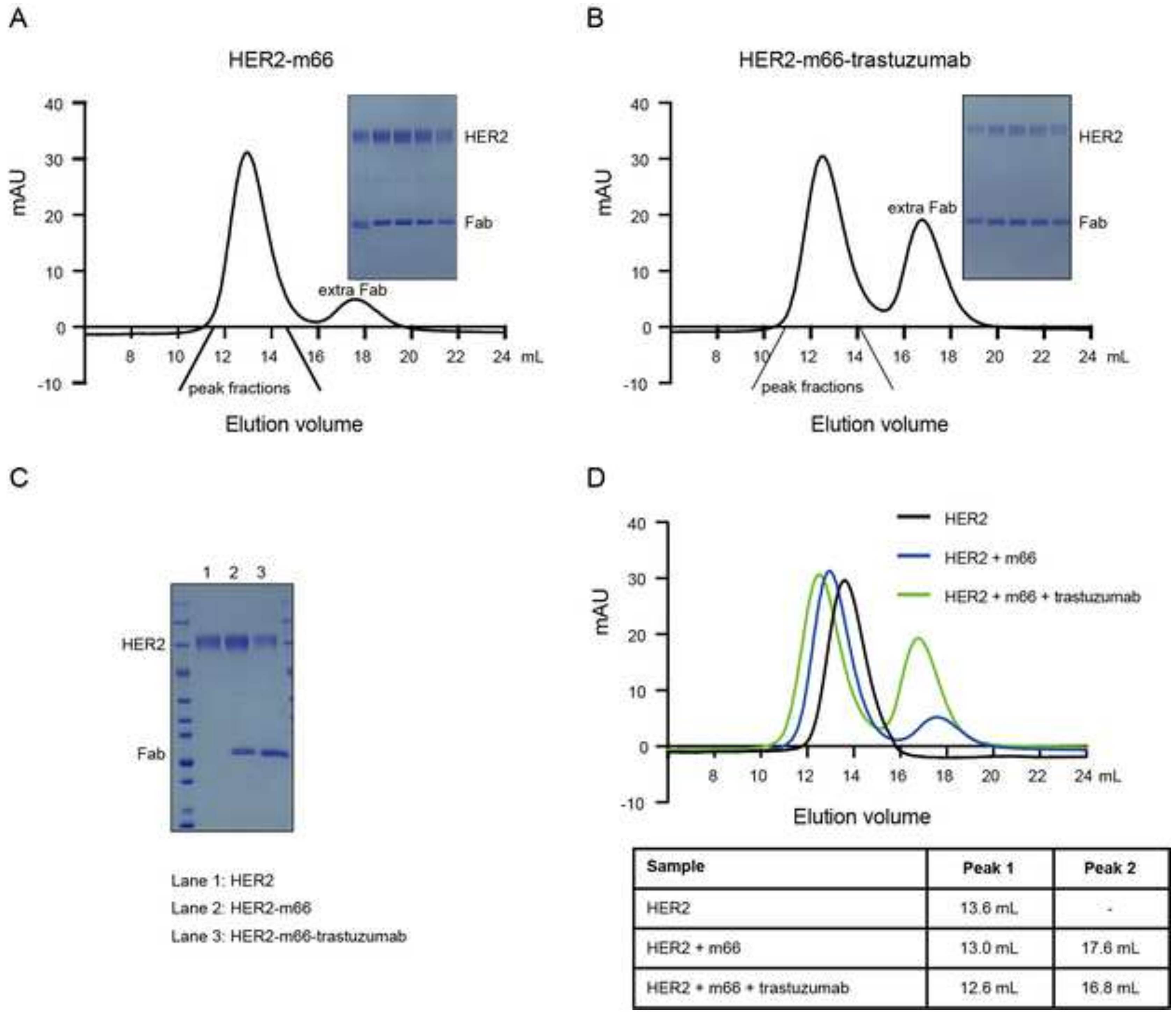


| Sample | Peak 1 | Peak 2 |
|---|---|---|
| HER2 | 13.6 mL | - |
| HER2 + m66 | 13.0 mL | 17.6 mL |
| HER2 + m66 + trastuzumab | 12.6 mL | 16.8 mL |

S4 Fig

Click here to access/download;Figure;S4 Fig.tif

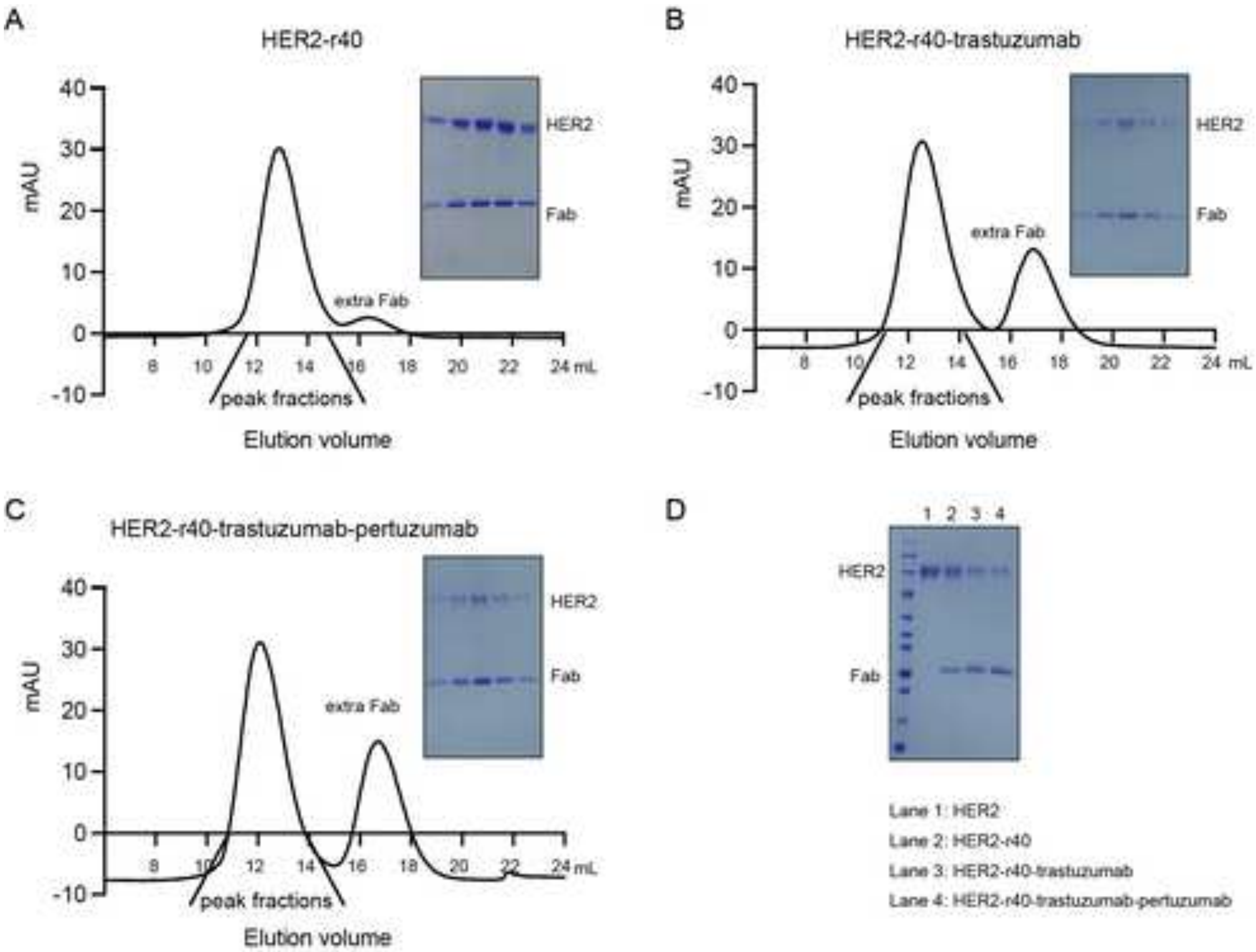


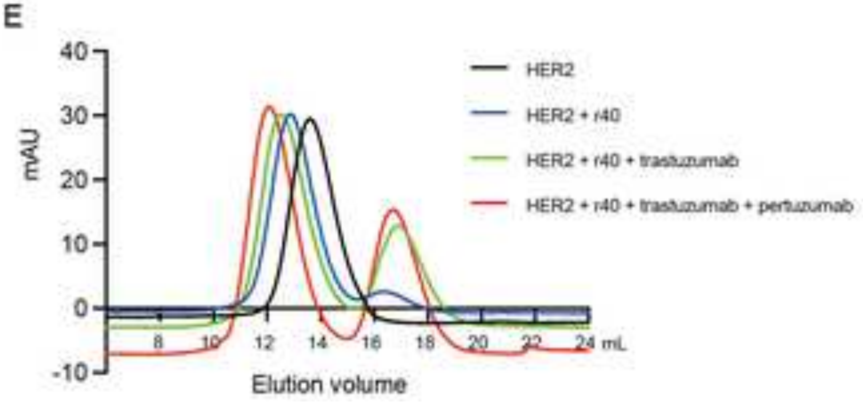


| Sample | Peak 1 | Peak 2 |
|---|---|---|
| HER2 | 13.6 mL | - |
| HER2 + r40 | 12.9 mL | 16.3 mL |
| HER2 + r40 + trastuzumab | 12.5 mL | 16.8 mL |
| HER2 + r40 + trastuzumab + pertuzumab | 12.1 mL | 16.6 mL |

S5 Fig

Click here to access/download;Figure;S5 Fig.tif

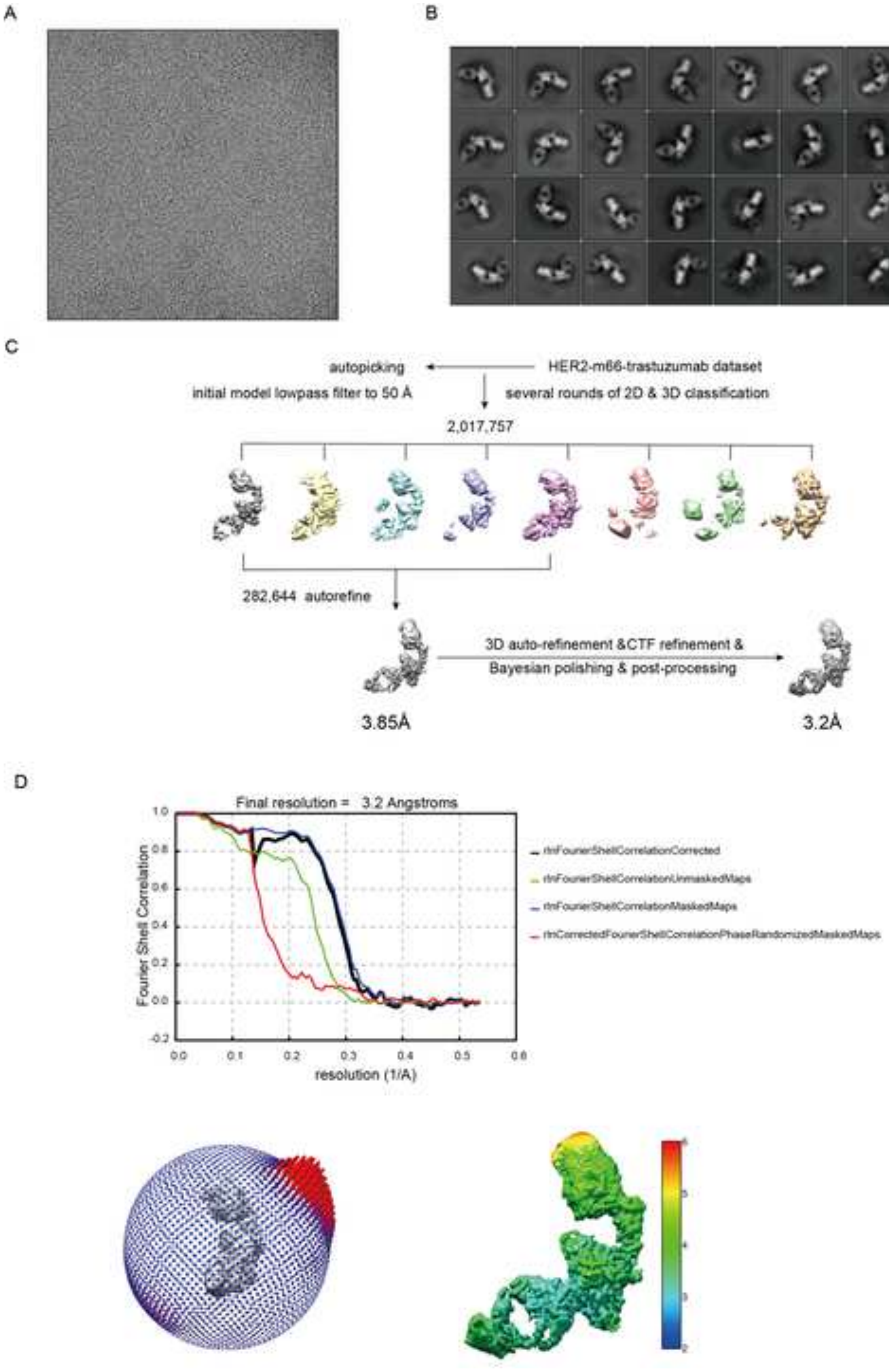

S6 Fig

Click here to access/download;Figure;S6 Fig.tif

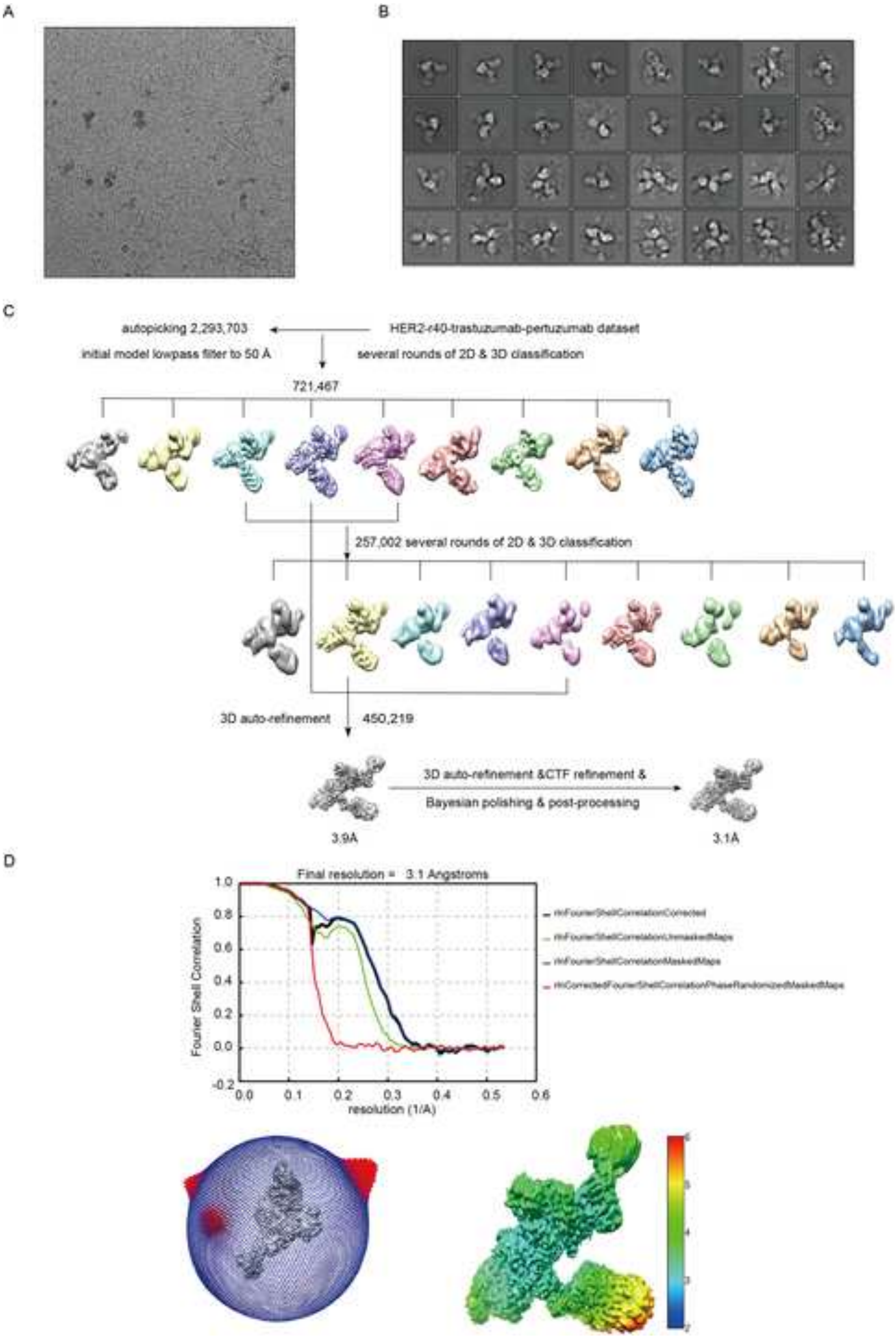

Figure 4 raw images

Click here to access/download

**Supporting Information**

Figure 4 raw images.pdf

Figure 5 raw images

Click here to access/download
**Supporting Information**
Figure 5 raw images.pdf

Raw data

Click here to access/download
**Supporting Information**
Raw data.xlsx

Supplement

Click here to access/download
**Supporting Information**
Supplement.docx

# Inhibition of HER2 signaling and breast cancer cell growth with a novel antibody targeting HER2 ECD III/IV

Chunchun Liu [1, ¶, *], Yulei Ren [1, ¶], Xuan Luo [3, 4, ¶], Peiyun Zhou [1], Xiangjuan Du [1], Qianmin Wang [1], Xue Han [1], Yanhui Xu [1, 2], Ping Wang [6], Dan Zhao [1, 5, *], Huirong Yang [1, 2, *]

[1] Fudan University Shanghai Cancer Center, Institutes of Biomedical Sciences, Shanghai Key Laboratory of Radiation Oncology, and Shanghai Key Laboratory of Medical Epigenetics, Shanghai Medical College of Fudan University, Shanghai, China.

[2] The International Co-laboratory of Medical Epigenetics and Metabolism, Ministry of Science and Technology, China, Department of Systems Biology for Medicine, School of Basic Medical Sciences, Shanghai Medical College of Fudan University, Shanghai, China.

[3] Key Laboratory of Breast Cancer in Shanghai, Department of Breast Surgery, Fudan University Shanghai Cancer Center, Shanghai Medical College, Fudan University, Shanghai, China

[4] Department of Oncology, Shanghai Medical College, Fudan University, Shanghai, China

[5] School of Life Sciences, Greater Bay Area Institute of Precision Medicine (Guangzhou), Fudan University, Guangzhou, China

[6] Molinsights Biotechnologies, Guangdong Medical Valley Life Science Park, Guangzhou, China.

* Corresponding author

E-mail: liuchun880616@yeah.net, danzhao5278@foxmail.com, yanghr@fudan.edu.cn

¶These authors contributed equally to this work.

## Abstract

HER2 (Human epidermal growth factor receptor 2), Human epidermal growth factor receptor 2 (HER2), a ligand-independent tyrosine kinase receptor of the EGFR family, functions as a key oncogenic factor in breast, gastric and several other solid malignancies. Despite the huge survival benefits from anti-HER2 treatment, particularly in HER2 overexpression breast cancer and gastric cancer, the emergence of treatment resistance and cancer recurrence persists as a significant challenge. Thus, the creation of innovative antibodies exhibiting complementary effects to the current anti-HER2 therapies could offer clinical benefits. Here, we describe a two novel rabbit HER2-monoclonal antibody targeting monoclonal antibodies HER2, termed m66 (mouse-derived) and r40 (rabbit-derived), that which demonstrates inhibitory activity on the proliferation ofinhibit breast cancer cellscell proliferation *in vitro*. Antibody r40 exhibits a more potent effect than m66 in suppresseings the PI3K/AKT and MAPK signaling pathways via decreasing the level of HER2 protein. Compared with the combination of trastuzumab and pertuzumab, the addition of r40 significantly enhances the inhibitory effect. –Furthermore, we report the cryo-EM structure determination of HER2-m66-trastuzumab ternary complex and HER2-r40-trastuzumab-pertuzumab-r40 tetrameric complex to overall 3.2 Å –and 3.1 Å resolution, respectively. The structure data indicates that the epitope of m66 overlaps with that of pertuzumab, whereas the epitope of r40 is mappedlies within the HRE2 ECD III/IV region, unlike, distinct from the epitopes of trastuzumab and pertuzumab. The findings of this study highlight that r40 may be considered asis a promising candidate candicate for combinationthe therapy of breast cancer with– trastuzumab and pertuzumab in breast cancer treatmentHER2 overexpression.

Formatted: Font: Italic

## Introduction

The ~~EGFR (~~human epidermal growth factor receptor~~)~~ (EGFR) family comprises four members: EGFR (ERBB1, HER1), HER2 (ERBB2), HER3 (ERBB3) and HER4 (ERBB4). All ~~these four~~ of these HER proteins are ~~considered as~~ oncogenic drivers, with EGFR and HER2 highly associated with tumorigenesis. HER2, characterized as a single-pass type I transmembrane protein, consists ~~is consisted~~ of four extracellular domains (ECD I-IV), a transmembrane domain and an intracellular tyrosine kinase domain from N-to-C terminus. Unlike other EGFR family members, HER2 has the constitutive activity~~,~~ ~~which~~ that can initiate signaling pathways without a bound activating ligand[1, 2]. Overexpression of HER2 results in ligand-independent homodimerization and ligand-dependent heterodimerization of EGFR/HER2, HER2/HER3, HER2/HER4, causing HER2 phosphorylation, specifically activating the PI3K/AKT and MAPK signaling pathways, ~~and~~ thus triggering cell division and growth[1, 3].

Current clinical practice guidelines incorporate various anti-HER2 biologics, ranging from monoclonal antibodies to advanced antibody-drug conjugates (ADC) platforms, for managing cancers with amplified HER2 expression. Trastuzumab (Herceptin®) was approved for the therapeutic treatment of breast cancer and metastatic gastric cancer in patients with HER2 overexpression in 1998 and 2010, respectively. Trastuzumab binds to ECD IV of HER2, ~~which~~ and triggers the internalization and degradation of HER2~~, causing~~. This results in the inhibition of downstream signaling pathways and the elimination of cytotoxic effector T cells ~~via ADCC (~~antibody-dependent cell-mediated cytotoxicity~~)~~ (ADCC) mechanisms[4-6]. Combined with chemotherapy, trastuzumab has demonstrated significant therapeutic benefits in the treatment of HER2-driven breast malignancies. However, therapeutic resistance and disease relapse inevitably occurs after trastuzumab treatment[7]. Pertuzumab (Perjeta®) is the second approved anti-HER2 monoclonal antibody ~~mAb~~ drug that binds to ECD II of HER2[8, 9]. The binding epitopes of trastuzumab and pertuzumab are both situated at the dimerization interface of HER2[10]. Combinational use of trastuzumab and pertuzumab has been evaluated to exhibit enhanced antitumor effect for HER2 overexpressing breast cancer[11-13].

Other anti-HER2 agents, including small molecular drugs (lapatinib, neratinib),

and ADC-based drugs (trastuzumab emtansine, trastuzumab deruxtecan) have been approved for HER2-overexpression breast cancers[14-18]. Trastuzumab and trastuzumab deruxtecan (T-Dxd) are also approved for the therapy of HER2-positive gastric cancers[19-21]. Moreover, metastatic HER2-driven malignancies maglignancies inevitably develop refractoriness to the current approved HER2 therapies, leading to disease progression over time[22].

The observed synergistic anticancer effect of trastuzumab and pertuzumab targeting topographically distinct HER2 epitopes has catalyzed intensive exploration of novel antibodies recognizing new epitopes[23]. The combination of three anti-HER2 antibodies with non-overlapping epitopes showed higher antitumor efficacy than trastuzumab and pertuzumab combination[24]. Also, the combination of a monoclonal antibody targeting HER2 ECD III with trastuzumab and pertuzumab provided blockade of HER2 heterodimerization and cell signaling[25]. Although neither ECD I nor III is directly involved in HER2 dimerization, ECD I is critical for forming a binding pocket for the dimerization arm of other EGFR family members. ECD III, situated in the center of the whole extracellular domain, serves as a pivotal connector between ECD II and ECD IV, and also establishes contact contacts with ECD I. Antibodies targeting ECD I and III can also possess the potential to modulate the conformational dynamics of the HER2 receptor, which in turn could impede the activation cascade of HER2 signaling[23].

In this study, we report two novel HER2 targeted antibodies, m66 (mouse-derived) and r40 (rabbit-derived). aThe novel HER2 ECD III/IV specific monoclonal rabbit antibody r40 r40 by cryo-electron microscopy (cryo-EM) study, which can suppresses the growth of breast cancer cells cell growth via inhibition ofby inhibiting the PI3K/AKT and MAPK signaling pathways. FurthermoreFurhermore, we demonstrate that r40 enhances the efficacy of trastuzumab-pertuzumab combination against breast cancer cell proliferation and HER2 signaling inhibitionblocks the HER2 signaling through decreasing the level of HER2 protein.

# Results

## Discovery of antibodies targeting HER2 ECD

HER2 ECD specific antibodies were obtained by single cell VDJ sequencing (scVDJ-seq) from the spleens of mouse mice and rabbit rabbits immunized with HER2 ECD proteins (S1A Fig). The antibodies selected from B cells were synthesized and expressed *in vitro* and assessed for antigen binding affinity by ELISA and pull-down assays (data not shown). Finally, we acquired got two mouse antibodies (termed m66, m75) and thirty-eight rabbit antibodies among r1-r40 specifically targeting HER2 ECD.

Formatted: Font: Italic

## Antibody m66 competes with pertuzumab for binding to HER2

To characterize the epitopes of the two mouse antibodies, we purified trastuzumab, pertuzumab, m66, and m75 in two human IgG1 formats, both His-tagged and non-His-tagged forms (S1B Fig). We prepared these two formats by performing the purification procedure without or with HRV 3C protease cleavage. Blocking ELISA was performed to assess the binding epitopes by incubating immobilized HER2 with supersaturating non-His-tagged IgGs followed by detecting the binding of His-tagged IgGs. As expected, each non-His-tagged IgG significantly blocked the binding of its own His-tagged IgG (Fig 1A). In addition, supersaturation of m66 and m75 did not influence the binding of trastuzumab, indicating that m66 and m75 target epitopes distinct from that of trastuzumab. The binding of Antibody antibody m66 binding was blocked by pertuzumab but not vice versa, indicating that the binding affinity of m66 is was weaker than that of pertuzumab. While Antibody antibody m75 blocked pertuzumab and pertuzumab blocked m75 (Fig 1A). These results indicated that m66 and m75 bind bound to distinct epitopes from that of trastuzumab, while they likely bind to epitopes overlapping with pertuzumab (Fig 1A).

To map antibody epitope by flow cytometry, we selected the low-HER2-expressing breast cancer cell line MCF7. This model allowed us to determine whether the identified mouse and rabbit antibodies retained binding to HER2 in the presence of saturating concentrations of trastuzumab and pertuzumab. To determine the saturation concentrations of trastuzumab and pertuzumab in MCF7 cells, we incubated the cells with 0–400 nM of each antibody individually. After incubation, cells were stained with

Formatted: Indent: First line: 2 ch

an AF647-conjugated goat anti-human secondary antibody (Invitrogen, A21445) and analyzed by flow cytometry. The saturation concentration for both antibodies was determined to be about 100 nM (Fig 1B).

Subsequently, trastuzumab, pertuzumab, m66 and m75 were conjugated with NHS-AF647 dye. As expected, binding of AF647-conjugated trastuzumab and pertuzumab was precluded in MCF7 cells saturated with unlabeled trastuzumab and pertuzumab (Fig 1C). Binding of both m66-AF647 and m75-AF647 was blocked exclusively by pertuzumab saturation, with minimal inhibition from trastuzumab saturation (Fig 1D). This also indicated that m66 and m75 bound to epitopes overlapping with pertuzumab, which was consistent with ELISA results (Fig 1A). Enhanced binding affinity of m66 to trastuzumab-saturated MCF7 cells suggested that trastuzumab binding altered HER2 conformation, thus increasing m66 affinity (Fig 1D). As a resultWhile in the purification of antibodies, we found that m75 was unstable and prone to precipitation,, antibody m66 antibody was selected for further investigation.

**Antibody r40 binds to a different eptiode epitope from that of trastuzumab and pertuzumab**

To characterize epitopes of rabbit antibodiesy epitopes through competitive binding, we first blockedpre-incubated Expi 293 cellsHER2 with supersaturating 10 μg/mL (66.7 nM) trastuzumab and pertuzumab,. then Then the cells were incubated with incubated cells with rabbit antibodies and analyzed by . Fflow cytometry assay was conducted to assess simultaneous binding capacity.determine whether rabbit antibodies could bind simultaneously. Primarily, we tested the binding ability of thirty-eight39 rabbit antibodies in Expi 293 cells, among which r22, r30, r35 couldn't could not recognize cell surface HER2 (S2A FigFig 1B). When When cells were pre-incubated with trastuzumab and pertuzumab, the mean fluorescence intensity (MFI) of the rabbit monoclonal antibody remained comparable to control cells incubated with the rabbit antibody alone. This indicated that the rabbit antibody bound HER2 without competing for the epitopes of trastuzumab and pertuzumab. saturated with trastuzumab and pertuzumab, the mean fluorescence intensity of a rabbit antibody is nearly identical

~~to that of the control cells solely incubated with the rabbit mAb, which suggests that the rabbit antibody can bind to HER2 with trastuzumab and pertuzumab simultaneously. Thus, we~~ We therefore determined ~~suspected~~ that the six rabbit antibodies (r3, r4, r5, r29, r36 and r40) ~~might~~ recognized epitopes distinct from ~~that~~ those of trastuzumab and pertuzumab (S2B Fig~~Fig 1C, 1D~~).

We further comfirmed the distinct epitope binding of rabbit monoclonal antibodies (r3, r4, r5, r29, r35, r36, r40) relative to trastuzumab and pertuzumab in low-HER2-expressing MCF7 cells (Fig 1E, S2C Fig). Saturation of HER2 in MCF7 cells with trastuzumab and pertuzumab did not inhibit subsequent binding of r40, comfirming its epitope was distinct from those targeted by these two therapeutic antibodies (Fig 1E). Similarly, binding of r3, r4, r5 and r29 remained unaffected, consistent with their epitope divergence from those of trastuzumab and pertuzumab (S2C Fig). In contrast, r36 exhibited increased MFI on trastuzumab-pertuzumab-saturated cells, indicating the binding of the two therapeutic antibodies altered HER2 conformation to enhance r36 affinity (S2C Fig).

Formatted: Indent: First line: 2 ch

**Antibody r40 inhibits breast cancer cell proliferation**

~~We next~~Next, we ~~tested whether these~~evaluated the anti-proliferative activity of six rabbit antibodies (r3, r4, r5, r29, r36, r40) and the mouse antibody m66 ~~possess anti-proliferative activities on~~in HER2-overexpressing ~~breast cancer cell line~~ BT-474 breast cancer cells (Fig 2A). ~~Without ligand stimulation, trastuzumab shows a more potent effect in suppressing BT-474 cell growth than pertuzumab.~~ Among ~~the~~ the tested antibodies, r40 ~~exhibited~~ showed concentration-dependent suppression of cell viability, exceeding the potency of both trastuzumab and pertuzumab (Fig 2A)~~an anti-proliferative effect comparable to pertuzumab in BT-474 cells,~~. The triple antibody combination (trastuzumab, pertuzumab and r40) enhanced inhibition at concentrations below 10 nM compared to the dual combination (trastuzumab and pertuzumab) or single agents (Fig 2A). ~~while~~ While the other five rabbit antibodies and the mouse antibody m66 ~~showed very~~exhibited weak ~~or undetectable~~ effects on ~~BT-474 cell~~ proliferation (Fig 2A). ~~Thus~~Based on these findings, r40 and m66 were ~~chosen~~ selected for further ~~study~~characterization.

In another Another two breast cancer cell lines, HER2-overexpressing –SK-BR-3 with HER2 overexpressionand low-HER2-expressing MCF7, –was also used in the cell proliferation assay. Pertuzumab was shown to inhibit SK-BR-3 cell proliferation more effectively than trastuzumab. Antibody r40 demonstrated exhibits a far superior inhibitory effectcomparable growth inhibition to than that of trastuzumab and pertuzumab (Fig 2B), while m66 has a comparable inhibition effect with trastuzumab. The triple combination exhibited enhanced or comparable efficacy relative to the dual combination –(Fig 2B).

Formatted: Indent: First line: 2 ch

With or without the activation of the NRG1 ligand, both m66 and r40 antibodies significantly inhibited cell proliferation of MCF7 with low HER2 expression. The inhibitory effect of r40 was higher than that of pertuzumab in the absence of NRG1. Antibody r40 shows a more potent effect than trastuzumab in inhibiting MCF7 cell proliferation with the activation of NRG1 ligand (Fig 2C).

The binding kinetics of trastuzumab, pertuzumab, m66 and r40 with HER2 were evaluated by Surface Plasmon Resonance Imaging (SPRi)surface plasmon resonance (SPR), which showed high affinity for r40 ($K_D$ = 0.279 nM) and trastuzumab ($K_D$ = 0.283 nM), slightly lower binding affinity for pertuzumab ($K_D$ = 2.3 nM), and modest affinity for m66 ($K_D$ = 86.4 nM) (Fig 2D2C).

Formatted: None

**Antibody r40 and m66 could bind and detect cell-surface HER2 of breast cancer cells**

Next, we tested evaluated whether the binding capacity of the rabbit antibodiesm66 and r40 could recognize to the endogenous HER2 protein of the in breast cancer cells via by flow cytometry. Both m66 and r40antibodies could detectdetected similar level of the endogenous HER2 proteins asat levels comparable to trastuzumab and pertuzumab as measured by with regards to mean fluorescence intensity MFI (Fig 3A-3C).

Formatted: Level 1

**Antibody r40 effectively blocks HER2-dependent signaling pathways**

PI3K/AKT and MAPK are the main signaling pathways downstream of the EGFR

family[1, 23]. Both trastuzumab and pertuzumab have been reported to inhibit ~~the~~ these two signaling pathways in the breast cancer cells[12, 26-28]. We evaluated some key downstream signaling molecules level upon treatment with antibodies for ~~6 h or~~ 24 h. We first assessed HER2 signaling pathway modulation in MCF7 cells after 24 h antibody treatment (5, 10, 25, 50 µg/mL) (Fig 4A). While trastuzumab, r40, trastuzumab-pertuzumab dual therapy, and triple combination all significantly reduced HER2 protein levels, neither p-AKT (S473) nor p-ERK (T202/Y204) showed significant changes (Fig 4A). Consistent with literatures, evaluating PI3K/AKT and MAPK pathways in MCF7 cells typically requires ligand stimulation (e.g., EGF or NRG1) [26, 29, 30]. Since antibodies at concentrations ranging from 5 to 50 µg/mL yielded comparable effects in reducing HER2 protein levels, we selected the concentration of 10 µg/mL (66.7 nM) for treating cells in subsequent experiments.

In BT-474 cells, trastuzumab, pertuzumab and r40 treatment reduced ~~With trastuzumab or r40 treatment, there is a reduction in pAKT~~p-AKT (S473) levels ~~in BT-474 and SK-BR-3 cells~~ (Fig 4B). ~~, whereas both pertuzumab and m66 had weak or undetectable effects on pAKT (S473) level~~Neither m66 nor r40 affected p-ERK (T202/Y204), while the triple antibody combination exhibited the lowest levels of p-AKT (S473) and p-ERK (T202/Y204) (Fig 4B).

In ~~both BT-474 and~~ SK-BR-3 cells, ~~24 h treatment with either trastuzumab or~~ r40 reduced ~~the protein level of~~ HER2~~.~~ protein levels, and ~~The~~ the triple~~combinatory use of~~ antibody combination achieved the lowest HER2 protein levels and exhibited a p-AKT (S473) effect comparable to trastuzumab and pertuzumab dual combination ~~markedly decreased pAKT (S473) and HER2 protein levels, which is consistent with their synergistic efficiency in clinical use~~ (Fig 4~~A, 4B~~C).

In conclusion, ~~without the~~in the absence of ~~activation of~~ ligand activation, ~~trastuzumab~~ r40 ~~demonstrates~~ demonstrated ~~greater~~ efficacy comparable to ~~than pertuzumab~~ trastuzumab in disrupting ~~the~~ constitutive HER2 homodimerization and heterodimerization in ~~the~~ ~~HER2-overexpressing~~breast cancer cell lines~~.~~. Furthermore, adding r40 to trastuzumab and pertuzumab enhanced their effect.

~~Next, w~~We ~~–~~next measured ~~the~~ EGF-induced EGFR/HER2 heterodimerization and downstream signaling ~~pretreated with~~following ~~antibodies~~antibody treatment. Upon EGF ~~ligand~~ activation, phosphorylation level of AKT and ERK were

Formatted: Indent: First line: 2 ch

significantly increased ~~largely~~ compared to ~~the~~ control cells (Fig 5A-5C). In MCF7 cells, r40 reduced HER2 protein levels and inhibited p-AKT (S473) (Fig 5A). In BT-474 cells, r40 showed no effect on HER2 protein levels but inhibited p-AKT (S473) and p-ERK (T202/Y204) to an extent comparable to trastuzumab and pertuzumab (Fig 5B). The triple antibody combination exhibited the most potent inhibiton of p-AKT (S473) and HER2 levels (Fig 5B). In SK-BR-3 cells, m66 inhibited p-AKT (S473) more potently than r40 (Fig 5C). Furthermore, the triple combination downregulated HER2 more effectively than the trastuzumab-pertuzumab dual combination (Fig 5C). ~~Antibody r40 exhibited a more potent ability to downregulate pAKT (S473), pERK, and HER2 than pertuzumab in BT-474 and SK-BR-3 cells.~~

~~For MCF7, m66 and r40 also exhibited pAKT inhibition effect, and trastuzumab and r40 antibody treatment obviously decreased HER2 protein level (Fig 5A).~~ Furthermore, we investigated the effect of antibodies on NRG1-induced HER2/HER3 heterodimerization and downstream signaling in ~~MCF7~~ the three breast cancer cells. In MCF7 cells, ~~Both 6 h and 24 h treatment with~~ pertuzumab completely blocked NRG1 activation (Fig 5D)~~, and~~. Antibody r40 ~~exhibited equal activity with~~was as effective as trastuzumab in reducing ~~the levels of~~ ~~pAKT~~p-AKT (S473) and p-ERK (T202/Y204) levels compared to ~~the~~ ~~control~~ NRG1 activated control cells (Fig 5D). In ~~Both~~ both ~~6 h~~BT-474 and ~~24 h~~SK-BR-3 cells, r40 and trastuzumab exerted minimal effects on p-AKT (S473), p-ERK (T202/Y204) and HER2 levels (Fig 5E, 5F). However, the triple antibody combination exhibited the most potent inhibitory effect on p-AKT (S473) and HER2 (Fig 5E, 5F). Additionally, the ~~treatment with trastuzumab or r40~~triple combination significantly reduced ~~HER2 levels,~~ p-ERK (T202/Y204) levels in BT-474 ~~and after 24 h treatment with pertuzumab or m66, HER2 levels were slightly reduced~~ cells (Fig ~~5B~~5E).

Taken together, ~~in BT-474 and SK-BR-3 cells with overexpressing HER2,~~ r40 ~~shows~~ suppressed HER2~~a considerable~~ signaling ~~reduction effect~~ in both ~~EGF~~ ligand-independent and ~~EGF~~ ligand-~~in~~dependent ~~manners~~contexts ~~.~~and enhanced the efficacy of trastuzumab and pertuzumab.~~In low HER2 expressing MCF7 cells, r40 treatment reduced HER2, pAKT, and pERK in both EGF and NRG1-dependent manners.~~

**Complex assembly and structure determination**

For the purposes of structural elucidationstudy, antibodies initially in the IgG format were converted to the Fab (fragment antigen-binding) (Fab) format (S1B Fig, S1C Fig). To obtain the trimer comprising HER2, m66 and trastuzumab, size exclusion chromatography (SEC) was conducted to obtain the binary complex of HER2 and the m66 Fab. As indicated by the SEC profiles, HER2 protein fully engaged with m66 Fab to form a binary complex, with an additional peak corresponding to the unbound m66 Fab (S2A S3A Fig). The peak of HER2-m66 binary complex was shifted forward with the addition of the trastuzumab Fab, indicating that HER2, m66 and trastuzumab formed a ternary complex (S2BS3B-S2D S3D Fig).

To obtain the tetrameric complex containing HER2, r40, trastuzumab, and pertuzumab, we first purified the binary complex containing HER2 and r40, followed by the addition of trastuzumab (S3A S4A Fig, S3B S4B Fig). The detectable shift of the SEC peak was observed with the addition of the pertuzumab Fab, indicating the presence of the larger tetrameric complex (S3CS4C-S3E S4E Fig).

Single-particle cryo-EM reconstruction was used to determine the structures of the two HER2-antibodies complexes (S4 S5 Fig, S5 S6 Fig). The cryo-EM maps of HER2-m66-trastuzumab and HER2-r40-trastuzumab-pertuzumab were refined to overall resolutions of 3.2 Å and 3.1 Å, respectively. The final structural models were built with the HER2-trastuzumab-pertuzumab structure (PDB: 6OGE){Meng, 2016 #505;Hao, 2019 #511;Hao, 2019 #511;Hao, 2019 #511;Hao, 2019 #511;Hao, 2019 #511} as a template and Alphafold2 structure prediction. For the HER2-m66-trastuzumab complex, the three components could be clearly identified in the cryo-EM map, and a final high-resolution model was built and refined (Fig 6A). Trastuzumab binds bound ECD IV and the m66 epitope of m66 was located in ECD I of HER2. We compared HER2-trastuzumab-pertuzumab (PDB: 6OGE) and HER2-m66-trastuzumab we determined with HER2 superimposed (Fig 6C6B). The binding site of pertuzumab iswas located at the dimerization interface of HER2. There would be a steric clash if m66 and pertuzumab were both bound, which explains explained why m66 and pertuzumab do didn't not bind HER2 simultaneously. Superimposition of the HER2-HER3-NRG1 structure (PDB: 7MN5)[31] and HER2-m66-trastuzumab structure shows showed that m66 is was unable to suppress HER2 homodimerization and heterodimerization (Fig 6C6B).

In the tetrameric structure of HER2-r40-trastuzumab-pertuzumab, r40 targets targeted the ECD III/IV of HER2, a site distinct from the dimerization interface of HER2 (Fig 6B6C, 6C). The superimposed structure of HER2-HER3-NRG1 (PDB: 7MN5) and HER2-r40-trastuzumab-pertuzumab structure indicates showed that the epitope of antibody r40 is was opposite to the dimerization interfaces of HER2, which indicates indicated r40 dose didn't not directly disturb the dimerization of HER2 (Fig 6C6D). The large interface between r40 and HER2 established indicates the high affinity and inhibitory activity of r40 on HER2.

## Discussion

Antibody drugs have revolutionized cancer treatment by specifically targeting tumor antigens, thereby enhancing therapeutic efficacy and reducing side effects, making them a cornerstone of modern oncology. Two approved anti-HER2 antibodies, trastuzumab and pertuzumab specifically target distinct regions of the HER2 protein: trastuzumab targets the juxtamembrane region of ECD IV, while pertuzumab binds near the core of ECD II~~ ~~[10]. Till now, no antibody therapeutics have been identified that selectively target the ECD I and ECD III of HER2. Nevertheless, ongoing studies are exploring new HER2 epitopes to develop more effective treatment strategies.

To obtain antibodies targeting distinct epitopes from those recognized by trastuzumab and pertuzumab, we performed ELISA~~ for mouse antibodies and ~~ and flow cytometry~~ for rabbit antibodies ~~ with saturated trastuzumab and pertuzumab. The data show that m66 competes with pertuzumab and r40~~ may ~~ recognizes a different epitope from that of trastuzumab and pertuzmab. The Cryo-EM structures further confirm the epitopes of m66 and r40 antibodies.

In the absence of ligands, overexpressed HER2 can form homodimers[25]. EGF and NRG1 play pivotal roles in the activation of HER2 signaling. Upon binding to EGFR, EGF triggers a conformational change that extends EGFR from a bent form to an extended state, thereby revealing its dimerization arm. This arm of HER2 then engages with the binding pocket of EGFR, establishing a stable EGFR/HER2 heterodimer[32]. NRG1, through its EGF-like domain, binds to HER3 and HER4, thereby promoting the heterodimerization of HER2/HER3 and HER2/HER4[31, 33]. These three heterodimers induce activation of the downstream PI3K/AKT and MAPK signaling pathways, thereby drive the growth of cells. In our study,~~ we demonstrated that ~~ r40 effectively ~~inhibits ~~suppresses ~~the ~~HER2 signaling~~ pathways ~~ in both ligand-independent and ligand-dependent ~~manners~~contexts, whereas m66 exerts a weak inhibitory effect.~~.~~

Trastuzumab has been shown to facilitate the internalization of HER2, directing it into endosomes, and subsequently routing it to lysosomes for degradation[34]. In both ligand-dependent and ligand-independent contexts, we observed that the r40 antibody, ~~similar to~~like trastuzumab,~~ effectively ~~ diminished the expression levels of HER2 protein in MCF7 and SK-BR-3 cells (Fig 4, Fig 5). Conversely, the m66 antibody,

similar to pertuzumab, has had noa much relatively weak influence on HER2 protein levels –(Fig 4, , Fig 5). We hypothesize that r40 induces the internalization of HER2, whereas m66 does not. One potential reason is that r40 exhibits higher affinity than m66, and the stable antigen-antibody complex structure triggers the internalization process. Another possible explanation is that the binding of m66 to ECD I does not induce conformational changes, however the epitope targeted by the r40 antibody is situated at the interface between ECD III and IV, and conformational shifts within these domains are more prone to trigger the internalization of HER2.

In summary, we screened a panel of human HER2 antibodies and characterized identifiedthat r40 is as a HER2an ECD III/IV-specific antibody, which that downregulates HER2 protein level, disruptsed PI3K/AKT and MAPK signaling pathways and suppressesd the growth of breast cancer cellscell growth. Critically, r40 enhanced the efficacy of the trastzumab-pertuzumab combination. These findings suggest Antibody r40 is a promising candidate to augment existing HER2-targeted therapies, potentially improving outcomes anticipated to enhance therapeutic efficacy when used in combination with trastuzumab and pertuzumab, suggesting its potential for HER2 HER2-overexpressing cancers. Nevertheless, to explore more functions and elucidate the complete mechanism,Future work should include more detailed mechanistic analyses and humanization of r40 will be needed in the future.

**References**

1. Citri A, Yarden Y. EGF-ERBB signalling: towards the systems level. Nat Rev Mol Cell Biol. 2006;7(7):505-16. doi: 10.1038/nrm1962. PubMed PMID: 16829981.

2. Weisser NE, Sanches M, Escobar-Cabrera E, O'Toole J, Whalen E, Chan PWY, et al. An anti-HER2 biparatopic antibody that induces unique HER2 clustering and complement-dependent cytotoxicity. Nature communications. 2023;14(1):1394-. doi: 10.1038/s41467-023-37029-3. PubMed PMID: 36914633.

3. Tzahar E, Waterman H, Chen X, Levkowitz G, Karunagaran D, Lavi S, et al. A hierarchical network of interreceptor interactions determines signal transduction by Neu differentiation factor/neuregulin and epidermal growth factor. Mol Cell Biol. 1996;16(10):5276-87. doi: 10.1128/MCB.16.10.5276. PubMed PMID: 8816440; PubMed Central PMCID: PMC231527.

4. Junttila TT, Akita RW, Parsons K, Fields C, Lewis Phillips GD, Friedman LS, et al. Ligand-independent HER2/HER3/PI3K complex is disrupted by trastuzumab and is effectively inhibited by the PI3K inhibitor GDC-0941. Cancer Cell. 2009;15(5):429-40. doi: 10.1016/j.ccr.2009.03.020. PubMed PMID: 19411071.

5. Lewis GD, Figari I, Fendly B, Wong WL, Carter P, Gorman C, et al. Differential responses of human tumor cell lines to anti-p185HER2 monoclonal antibodies. Cancer Immunol Immunother. 1993;37(4):255-63. doi: 10.1007/BF01518520. PubMed PMID: 8102322.

6. Clynes RA, Towers TL, Presta LG, Ravetch JV. Inhibitory Fc receptors modulate in vivo cytotoxicity against tumor targets. Nat Med. 2000;6(4):443-6. doi: 10.1038/74704. PubMed PMID: 10742152.

7. Nahta R, Esteva FJ. Herceptin: mechanisms of action and resistance. Cancer Lett. 2006;232(2):123-38. doi: 10.1016/j.canlet.2005.01.041. PubMed PMID: 16458110.

8. Franklin MC, Carey KD, Vajdos FF, Leahy DJ, de Vos AM, Sliwkowski MX. Insights into ErbB signaling from the structure of the ErbB2-pertuzumab complex. Cancer Cell. 2004;5(4):317-28. doi: 10.1016/s1535-6108(04)00083-2. PubMed PMID: 15093539.

9. Adams CW, Allison DE, Flagella K, Presta L, Clarke J, Dybdal N, et al. Humanization of a recombinant monoclonal antibody to produce a therapeutic HER dimerization inhibitor, pertuzumab. Cancer Immunol Immunother.

2006;55(6):717-27. doi: 10.1007/s00262-005-0058-x. PubMed PMID: 16151804.
10. Hao Y, Yu X, Bai Y, McBride HJ, Huang X. Cryo-EM Structure of HER2-trastuzumab-pertuzumab complex. PLoS One. 2019;14(5):e0216095. doi: 10.1371/journal.pone.0216095. PubMed PMID: 31042744; PubMed Central PMCID: PMC6493747.
11. Scheuer W, Friess T, Burtscher H, Bossenmaier B, Endl J, Hasmann M. Strongly enhanced antitumor activity of trastuzumab and pertuzumab combination treatment on HER2-positive human xenograft tumor models. Cancer Res. 2009;69(24):9330-6. doi: 10.1158/0008-5472.CAN-08-4597. PubMed PMID: 19934333.
12. Nahta R, Hung MC, Esteva FJ. The HER-2-targeting antibodies trastuzumab and pertuzumab synergistically inhibit the survival of breast cancer cells. Cancer Res. 2004;64(7):2343-6. doi: 10.1158/0008-5472.can-03-3856. PubMed PMID: 15059883.
13. von Minckwitz G, Procter M, de Azambuja E, Zardavas D, Benyunes M, Viale G, et al. Adjuvant Pertuzumab and Trastuzumab in Early HER2-Positive Breast Cancer. N Engl J Med. 2017;377(2):122-31. doi: 10.1056/NEJMoa1703643. PubMed PMID: 28581356; PubMed Central PMCID: PMC5538020.
14. Bilancia D, Rosati G, Dinota A, Germano D, Romano R, Manzione L. Lapatinib in breast cancer. Ann Oncol. 2007;18 Suppl 6:vi26-30. doi: 10.1093/annonc/mdm220. PubMed PMID: 17591827.
15. Sonpavde G. Lapatinib plus capecitabine in breast cancer. N Engl J Med. 2007;356(14):1471; author reply -2. doi: 10.1056/NEJMc070182. PubMed PMID: 17409332.
16. Lopez-Tarruella S, Jerez Y, Marquez-Rodas I, Martin M. Neratinib (HKI-272) in the treatment of breast cancer. Future Oncol. 2012;8(6):671-81. doi: 10.2217/fon.12.66. PubMed PMID: 22764764.
17. Verma S, Miles D, Gianni L, Krop IE, Welslau M, Baselga J, et al. Trastuzumab emtansine for HER2-positive advanced breast cancer. N Engl J Med. 2012;367(19):1783-91. doi: 10.1056/NEJMoa1209124. PubMed PMID: 23020162; PubMed Central PMCID: PMC5125250.
18. Cortes J, Hurvitz SA, Im SA, Iwata H, Curigliano G, Kim SB, et al. Trastuzumab deruxtecan versus trastuzumab emtansine in HER2-positive metastatic breast

cancer: long-term survival analysis of the DESTINY-Breast03 trial. Nat Med. 2024;30(8):2208-15. doi: 10.1038/s41591-024-03021-7. PubMed PMID: 38825627; PubMed Central PMCID: PMC11333275.
19. Oh DY, Bang YJ. HER2-targeted therapies - a role beyond breast cancer. Nature Reviews Clinical Oncology. 2020;17(1):33-48. doi: 10.1038/s41571-019-0268-3. PubMed PMID: 31548601.
20. Shitara K, Bang YJ, Iwasa S, Sugimoto N, Ryu MH, Sakai D, et al. Trastuzumab deruxtecan in HER2-positive advanced gastric cancer: exploratory biomarker analysis of the randomized, phase 2 DESTINY-Gastric01 trial. Nat Med. 2024;30(7):1933-42. doi: 10.1038/s41591-024-02992-x. PubMed PMID: 38745009; PubMed Central PMCID: PMC11271396.
21. Okines AF, Cunningham D. Trastuzumab: a novel standard option for patients with HER-2-positive advanced gastric or gastro-oesophageal junction cancer. Therap Adv Gastroenterol. 2012;5(5):301-18. doi: 10.1177/1756283X12450246. PubMed PMID: 22973416; PubMed Central PMCID: PMC3437538.
22. Rexer BN, Arteaga CL. Intrinsic and acquired resistance to HER2-targeted therapies in HER2 gene-amplified breast cancer: mechanisms and clinical implications. Crit Rev Oncog. 2012;17(1):1-16. doi: 10.1615/critrevoncog.v17.i1.20. PubMed PMID: 22471661; PubMed Central PMCID: PMC3394454.
23. Liu X, Song Y, Cheng P, Liang B, Xing D. Targeting HER2 in solid tumors: Unveiling the structure and novel epitopes. Cancer Treat Rev. 2024;130:102826. doi: 10.1016/j.ctrv.2024.102826. PubMed PMID: 39270365.
24. Pedersen MW, Jacobsen HJ, Koefoed K, Dahlman A, Kjaer I, Poulsen TT, et al. Targeting Three Distinct HER2 Domains with a Recombinant Antibody Mixture Overcomes Trastuzumab Resistance. Mol Cancer Ther. 2015;14(3):669-80. doi: 10.1158/1535-7163.MCT-14-0697. PubMed PMID: 25612619.
25. Meng Y, Zheng L, Yang Y, Wang H, Dong J, Wang C, et al. A monoclonal antibody targeting ErbB2 domain III inhibits ErbB2 signaling and suppresses the growth of ErbB2-overexpressing breast tumors. Oncogenesis. 2016;5(3):e211. doi: 10.1038/oncsis.2016.25. PubMed PMID: 26999718; PubMed Central PMCID: PMC4815051.
26. Fu W, Wang Y, Zhang Y, Xiong L, Takeda H, Ding L, et al. Insights into HER2 signaling from step-by-step optimization of anti-HER2 antibodies. MAbs.

2014;6(4):978-90. doi: 10.4161/mabs.28786. PubMed PMID: 24838231; PubMed Central PMCID: PMC4171031.
27. Tamaskovic R, Schwill M, Nagy-Davidescu G, Jost C, Schaefer DC, Verdurmen WP, et al. Intermolecular biparatopic trapping of ErbB2 prevents compensatory activation of PI3K/AKT via RAS-p110 crosstalk. Nat Commun. 2016;7:11672. doi: 10.1038/ncomms11672. PubMed PMID: 27255951; PubMed Central PMCID: PMC4895728.
28. Kast F, Schwill M, Stuber JC, Pfundstein S, Nagy-Davidescu G, Rodriguez JMM, et al. Engineering an anti-HER2 biparatopic antibody with a multimodal mechanism of action. Nat Commun. 2021;12(1):3790. doi: 10.1038/s41467-021-23948-6. PubMed PMID: 34145240; PubMed Central PMCID: PMC8213836.
29. Selitrennik M, Lev S. PYK2 integrates growth factor and cytokine receptors signaling and potentiates breast cancer invasion via a positive feedback loop. Oncotarget. 2015;6(26):22214-26. doi: 10.18632/oncotarget.4257. PubMed PMID: 26084289; PubMed Central PMCID: PMCPMC4673158.
30. Berdiel-Acer M, Maia A, Hristova Z, Borgoni S, Vetter M, Burmester S, et al. Stromal NRG1 in luminal breast cancer defines pro-fibrotic and migratory cancer-associated fibroblasts. Oncogene. 2021;40(15):2651-66. doi: 10.1038/s41388-021-01719-3. PubMed PMID: 33692466; PubMed Central PMCID: PMCPMC8049869.
31. Diwanji D, Trenker R, Thaker TM, Wang F, Agard DA, Verba KA, et al. Structures of the HER2-HER3-NRG1beta complex reveal a dynamic dimer interface. Nature. 2021;600(7888):339-43. doi: 10.1038/s41586-021-04084-z. PubMed PMID: 34759323; PubMed Central PMCID: PMC9298180.
32. Yu X, Wang L, Shen Y, Wang C, Zhang Y, Meng Y, et al. Targeting EGFR/HER2 heterodimerization with a novel anti-HER2 domain II/III antibody. Molecular Immunology. 2017;87:300-7. doi: 10.1016/j.molimm.2017.05.010. PubMed PMID: 28531814.
33. Trenker R, Diwanji D, Bingham T, Verba KA, Jura N. Structural dynamics of the active HER4 and HER2/HER4 complexes is finely tuned by different growth factors and glycosylation. Elife. 2024;12. doi: 10.7554/eLife.92873. PubMed PMID: 38498590; PubMed Central PMCID: PMC10948148.
34. Baselga J, Albanell J. Mechanism of action of anti-HER2 monoclonal

antibodies. Ann Oncol. 2001;12 Suppl 1:S35-41. doi: 10.1093/annonc/12.suppl_1.s35. PubMed PMID: 11521720.
35. Zheng SQ, Palovcak E, Armache JP, Verba KA, Cheng Y, Agard DA. MotionCor2: anisotropic correction of beam-induced motion for improved cryo-electron microscopy. Nat Methods. 2017;14(4):331-2. doi: 10.1038/nmeth.4193. PubMed PMID: 28250466; PubMed Central PMCID: PMC5494038.
36. Zhang K. Gctf: Real-time CTF determination and correction. J Struct Biol. 2016;193(1):1-12. doi: 10.1016/j.jsb.2015.11.003. PubMed PMID: 26592709; PubMed Central PMCID: PMC4711343.
37. Scheres SH. RELION: implementation of a Bayesian approach to cryo-EM structure determination. J Struct Biol. 2012;180(3):519-30. doi: 10.1016/j.jsb.2012.09.006. PubMed PMID: 23000701; PubMed Central PMCID: PMC3690530.
38. Kimanius D, Forsberg BO, Scheres SH, Lindahl E. Accelerated cryo-EM structure determination with parallelisation using GPUs in RELION-2. Elife. 2016;5. doi: 10.7554/eLife.18722. PubMed PMID: 27845625; PubMed Central PMCID: PMC5310839.
39. Punjani A, Rubinstein JL, Fleet DJ, Brubaker MA. cryoSPARC: algorithms for rapid unsupervised cryo-EM structure determination. Nat Methods. 2017;14(3):290-6. doi: 10.1038/nmeth.4169. PubMed PMID: 28165473.
40. Pettersen EF, Goddard TD, Huang CC, Couch GS, Greenblatt DM, Meng EC, et al. UCSF Chimera--a visualization system for exploratory research and analysis. J Comput Chem. 2004;25(13):1605-12. doi: 10.1002/jcc.20084. PubMed PMID: 15264254.
41. Tunyasuvunakool K, Adler J, Wu Z, Green T, Zielinski M, Zidek A, et al. Highly accurate protein structure prediction for the human proteome. Nature. 2021;596(7873):590-6. doi: 10.1038/s41586-021-03828-1. PubMed PMID: 34293799; PubMed Central PMCID: PMC8387240.
42. Emsley P, Cowtan K. Coot: model-building tools for molecular graphics. Acta Crystallogr D Biol Crystallogr. 2004;60(Pt 12 Pt 1):2126-32. doi: 10.1107/S0907444904019158. PubMed PMID: 15572765.

**Acknowledgments**

We thank the Center of Cryo-Electron Microscopy, Core Facility of Shanghai Medical College, Fudan University for the supports on cryo-EM data collection and data analyses. ~~This work was supported by grants from the Shanghai Natural Science Foundation of China (21ZR1407900).~~

**Author contributions**

H. Y. and Y. X. supervised the project; C. L. purified the antibodies and conducted the sample preparation for structural and biochemical studies; C. L. and X. L. performed the cell assay; Y. R. and Q. W. conducted cryo-EM data collection, data analyses and model building; D. Z., P. Z., X. D. screened the antibodies; X. H. helped the flow cytometry assay; C. L. and H. Y. performed data analysis, genetated the figures and drafted the manuscript with contributions from Y. X.

**Competing interests**

Authors declare no competing interests.

**Data and materials availability**

The cryo-EM density maps and corresponding atomic coordinates will be made available upon manuscript acceptance.

Formatted: Justified

# Materials and Methods

## Cell culture

BT-474, SK-BR-3 and MCF7 were acquired from the American Type Culture Collection (ATCC). These cells were maintained in DMEM medium (Gibco) supplemented with 10% fetal bovine serum (NEWZERUM) and penicillin/streptomycin (Basalmedia) at the concentration of 100 μg/mL. Expi293 cells were cultured in Union 293 cell feed medium (Union) containing 100 μg/mL penicillin/streptomycin.

## Construction and expression

The DNA sequence encoding human HER2 ECD (residues 23-652) was amplified and inserted into a modified pCAG vector containing a signal peptide and an N-terminal 9× His tag, enabling the production of a secreted HER2 ECD fusion protein. There is a HRV 3C protease recognition site between the His tag and HER2 ECD sequence (S1A Fig). The DNA sequence encoding the 55 amino-acid EGF like domain of human NRG1 (GTSHLVKCAEKEKTFCVNGGECFMVKDLSNPSRYLCKCPNEFTGDRCQNYVMASF) was synthesized and cloned into the same modified pCAG vector (S1A Fig). The fusion proteins were purified from the culture medium of Expi293 cells using Ni-NTA Sepharose Fast Flow (GE Healthcare), and subsequently cleaved with HRV 3C protease to remove the His tag.

## Kinetics and affinity measurement

Binding kinetics of antibodies with HER2 were determined by SPRi using a PlexArray system (Plexera). IgG format antibodies (~~50~~ 0.5 ~~μg~~mg/mL) were immobilized on an 3D optical crosslinking biosensor, and then a of serial dilutions of HER2 (1-128 nM) in PBS were subjected to a process of 285 s association and 930 s dissociation.

$EC_{50}$ of m66 and r40 was measured with ELISA. In brief, ELISA plates (Corning) were coated with 0.2 ng/μL of HER2 solution overnight at 4 °C. After blocking, a serial of concentrations ($2^{-11}$-$2^{3}$ ng/μL) were added to the plate and incubated for another 1 h at 37 °C. Either HRP-conjugated goat anti-human IgG1 Fc antibody (Millipore, AP113P) or HRP-conjugated goat anti-rabbit IgG (H+L) antibody (Abclonal, AS014) was incubated for 30 min-1 h at 37 °C. After washing with PBST, TMB substrate

(Beyotime, P0206) was added. The reaction was quenched with 1 M HCl, followed by absorbance measurement at 450 nm using a microplate reader (Biotek). $EC_{50}$ estimates were obtained by fitting ELISA binding curves in Graphpad Prism using the log versus response-variable slope model.

**Competitive binding assayEpitope mapping**

To map epitopes of two mouse antibodies, HER2 ECD protein was diluted to 0.2 µg/mL in 1× PBS and incubated overnight at 4°C. After washing with PBST buffer, non-His-tagged IgG was diluted to 2 µg/mL in blocking buffer (PBST + 3% BSA) and incubated with the immobilized HER2 at 37°C for 1 h. Without discarding the non-His-tagged IgG, 2 µg/mL His-tagged antibodies was immediately added to the wells. Incubation continued at 37°C for 30 min with agitation. After washingwas blocking with saturating non-His-tagged IgG, the subsequent binding of His-tagged IgG protein was measured with anti-his-HRP (Genscript, A00612) using ELISA.

To preliminarily characterize the epitopes of rabbit antibodies, Expi 293 cells were suspended in FACS buffer (DPBS + 2% FBS) at 1× $10^7$ cells/mL with 1:50 Human TruStain FcX™ (Biolegend, 422302) and , then incubated with or without saturating concentrations10 µg/mL (66.7 nM) of trastuzumab and pertuzumab for 0.5 h30 min. After washing, the cells were further incubated with 10 µg/mL rabbit monoclonal antibodies for additional 0.5 h30 min. Cells were stained with AF488-conjugated goat anti-rabbit IgG secondary antibody (Invitrogen, A11008), and subsequently analyzed using a flow cytometer (Thermo Fisher Scientific) and the FlowJo FlowJo 10.8.1 software.

To determine the saturation concentrations for the binding of trastuzumab and pertuzumab to HER2 receptors on the cell surface of MCF7, 1× $10^5$ MCF7 cells were incubated with trastuzumab and pertuzumab (0-400 nM) for 30 min. Subsequently, cells were stained with AF647-conjugated goat anti-human secondary antibody (Invitrogen, A21445) and analyzed by flow cytometry.

The two mouse antibodies (m66, m75) and 7 rabbit antibodies (r3, r4, r5, r29, r35, r36, r40) were labeled by AF647 dye functionalized with an NHS Ester. The antibodis were incubated with 20 molar equivalents of AF647 NHS ester (Biotium, 30-3007) for 2 h at room temperature protected from light. The crude conjugation mixture was

Formatted: Font: (Default) Times New Roman

Formatted: Font: (Default) Times New Roman

Formatted: Font: (Default) Times New Roman

Formatted: Font: (Default) Times New Roman

Formatted: Font: (Default) Times New Roman

Formatted: Font: (Default) Times New Roman

dialyzed against PBS (pH 7.4) to remove the excess dye. The 1:3 serial dilution of AF647 labeled antibodies (0-300 nM) were incubated with or without 267 nM (40 μg/mL) trastuzumab and/or pertuzumab pre-saturated MCF7 cells.

**Tumor cell binding by Flow flow cytometric analysis**

BT-474, SK-BR-3 and MCF7 were briefly trypsinized at 37°C, quenched with FBS-containing medium and removed by centrifugation. A number of $1\times 10^5$600,000 cells for each sample were suspended in FACS buffer with Human TruStain FcX™ (1× PBS containing 2% FBS), followed by incubation with 10 μg/mL267 nM (40 μg/mL) trastuzumab, pertuzumab, m66, r40 for 0.5 h30 min - 1 h at 4 °C. Human IgG isotype (Beyotime, A7001) and rabbit IgG isotype (Beyotime, A7016) were used as the isotype control. Then the cells were incubated for an additional 0.5 h30 min with either AF647-conjugated goat anti-human secondary antibody (Invitrogen, A21445) or AF488-conjugated goat anti-rabbit secondary antibody (Invitrogen, A11008). Cells were analyzed via flow cytometery cytometry after staining, and histograms of anti-HER2 antibodies were compared with their respective isotype controls.

**Cell proliferation assay**

1-29$\times10^3$ BT-474, $3\times10^3$ SK-BR-3 and $5\times10^3$ MCF7 cells were plated in the flatbottomed 96-well cell culture plates overnight and incubated with a 1:3 serial dilution of antibodies (0-100 nM) for 4 d. After treatment, antibody-mediated cell growth inhibitionand incubated overnight. Gradient concentrations of antibodies (100, 20, 4, 0.8, 0.16, 0 μg/mL) were added to the wells. Cell proliferation was quantitatively determined using Cell Counting Kit-8 ((Selleck, B34302) with a microplate reader Synergy H1 (BioTek).Beyotime, C0042) following an additional 4-day incubation.

**Immunoblotting**

For ligand-independent HER2 signaling, BT-474, SK-BR-3 and MCF7 cells were treated with 5, 10, 25, 50 μg/mL mL (33.3, 66.7, 166.7, 333.3 nM) trastuzumab, pertuzumab, m66, r40, or the combination of trastuzumab and pertuzumab or the combination of trastuzumab, pertuzumab and r40 (each at 50 μg/mL) (each at 5, 10, 25, 50 μg/mL) for either 6 h or 24 h. BT-474 and SK-BR-3 cells were treated with 10 μg/mL trastuzumab, pertuzumab, m66, r40, the combination of trastuzumab and pertuzumab or the combination of trastuzumab, pertuzumab and r40 (each at 10 μg/mL)

Formatted: Font: (Default) Times New Roman

Formatted: Font: (Default) Times New Roman

Formatted: Font: (Default) Times New Roman

Formatted: Font: 12 pt, Not Bold

Formatted: Font: 12 pt

for 24 h. For ligand-dependent HER2 signaling, Recombinant recombinant human NRG1 or EGF (Acro biosystems, EGF-H52H3) were supplemented at the final concentration of 0.5 nM and 5 nM respectively for 10 min after the 10 μg/mL antibodies treatment for 24 h. Following washing with ice-cold 1× PBS, the cells were lysed and the cell lysates were harvested and analyzed using reducing 4-20% gradient SDS-PAGE, followed by immunoblotting with antibodies targeting GAPDH (Beyotime, AF0006), Tubulin (Abelonal, AC012), AKT (Cell Signaling Technology, 9272), phospho-AKT (Ser473) (Cell Signaling Technology, 4058), p44/42 MAPK (Erk1/2) (Cell Signaling Technology, 4695), phospho-p44/42 MAPK (Erk1/2) (Cell Signaling Technology, 4370) or HER2 (Cell Signaling Technology, 4290).

**Fab purification and complex formation**

The heavy chain and light chain expression cassettes of four Fabs (trastuzumab, pertuzumab, m66 and r40) were assembled into a redesigned pCAG vector. One 9× His tag was incorporated into the C-terminal of heavy chain constant domain. Fab fragments were obtained by co-transfection of light chain and heavy chain into suspension Expi 293 cells using PEI (Yeasen, 40816ES08). Following Ni-NTA purification, eluent was dialyzed to remove imidazole.

For HER2-m66-trastuzumab complex, HER2 was pretreated with an excess amount of m66 Fab. The resulting mixture was seperated separated by SEC using a Superdex 200 10/300 column (GE Healthcare) in 20 mM HEPES pH 7.4, 150 mM NaCl. The HER2-m66 binary complex was collected and further incubated with excessive trastuzumab Fab to form the HER2-m66-trastuzumab complex. HER2-r40-trastuzumab-pertuzumab complex was also prepared using a similar procedure.

**Cryo-EM sample preparation and data collection**

For cryo-EM grid preparation, 3 μL of 1.5 mg/mL HER2-m66-trastuzumab and HER2-r40-trastuzuambtrastuzumab-pertuzumab were loaded onto the freshly glow-discharged Quantifoil R1.2/1.3 Au grids. Samples were vitrified by a FEI Vitrobot Mark IV with one second blot time at 100% humidity. Subsequently, the grids were applied to a Titan Krios G3i or G4 transmission electron microscope (Thermo Fisher Scientific) with 300 kV accelerating voltage for data collection and characterization. In total, 992 micrographs of HER2-r40-trastuzumab-pertuzumab were automatically recorded via a

Titan Krios G3i transmission electron microscope equipped with Gatan K3 direct electron detector for characterization. Micrographs of HER2-r40-trastuzumab-pertuzumab were captured with Serial-EM software in the super-resolution counting mode. The instrument was operated at a ~~norminal~~ nominal magnification of 81,000× in the EFTEM mode, leading to a super-resolution pixel size of 0.532 Å on the image plane. The defocus values spanned a range of -1.5 µm to -2.5 µm. The micrograph stack was dose-divided into 40 individual frames for dose-per-frame, with each frame receiving approximately 50 e-/ Å$^2$ and 2.3 s exposure time. The other images were captured automatically using a Titan Krios G4 transmission electron microscope equipped with a Falcon 4i direct electron detector. The micrographs were recorded in super-resolution counting mode using the EPU software at a ~~norminal~~ nominal magnification of 130,000× in the NPTEM mode. This configuration achieved a super-resolution pixel size of 0.466 Å on the image plane. With defocus values spanning between -1.5 µm to -2.5 µm, each micrograph stack was dose-fractionated into 1080 frames, receiving approximately 50 e-/ Å$^2$ total electron dose, under 3.51 s exposure time. For further processing, 2,439 and 2,965 micrographs were systematically acquired for the datasets of HER2-m66-trastuzumab and HER2-r40-trastuzumab-pertuzumab, respectively.

**Cryo-EM data processing**

The processing of all cryo-EM datasets involved addressing and correcting drift and beam-induced motion corrections to achieve whole-frame movie alignment via MotionCor2[35]. The micrographs from the Titan Krios G3i were subjected to a binning process with a factor of 2 to achieve a calibrated pixel size of 1.064 Å/pixel, while the Titan Krios G4 datasets were binned similarly and down to 0.932 Å/pixel. Defocus values were determined using Gctf[36] on summed image data, with dose weighting not applied. Subsequent cryo-EM data were processed using RELION v3.1[37, 38] and cryoSPARC v2[39], employing dose-weighted images for analysis.

For HER2-m66-trastuzumab dataset, 2,017,757 particles were automatically picked in RELION with no reference and subsequently subjected to reference-free 2D classification in cryoSPARC. In total, 466,601 particles were selected from good 2D classes for the 3D initial model. In all, 584,245 were selected from high-quality 3D classes and utilized for heterogeneous refinement in cryoSPARC. The refined particles

were loaded to RELION again for 2D and 3D classification. A total of 282,644 particles were extracted from well-defined 3D class assignments and subjected to heterogeneous refinement in cryoSPARC, with CTF correction, achieving the HER2-m66-trastuzumab complex at 3.2 Å resolution.

For the dataset of HER2-r40-trastuzumab-pertuzumab acquired using the Titan Krios G3i, an automatic particle selection process yielded 1,490,027 particles, followed by 2D classification analysis using RELION. From the well-characterized 2D classes, 56,640 particles were chosen to initiate the 3D classification process. Ultimately, 24,229 particles were extracted from high-quality 3D classes and subjected to 3D auto-refinement, achieving a 9.1 Å resolution reconstruction of the HER2-r40-trastuzumab-pertuzumab complex.

For the dataset of HER2-r40-trastuzumab-pertuzumab from Titan Krios G4, leveraging the previously achieved 9.1 Å resolution structure as a template, 2,293,703 particles were automatically selected and underwent 2D classification using RELION. From the well-defined 2D classes, a selection of 1,973,712 particles was made to proceed with the preliminary 3D classification analysis. A total of 450,219 particles were extracted from high-quality 3D classes and subsequently utilized for 3D auto-refinement. After undergoing Bayesian polishing, post-processing and CTF refinement, this process achieved a 3.1 Å resolution reconstruction of the HER2-r40-trastuzumab-pertuzumab complex.

All observed resolutions were established in accordance with the gold-standard Fourier shell correlation (FSC) criterion of 0.143 and the gold-standard FSC curves were validated to address the effects of a soft mask with high-resolution noise substitution. The cryo-EM maps were processed within the cryoSPARC software using module-based tools for B-factor optimization. 3D volume mapping was visualized and evaluated using UCSF Chimera or UCSF ChimeraX[40], with local resolution variations determined via RELION analysis.

**Model building and structure refinement**

With the structure of HER2-trastuzumab-pertuzumab (PDB: 6OGE)[10] serving as the initial structural template, the cryo-EM maps were utilized for model fitting. The HER2-trastuzumab ~~compenent~~ component and the m66 structure from AlphaFold

prediction[41] were docked into corresponding cryo-EM maps of HER2-m66-trastuzumab via rigid-body fitting in UCSF Chimera[40]. The HER2-trastuzumab-pertuzumab structure (PDB: 6OGE) and the r40 structure predicted by AlphaFold were incorporated into the cryo-EM maps of HER2-r40-trastuzumab-pertuzumab using UCSF Chimera for rigid-body fitting. Structural models were generated using COOT[42]. Maps and models in the figures were created using PyMOL (https://pymol.org/), UCSF Chimera, or UCSF ChimeraX. Statistics of the map reconstruction and model refinement with detailed parameters are available in S1 Table.

## Figure Legends

**Fig. 1. Relative epitopes were assessed by ELISA and flow cytometry.** A. Binding of His-tagged IgG to immobilized HER2 was assessed with anti-His-HRP via ELISA under conditions with or without saturating concentrations of non-His-tagged IgG. B. – Saturating concentrations of trastuzumab and pertuzumab for binding to HER2 on live MCF7 cells were determined. MCF7 cells were incubated with trastuzumab and pertuzumab (0-400 nM) for 30 min, then stained with AF647-conjugated goat anti-human secondary antibody for flow cytometry analysis. The binding of rabbit antibodies to HER2 on the cell surface of Expi293 cells was evaluated, with results compared to those obtained using isotype control IgG alone. C. – MCF7 cells were pre-incubated with supersaturating concentration (267 nM) of trastuzumab and pertuzumab, and the binding of 1-300 nM trastuzumab-AF647 and pertuzumab-AF647 was evaluated by flow cytometry. Flow cytometry results for six rabbit antibodies, with or without saturating concentrations of trastuzumab and pertuzumab. D. Binding of m66-AF647 and m75-AF647 to MCF7 that were pre-incubated with supersaturating concentration (267 nM) of trastuzumab or pertuzumab was evaluated by flow cytometry. E. Binding of r35-AF647 and r40-AF647 to MCF7 that were pre-incubated with supersaturating concentration (267 nM) of trastuzumab or pertuzumab was evaluated by flow cytometry. T, trastuzumab; P, pertuzumab. Rabbit antibodies epitope mapping by flow cytometry. The x-axis represents the clone identification of rabbit antibodies, while the y-axis shows the ratio of the mean fluorescence, comparing the presence and absence of saturating concentrations of trastuzumab and pertuzumab. Error bars, SD. *, $P < 0.05$; **, $P < 0.01$; ***, $P < 0.001$; ****, $P < 0.0001$, T-test.

**Fig. 2. HER2-expressing tumor cell proliferation inhibition and $K_D$ measurement of m66 and r40.** A. BT-474 cells were treated with a 1:3 serial dilution of antibodies (0-100 nM) for 4 d. Cell viability was determined by CCK8 assay. Cell proliferation inhibition activity of nine antibodies and the combination of trastuzumab and pertuzumab were measured by CCK8 assay in BT-474. B. The inhibitory effects on cell proliferation induced by antibodies were conducted in SK-BR-3 and MCF7 cells with a 1:3 serial dilution of antibodies (0-100 nM) treatment for 4 d. Data are mean ± SEM from n = 5 (BT-474 and SK-BR-3) and n = 6 (SK-BR-3) independent experiments. C. NRG1-independent and NRG1-dependent tumor cell proliferation was conducted in MCF7 cells. Results are depicted as the percentage relative of control cell proliferation. C, control; T, trastuzumab; P, pertuzumab. Error bars, SD. *, $P < 0.05$; **, $P < 0.01$; ***, $P < 0.001$; ****, $P < 0.0001$, T-test. D.

Formatted: Font: 10.5 pt

Formatted: Font: 10.5 pt

Formatted: Font: 10.5 pt

Formatted: Font: 10.5 pt

Formatted: Font: 10.5 pt

Formatted: Font: 10.5 pt

Dissociation constant measurement of trastuzumab, pertuzumab, m66 and r40 against HER2 by SPRi assay. Trastuzumab, pertuzumab, m66 and r40 at the concentration of 0.5 mg/mL were immobilized on an 3D optical crosslinking biosensor (Plexera), and then a of serial dilutions of HER2 (1-128 nM) in PBS were subjected to a process of 285 s association and 930 s dissociation by PlexArray® HT system (Plexera).

**Fig. 3. Binding profiles of trastuzumab, pertuzumab, m66 and r40 to tumor cell lines expressing HER2.** BT-474 (A), SK-BR-3 (B) and MCF7 (C) were incubated with 267 nM (~~10~~ 40 μg/mL) of the antibodies, followed by staining with fluorescence-labeled secondary antibodies. The samples were analyzed via flow cytometry.

**Fig. 4. Antibody r40 inhibits ligand-independent HER2 signaling in BT-474 and SK-BR-3.** A. Immunoblots assessing HER2 signaling upon treatment with 5, 10, 25, 50 μg/mL (33.3, 66.7, 166.7, 333.3 nM) antibodies for 24 h in MCF7. Cell lysates were immunoblotted to detect p-AKT (Ser473), total AKT, p-ERK (T202/Y204), total ERK, HER2, GAPDH. Immunoblots assessing HER2 signaling upon treatment with ~~50~~ 10 μg/mL (66.7 nM) antibodies for ~~6 h or~~ 24 h in BT-474 (~~A~~B) and SK-BR-3 (~~B~~C). For the antibody combination ~~treatment of trastuzumab and pertuzumab~~, the final concentration of each antibody is 5, 10, 25, 50 μg/mL for MCF7 and ~~50~~ 10 μg/mL for BT-474 and SK-BR-3.

**Fig. 5. Antibody r40 inhibits ligand-dependent signaling.** ~~A.~~ Immunoblots assessing HER2 downstream signaling in MCF7 (A)~~BT-474~~, BT-474 (B)~~SK-BR-3~~ and SK-BR-3 (C) ~~MCF7~~ upon incubation with ~~50~~ 10 μg/mL (66.7 nM) antibodies for 24 h under 5 nM EGF stimulation for 10 min. ~~B.~~ Immunoblots assessing HER2 downstream signaling in MCF7 (D), BT-474 (E) and SK-BR-3 (F)~~MCF7~~ cells with ~~50~~ 10 μg/mL (66.7 nM) antibodies treatment for ~~6 h or~~ 24 h ~~in the presence of~~ under 0.5 nM NRG1 stimulation for 10 min.

**Fig. 6. Cryo-EM structures of HER2-m66-trastuzumab and HER2-r40-trastuzumab-pertuzumab are determined.** A. Cryo-EM map depicting the the complex composed of three components: HER2 (sky blue), m66 Fab (orange), and trastuzumab Fab (pink), and the final model (grey surface) incorporating the three components into the map. B. Cryo-EM map

Formatted: Font: 10.5 pt

Formatted: Font: 10.5 pt

Formatted: Superscript

Formatted: Font: 10.5 pt

Formatted: Font: Not Bold

~~illustrating~~ illustrated the tetrameric complex consisting of four components: HER2 (sky blue), r40 Fab (green), trastuzumab Fab (pink) and pertuzumab Fab (yellow), and the final construction (grey surface) with the four proteins fitted into the map. C. Superposition of HER2-trastuzumab-pertuzumab structure (PDB: 6OGE) and HER2-m66-trastuzumab structure. Superposition of HER2-HER3-NRG1 structure (PDB: 7MN5) and HER2-m66-trastuzumab structure. Overall structure of HER2-r40-trastuzumab-pertuzumab. Superposition of HER2-HER3-NRG1 structure (PDB: 7MN5) and HER2-r40-trastuzumab-pertuzumab complex.

1. **Antibody generation:**

   a. **What formats were the recombinant antibodies produced using the selected sequences from B cells?**

For cryo-EM structural characterization, we employed Fab fragments; all functional experiments utilized full-length IgG antibodies.

   b. **Protocols for ELISA and "pull-down" screening assays have to be provided.**

Thanks for the kind reminder, we have provided the protocols for ELISA and pull-down as follows:

ELISA

HER2 ECD protein was diluted to 0.2 μg/mL in 1× PBS and incubated overnight at 4°C. After blocking, the medium of recombinant expression IgG was diluted 1:100 in blocking buffer (PBST + 3% BSA) and incubated for 1 h. After washing, the binding of the recombinant expression IgGs was measured with HRP-conjugated goat anti-human IgG1 Fc antibody (Millipore, AP113P) or HRP-conjugated goat anti-rabbit IgG (H+L) antibody (Abclonal, AS014).

Pull-down

20 μL of 20% slurry rProtein A/G magnetic beads (SMART, SM015010), pre-washed with washing buffer (PBS + 0.1% CHAPS), were incubated with 10 μg of antibody at room temperature for 1 h. The beads were then washed three times with washing buffer and aliquoted equally into two portions. One portion served as a negative control, the other portion was incubated with 20 μg HER2 ECD protein at room temperature for 1 h and washed three times with washing buffer, followed by SDS-PAGE analysis.

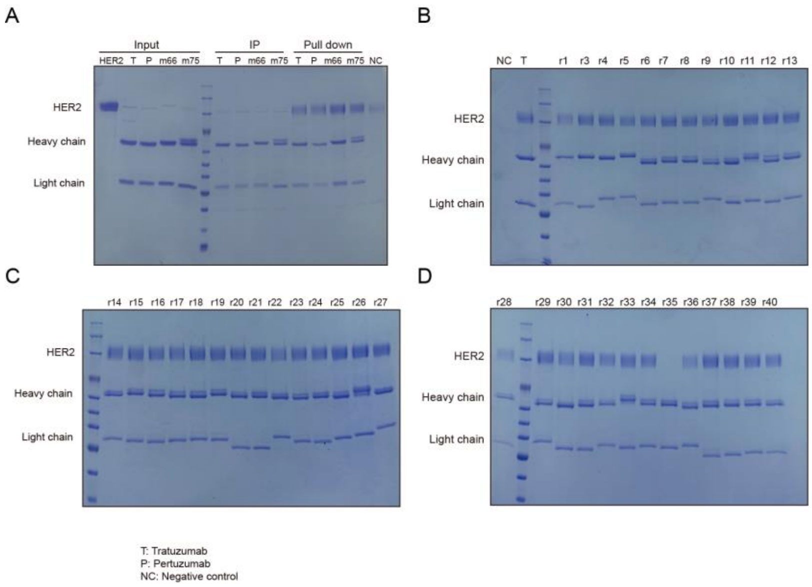


Fig. 1. A. SDS-PAGE of mouse antibody pull-down. B-D. SDS-PAGE of rabbit antibody pull-down.

In Figure 1, panel A demonstrates pull-down assay results for mouse antibodies m66 and m75, while panel B-D show results for the 39 rabbit antibodies. All antibodies except r35 demonstrated antigen-positive binding.

c. **How did the authors confirmed selectivity and specificity of the antibodies?**

We assessed the selectivity and specificity of the antibodies by ELISA, pull-down, SPRi and flow cytometry assays, which show that the screened antibodies are specific for HER2.

## 2. Competition assay

a. **What is "supersaturating IgG" [concentration] – how it was determined? What was the concentration of His-tagged IgG? There was no single dose-response binding curve for any reported antibody in the whole manuscript.**

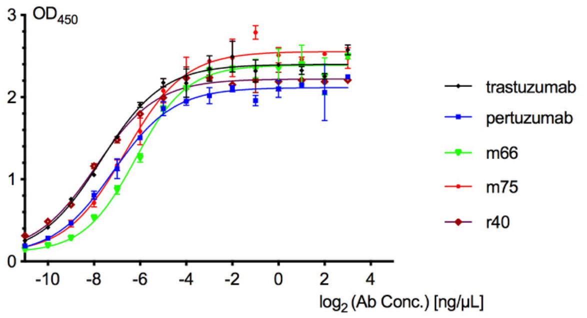


| Antibody | $EC_{50}$ (ng/mL) |
|---|---|
| trastuzumab | 4.837 |
| pertuzumab | 6.954 |
| m66 | 13.59 |
| m75 | 9.973 |
| r40 | 4.146 |

Fig. 2. The EC50 values of trastuzumab, pertuzumab, m66, m75 and r40 with immobilized HER2 ECD protein was determined by ELISA.

The $EC_{50}$ values of trastuzumab, pertuzumab, m66, m75 were all at the ng/mL level (Figure 2). Therefore, 2 μg/mL non-His-tagged IgG is sufficient to completely block all corresponding binding epitopes on HER2 ECD protein.

To map epitopes of mouse antibodies by ELISA, HER2 ECD protein was diluted to 0.2 μg/mL in 1× PBS and incubated overnight at 4°C. After blocking, non-His-tagged IgG was diluted to 2 μg/mL in blocking buffer and incubated with the immobilized HER2 at 37°C for 1 h. Without discarding the non-His-tagged IgG, 2 μg/mL His-tagged antibodies was immediately added to all wells. Incubation continued at 37°C for 30 min with agitation. After washing, the subsequent binding of His-tagged IgG protein was measured with anti-his-HRP (Genscript, A00612) using ELISA.

**b. Why mouse antibodies were tested via ELISA, and rabbit – via flow cytometry?**

Both ELISA and flow cytometry can be employed to determine antibody epitopes. Given the limited number of mouse antibodies (only two in this study),

ELISA was selected for its practical advantages in small-scale analysis. Conversely, the high quantity of rabbit antibodies made flow cytometry a more streamlined approach for large-scale screening.

In the revised manuscript, we have supplemented epitope binning experiments for both mouse and rabbit antibodies via flow cytometry. Because the variable regions of the two mouse antibodies were also cloned into a human IgG1 backbone, they could not be detected with human secondary antibodies to assess binding to trastuzumab/pertuzumab-saturated MCF7 cells. Thus, we directly conjugated both the mouse and rabbit antibodies with AF647 fluorescent dye using NHS ester chemistry.

c. **Flow cytometry competitive experiment was not described adequately in the Results section. If the authors used transient transfection, how was the efficiency of the transfection tested? What was the concentrations for all used antibodies? How were these concentrations determined?**

Since we can detect endogenous HER2 protein expression in Expi293 cells, we didn’t transiently transfect HER2 plasmid for flow cytometry assay.

We titrated the required concentrations of trastuzumab and pertuzumab to saturate HER2 on Expi293 cells, and found that neither 10 µg/mL nor even 20 µg/mL antibodies could fully saturate all HER2 proteins on the cell surface (Figure 3). Given that Expi293 is neither a breast cancer cell line nor an established model for cell-surface HER2 expression, we switched to MCF7 breast cancer cells (with low HER2 expression) for epitope binning in the revised manuscript.

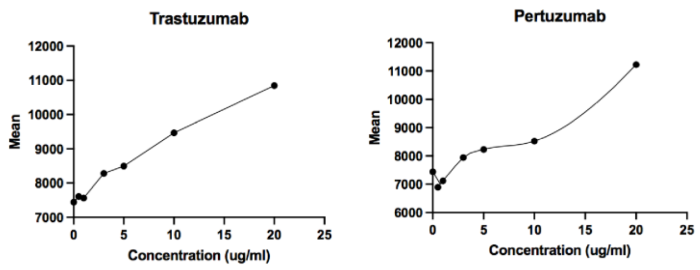


Fig. 3. The concentration of trastuzumab and pertuzumab required to saturate HER2 on Expi293 cells was determined using flow cytometry.

We titrated trastuzumab and pertuzumab on MCF7 cells to determine saturation levels and found that 100 nM sufficiently saturated all HER2 proteins on the MCF7 cell surface (Figure 4 in this response letter, same with Figure 1B in the revised manuscript). In subsequent flow cytometry experiments, cells were blocked with 267 nM (40 μg/mL) trastuzumab and pertuzumab, followed by assessment of 267 nM (40 μg/mL) mouse and rabbit antibody binding.

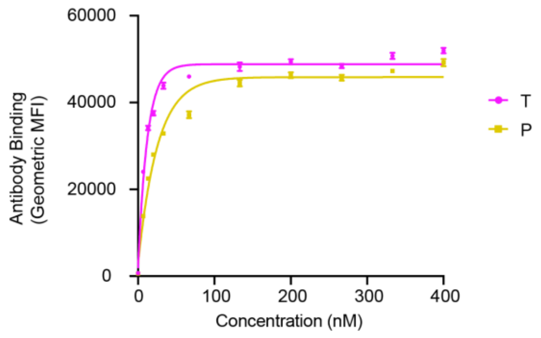


Fig. 4. The concentration of trastuzumab and pertuzumab required to saturate HER2 on MCF7 cells was determined using flow cytometry.

Although 10 μg/mL antibodies did not achieve complete HER2 saturation on Expi293 cells, the results remain biologically informative. Based on the Expi293 epitope binning data, we selected one negative control antibody (r35) and six antibodies (r3, r4, r5, r29, r36, r40) with epitopes distinct from trastuzumab/pertuzumab for validation in MCF7 cells. The binding profiles ultimately aligned with results from Expi293 cells (Figure 1E, Figure S2C in the revised manuscript).

**Inhibition breast cancer cell proliferation assay**

**d. There was no figure that allows to compare the levels of HER2 expression in three cell line used. Binding dose-response curves with tested antibodies (including clinical references trastuzumab and pertuzumab) are required for each of the cell lines before the other experiments are performed. The data presented on Figure 3 are not sufficient do describe the tested antibodies binding abilities.**

We didn't compare the levels of HER2 expression in the three cell lines we used because we can get the expression information from these aspects. First, the Figure 1 of this paper (doi: 10.1155/2012/486568) and Table 1 and Supplementary Table 4 of the paper (doi: 10.1038/s41467-023-37029-3) also can give the conclusion that HER2 protein level is lower in MCF7 cells than BT-474 and SKBR3 cells.

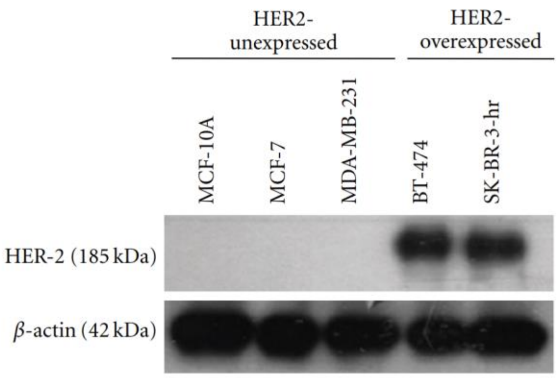


Figure 1: The expression of HER-2 oncoprotein in various human breast cancer cell lines. Cells were lysed and analyzed by western blot as described in methods. As revealed in figure, BT-474 and SKBr-3-hr were HER-2-overexpressed breast cancer cell lines. Whiles MCF-7, MCF-10A, and MDA-MB-231 cells were HER-2-unexpressed breast cancer cells.

Figure 1 of the paper (doi: 10.1155/2012/486568)

**Table 1 | Percent complement-dependent cytotoxicity at maximal effect values of anti-HER2 Abs in HER2 expressing cancer cell lines**

| Cell line | Cancer type | HER2 IHC overall score[a] | Average HER2 receptors/cell[b] | Percent of viable cells remaining following treatment at maximal effect (95% CI)[c] | | | | |
|---|---|---|---|---|---|---|---|---|
| | | | | Zanidatamab | Trastuzumab | Pertuzumab | Tras + pert | Negative control |
| HCC1954 | Breast | 3+ | $7.03\times10^6$ | 49.8 (46, 54) | Inactive | Inactive | Inactive | Inactive |
| HCC1419 | Breast | 3+ | $6.58\times10^6$ | 6.18 (2.3, 10) | Inactive | Inactive | Inactive | Inactive |
| ZR-75-30 | Breast | 3+ | $5.36\times10^6$ | 4.07 (1.7, 6.4) | Inactive | Inactive | Inactive | Inactive |
| AU565 | Breast | 3+ | $4.54\times10^6$ | 34.3 (28, 40) | Inactive | Inactive | Inactive | Inactive |
| NCI-H2170 | Lung | 3+ | $4.40\times10^6$ | 20.6 (16, 26) | Inactive | Inactive | Inactive | Inactive |
| OE-19 | Esophageal | 3+ | $3.44\times10^6$ | 24.6 (21, 28) | Inactive | Inactive | Inactive | Inactive |
| BT-474 | Breast | 3+ | $3.43\times10^6$ | 9.45 (4.2, 15) | Inactive | Inactive | Inactive | Inactive |
| SK-BR-3 | Breast | 3+ | $3.40\times10^6$ | 59.7 (56, 64) | Inactive | Inactive | Inactive | Inactive |
| NCI-N87 | Gastric | 3+ | $2.67\times10^6$ | 10.7 (6.4, 15) | Inactive | Inactive | 76.6 (48, 110) | Inactive |

*95% CI* 95% confidence interval.
[a]HER2 expression by IHC was determined with HercepTest (Dako, Carpinteria, California). Microscopic scoring of tumor cells was interpreted as described in the package insert.
[b] Values are mean from 3 to 6 separate experiments. Values extrapolated from standard curve generated with four bead standards, each with a different Ab binding capacity (ABC), the highest was $5.34\times10^5$ ABC/bead.
[c] Values are mean and 95% CI of 3–7 separate experiments. Percent viability (% viability) of treated cells at maximal effect determined by Best-fit Bottom value derived from the 4-parameter log(inhibitor) vs. response sigmoidal curve model fits using GraphPad Prism 9.2.0 (GraphPad Software, Inc., La Jolla, CA).

**Supplementary Table 4. Percent Complement-dependent Cytotoxicity at Maximal Effect Values of Anti-HER2 Abs in Cancer Cell Lines**

| Cell Line | Cancer Type | HER2 IHC Overall Score[a] | Average HER2 Receptors/Cell[b] | Percent of viable cells remaining following treatment at maximal effect (95% CI)[c] | | | | |
|---|---|---|---|---|---|---|---|---|
| | | | | zanidatamab | trastuzumab | pertuzumab | tras + pert | Negative control |
| JIMT-1 | Breast | 2+ | $5.27x10^5$ | Inactive | Inactive | Inactive | Inactive | Inactive |
| ZR-75-1 | Breast | 2+ | $3.50x10^5$ | Inactive | Inactive | Inactive | Inactive | Inactive |
| MCF7 | Breast | 1+ | $8.25x10^4$ | Inactive | Inactive | Inactive | Inactive | Inactive |
| MDA-MB-175-VII | Breast | 0 | $1.99x10^5$ | Inactive | Inactive | Inactive | Inactive | Inactive |

95% CI = 95% confidence interval.
[a]HER2 expression by IHC was determined with HercepTest (Dako, Carpinteria, California). Microscopic scoring of tumor cells was interpreted as described in the package insert [4]
[b] Values are mean from 3 to 6 separate experiments. Values extrapolated from standard curve generated with four bead standards, each with a different Ab binding capacity (ABC), the highest was $5.34x10^5$ ABC/bead
[c] Data is from *n*=3 (JIMT-1, MCF7, MDA-MB-175-VII) or *n*=2 (ZR-75-1) independent experiments. Percent viability (% viability) of treated cells at maximal effect determined by Best-fit Bottom value derived from the 4-parameter log(inhibitor) vs. response sigmoidal curve model fits using GraphPad Prism 9.2.0 (GraphPad Software, Inc., La Jolla, CA)

Table 1 and Supplementary Table 4 of the paper (doi: 10.1038/s41467-023-37029-3)

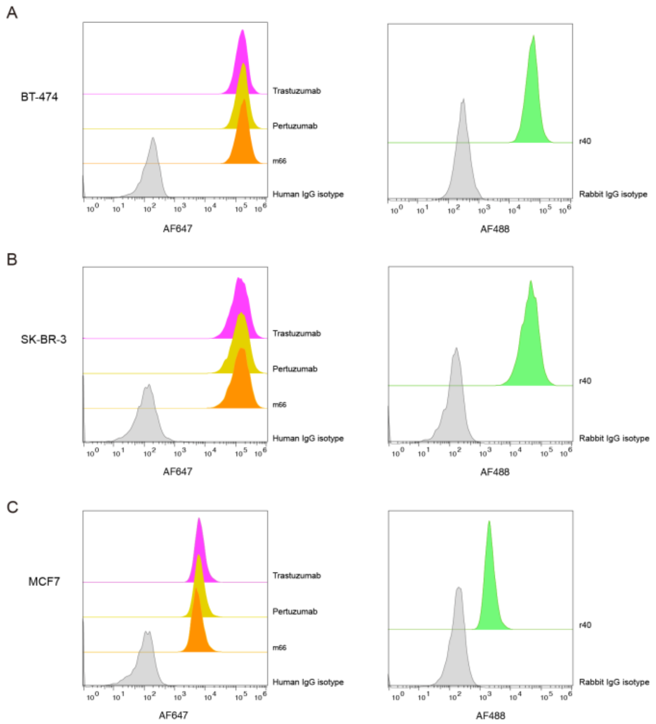

Fig. 5. Binding profiles of trastuzumab, pertuzumab, m66 and r40 to three tumor cell lines expressing HER2.

Further, the MFI of the flow cytometry analysis also revealed significantly lower HER2 protein levels in MCF7 cells compared to both BT-474 and SK-BR-3 cells (Figure 5 in this response letter, same with Figure 3 in the revised manuscript).

e. **What concentration of the tested antibodies did induce the inhibitory effect and what were the $EC_{50}$ s for the inhibition? What criteria did the authors use to compare the inhibitory effect for all 4 antibodies? It looks that the effective concentrations for the tested antibodies were way too high (100 µg/mL equal 670 nM).**

We adjusted antibody concentrations using threefold serial dilutions starting from 100 nM (15 ug/mL) and re-assessed proliferation across three breast cancer cell lines. These results are presented in Figure 2A and 2B of the revised manuscript. And the $EC_{50}$ values were determined by fitting dose-response curves from CCK-8 assays in these three breast cancer cells (Table 1).

| | T | P | m66 | r40 | T+P | T+P+r40 |
|---|---|---|---|---|---|---|
| BT-474 | 5.04 | 2.217 | 2.15 | 2.277 | 1.984 | 1.51 |
| SK-BR-3 | 3.099 | 2.297 | Unstable | 2.66 | 7.899 | 1.585 |
| MCF7 | 0.02602 | 1.041 | 1.436 | 0.00002711 | 1.075 | 1.122 |

Table 1. Antibody $EC_{50}$ values were determined by fitting dose-response curves from CCK-8 assays in breast cancer cells

The $EC_{50}$ s of trastumab, pertuzumab, m66 and r40 with immobilized HER2 ECD protein are 4.837 ng/mL (32.247 pM), 6.954 ng/mL (46.36 pM), 13.59 ng/mL (90.6 pM) and 4.146 ng/mL (27.64 pM) by ELISA analysis (Figure 6).

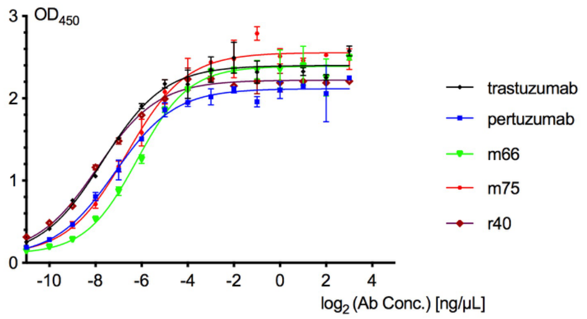


| Antibody | $EC_{50}$ (ng/mL) |
|---|---|
| trastuzumab | 4.837 |
| pertuzumab | 6.954 |
| m66 | 13.59 |
| m75 | 9.973 |
| r40 | 4.146 |

Fig. 6. $EC_{5\ 0}$ of selected antibodies were determined by ELISA analysis.

**f. Why the data presented in a different manner for different cells (Figure 2).**

Figure 2 has been updated in the revised manuscript. We initially assessed the effects of trastuzumab, pertuzumab, mouse monoclonal antibody m66, and six rabbit monoclonal antibodies (1-100 nM) on BT-474 cell proliferation. Combinations including trastuzumab + pertuzumab and triple antibodies (trastuzumab + pertuzumab + r40) were also evaluated. Antibodies and combinations showing inhibitory activity were subsequently validated in SK-BR-3 and MCF7 cells. These results are presented in Figure 2A and 2B of the manuscript.

**g. Why NRG1-independent and NRG1-dependent proliferation experiment was performed only in MCF7 cells?**

In the revised manuscript, the effect of antibodies on cell proliferation under ligand-dependent conditions was not assessed. We assessed antibody-mediated proliferation inhibition under ligand-independent conditions across all three breast cancer cell lines. The r40 antibody demonstrated inhibitory effects comparable to or greater than trastuzumab and pertuzumab, and the inhibitory effect of m66 was weaker than r40.

The triple combination (trastuzumab + pertuzumab + r40) exhibited inhibition equivalent to trastuzumab + pertuzumab in MCF7 cells, while showing superior efficacy at concentrations below 1 nM in both BT-474 and SK-BR-3 cells. These results are presented in Figure 2A and Figure 2B of the manuscript.

3. **HER2 signaling**

   a. **Again, what was the rationale to use one or another cell line in different experiments? The authors either need a good rationale to use one cell liner, or use all three and show the results for all three.**

In the revised manuscript, we assessed the effects of individual antibodies, trastuzumab + pertuzumab dual combination, and triple combination (trastuzumab + pertuzumab + r40) on HER2 signaling pathways across three cell lines under three ligand conditions: ligand-free, EGF-stimulated, and NRG1-stimulated. These results are presented in Figure 4 and Figure 5 of the manuscript.

   b. **What was a rationale for using 50µg/mL of the antibodies? This is unusually high concentration for an antibody, especially for the antibodies with the reported high affinity – in this manuscript, the novel antibodies exhibit sub-nanomolar (r40) and low nanomolar (m66) affinity.**

We tested four antibody concentrations (5, 10, 25, and 50 µg/mL) in MCF7 cells and observed minimal variation in HER2 protein downregulation across

these doses (Figure 4A in the revised manuscript). As concentrations between 10-50 μg/mL are standard in published literatures, we selected 10 μg/mL for subsequent Western blotting experiments. Some of the previously published literatures are listed as follows.

2C4 is Pertuzumab

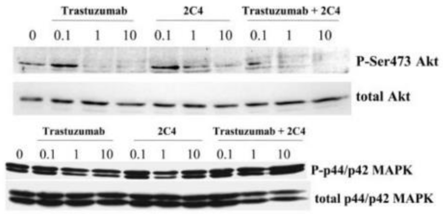


Fig. 4. Trastuzumab and pertuzumab (*2C4*) inhibit Akt signaling. BT474 cells were treated with 0.1, 1, or 10 μg/ml trastuzumab and/or 2C4 for 5 days. Total protein lysates (50 μg) were immunoblotted for phosporylated serine 473 Akt (*P-Ser473 Akt*), total Akt, phosphorylated p44/p42 mitogen-activated protein kinase (*P-p44/p42 MAPK*), and total MAPK (*total p44/p42 MAPK*).

***2004- Cancer Research- The HER- 2- Targeting Antibodies Trastuzumab and Pertuzumab Synergistically Inhibit the Survival of Breast Cancer Cells***

10 ug/mL

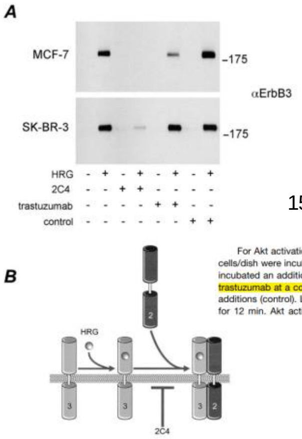


15 ug/mL

For Akt activation, 10 cm dishes seeded the night before with $3 \times 10^6$ cells/dish were incubated for 3 hr in serum-free media. The cells were then incubated an additional hour in serum-free media containing either 2C4 or trastuzumab at a concentration of 100 nM, or in serum-free media without additions (control). Ligand stimulation was carried out using 1 nM rHRGβ1 for 12 min. Akt activity was determined with an assay kit (Cell Signaling

**Figure 1.** 2C4, but not trastuzumab, blocks the association of ErbB2 with ErbB3 in breast cancer cell lines that express normal/low and high ErbB2 levels

**A:** MCF7 (top), which expresses ErbB3 and low or normal levels of ErbB2, and SK-BR-3 (bottom), which expresses ErbB3 and overexpresses ErbB2 at high levels, were treated with HRG or control buffer. ErbB3 was found in ErbB2 immunoprecipitates only when MCF7 cells are treated with the ErbB3 ligand HRG. A similar result is obtained when immunoprecipitates are prepared from the high ErbB2-expressing cell line SK-BR-3. **B:** A model for the proposed mechanism of 2C4 activity.

***2002- Cancer Cell- Targeting ligand- activated ErbB2 signaling inhibits breast and prostate tumor growth***

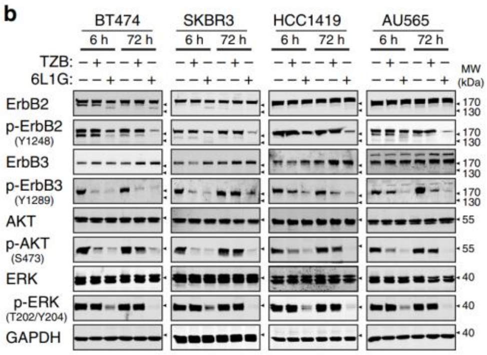


[illegible] 72 h [illegible]

[illegible]

[illegible]

[illegible]

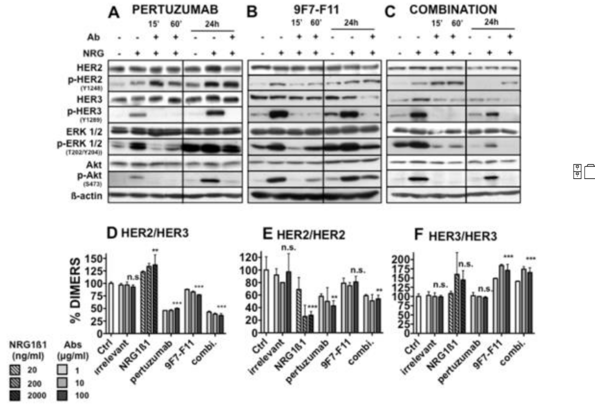


**Figure 5: Effect of pertuzumab and of the anti-HER3 antibody 9F7-F11 on HER2, HER3 and downstream signaling pathways in BxPC-3 cells.** Cells were pre-incubated with pertuzumab [A], 9F7-F11 (B) or both antibodies (C) (50 µg/ml/each antibody for the indicated time) and then with 100 ng/ml NRG1ß1 for 10 minutes. The expression level of phosphorylated and total HER2, HER3, AKT and ERK was then analyzed by western blotting. Effect of pertuzumab and 9F7-F11 on (D) HER2/HER3, (E) HER2/HER2 and (F) HER3/HER3 dimer formation in NIH/3T3 HER2/HER3 cells. $10^5$ cells/well were incubated with increasing concentrations (1 to 100 µg/ml) of antibodies in serum-free medium for 30 minutes. The TR-FRET signal was expressed as Delta F665 (%) and then as dimer percentage (see "Materials and Methods"). Data are the mean ± SEM of 3 experiments performed in triplicate. P, ***<0.001, ** p < 0.01, n.s., not significant.

[illegible]

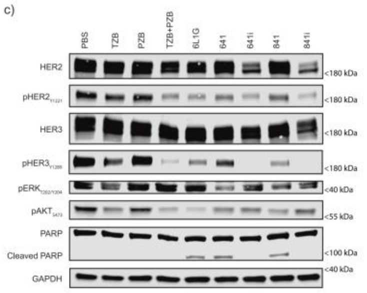


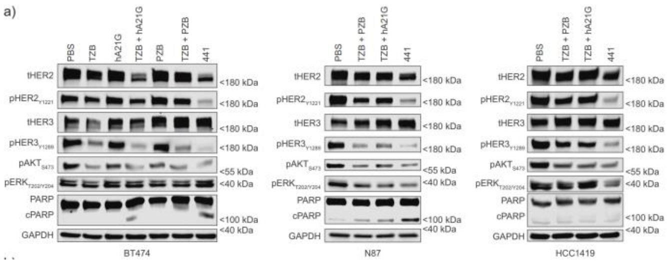


**24 hours** [illegible]

[illegible] **20 nM** [illegible] **24 h** [illegible]

[illegible]

[illegible]

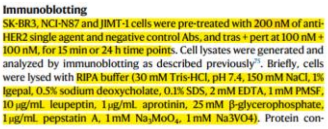

**Immunoblotting**

SK-BR3, NCI-N87 and JIMT-1 cells were pre-treated with 200 nM of anti-HER2 single agent and negative control Abs, and tras + pert at 100 nM + 100 nM, for 15 min or 24 h time points. Cell lysates were generated and analyzed by immunoblotting as described previously[75]. Briefly, cells were lysed with RIPA buffer (30 mM Tris-HCl, pH 7.4, 150 mM NaCl, 1% Igepal, 0.5% sodium deoxycholate, 0.1% SDS, 2 mM EDTA, 1 mM PMSF, 10 μg/mL leupeptin, 1 μg/mL aprotinin, 25 mM β-glycerophosphate, 1 μg/mL pepstatin A, 1 mM $Na_3MoO_4$, 1 mM Na3VO4). Protein con-

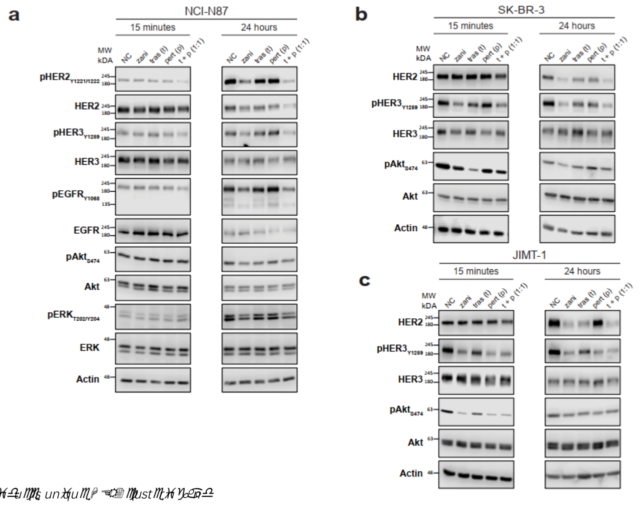


[illegible]

[illegible]

c.  **Western blot images need quantification.**

Thanks for the reviewer's comment. In the revised manuscript, we have updated the Western blot analyses (Figure 4 and Figure 5), band intensity differences were sufficiently clear and interpretable,  so we didn't use quantification software.

d.  **Interestingly, r40 affects the AKT pathway in SK-BR-3 cell line but not in BT-474 (Figure 4). Is it a cell-line dependent effect, or there is a rational reason for it?**

Thanks for the reviewer's comment. We observed that r40 reduced p-AKT (S473) levels in BT-474 cells, though this reduction was less pronounced than in SK-BR-3 cells. In the revised manuscript, we have updated the ligand-independent Western blotting results (Figure 4 in the revised manuscript). The r40 antibody did not reduce pAKT (S473) phosphorylation in MCF7 cells, whereas pAKT (S473) levels were significantly reduced in BT-474 and weakly reduced in SK-BR-3 cells. So we think it is a cell-line dependent effect.

e. **For the ligand-dependent signaling, r40 affected AKT pathway in BT-474, and, to least extent, in SK-BR-3 but not in MCF7. Did the authors consider this difference?**

In the revised manuscript, we stimulated three breast cancer cell lines with EGF and NRG1 ligands separately.

Under EGF stimulation: r40 exhibited the most pronounced reduction of p-AKT (S473) in BT-474 cells. The triple antibody combination (trastuzumab + pertuzumab + r40) reduced p-AKT (S473) more effectively than the dual combination in both BT-474 and SK-BR-3 cells （Figure 5A-5C of the revised manuscript）.

Under NRG1 stimulation: r40 showed weaker activity. The triple combination demonstrated superior efficacy (Figure 5D-5F of the revised manuscript).

These differential responses are attributed to cellular heterogeneity across the different kinds of cells.

f. **Finally, pharmacological inhibitors of the AKT and ERK are needed as controls for these type of the experiments**

We used AKT inhibitor LY294002 and ERK inhibitor U0126 for the kinase assay in MCF7. As shown below, the two inhibitors clearly decreased the levels of p-AKT (S473) and p-ERK (T202/Y204) (Figure 7).

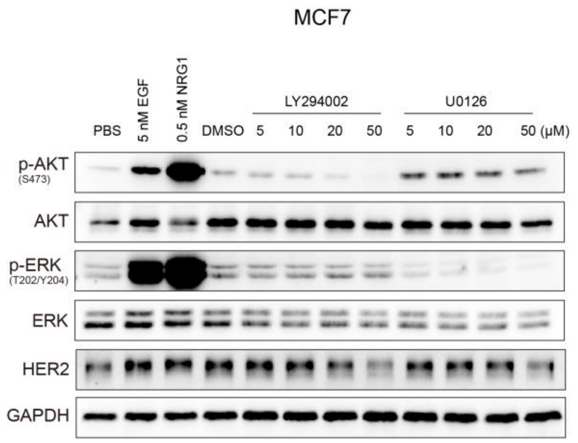


Fig.7. Effects of ligands and small-molecule inhibitors on the HER2 signaling pathway in MCF7 cells

5. Review Comments to the Author

Please use the space provided to explain your answers to the questions above. You may also include additional comments for the author, including concerns about dual publication, research ethics, or publication ethics. (Please upload your review as an attachment if it exceeds 20,000 characters)

Reviewer #1: These authors sought to identify a novel antibody specific to HER2-expressing cancers that could combat treatment resistance and cancer recurrence. They found that r40 could inhibit several key pathways, including cell proliferation, by modulating several cellular pathways such as PI3K/AKT and MAPK.

I found this paper very interesting to read and I congratulate the authors on their novel work. My main comments are around sentence structure and grammatical/spelling errors. I found that the authors would often switch between past and present tense when discussing their results. For clarity and readability, it is important to pick one tense and stick to it throughout. I also found that the authors sometimes abbreviated terms with the acronym coming before the term. For example:

"The EGFR (human epidermal growth factor receptor) family comprises four members..."

However, this should read:

"The human epidermal growth factor receptor (EGFR) family comprises four members..."

Other times the authors correctly put the acronym in brackets afterwards. Please ensure this is consist across the manuscript. I have attached my revisions in a word doc. Again, I would like to congratulate the authors on a novel study that address an urgent gap in clinical care for patients with HER2 cancers.

We thank the reviewer for evaluating our manuscript and the constructive comments that are very helpful for improvement of the manuscript. We have clearly checked our manuscript and the placement of abbreviations relative to full terms has been standardized. And also we made several changes on the tense, as shown in page 5 of the revised manuscript "indicating that the binding affinity of m66 was weaker than that of pertuzumab", "is" has been changed to "was"; "The superimposed structure of HER2-HER3-NRG1 (PDB: 7MN5) and HER2-r40-trastuzumab-pertuzumab structure showed that the epitope of antibody r40 was opposite to the dimerization interfaces of HER2" in page 10 of the revised manuscript, "indicates" has been corrected to "showed" and "is" has been corrected to "was".

Reviewer #2: This manuscript demonstrated the selection of mouse antibody m66 and rabbit antibody r40, which target distinct sites on HER2 that are different from those bound by trastuzumab and pertuzumab. It also examined their effects on inhibiting HER2-dependent signaling pathways.

Unfortunately, this manuscript does not include data from cancer cell proliferation inhibition assays using these antibodies in combination. As a result, the insights gained from the structural analysis of the antibody-HER2 complexes are not fully utilized, diminishing the impact of the report.

We thank the reviewer for evaluating our manuscript and the constructive comments that are very helpful for improvement of the manuscript. We have supplemented the effects of the triple-antibody combination on cell proliferation and the HER2 signaling pathway in Figure 2，Figure 4 and Figure 5 of our revised manuscript.

Additionally, while the experiments were conducted properly and yielded new findings, the results were presented in a manner that could lead to erroneous conclusions. Consequently, it has been determined that, in its current form, this manuscript is insufficient for publication.

Throughout the manuscript, the initial citations of figures are not appropriately placed. Figures should be referenced within specific explanatory contexts. For example, Fig 1A should be cited in the sentence: "As expected, each non-His-tagged IgG significantly blocked...".
Similar citation issues appear throughout the manuscript.
We thank the reviewer for evaluating our manuscript and the constructive comments that are very helpful for improvement of the manuscript. We have clearly checked our manuscript and made several changes on this. "As expected, each non-His-tagged IgG significantly blocked the binding of its own His-tagged IgG (Fig 1A)".

There are numerous parts of the text that are insufficiently explained. The authors should seek feedback from colleagues by having them review the manuscript.
Below are some examples:

- In the Abstract and Introduction sections, both r40 and m66 should be mentioned, along with a clarification that they are monoclonal antibodies derived from rabbit and mouse, respectively.

We have changed the sentence in the Abstract, "Here, we describe a novel monoclonal antibody targeting HER2, termed r40 " to " Here, we describe two novel HER2-targeting monoclonal antibodies, m66 (mouse-derived) and r40 (rabbit-derived), which inhibit breast cancer cell proliferation in vitro" in the revised manuscript.

- The sentence, "The binding kinetics of trastuzumab, pertuzumab…" should specify the binding target, possibly HER2.

We have changed the sentence: "The binding kinetics of trastuzumab, pertuzumab, m66 and r40 were evaluated by surface plasmon resonance (SPR)" to "The binding kinetics of trastuzumab, pertuzumab, m66 and r40 with HER2 were evaluated by surface plasmon resonance Imaging (SPRi)" in the page 7 in the reviesed manuscript.

- A reference for PDB entry #7MN5 should be provided.

We have provided the reference (2021, Nature) for PDB #7MN5 in the page 10 in the reviesed manuscript.

- Figure legends should provide more specific details to allow the experiments to be understood without referring to the Materials and methods section.

We have provided more detailed figure legends in the revised manuscript, for example the legend of Fig 1B is as follows, "Saturating concentrations of trastuzumab and pertuzumab for binding to HER2 on live MCF7 cells were determined. MCF7 cells were incubated with trastuzumab and pertuzumab (0-400 nM) for 30 min, then stained with AF647-conjugated goat anti-human secondary antibody for flow cytometry analysis." The legend of Fig 4A is as follows, "Immunoblots assessing HER2 signaling upon treatment with 5, 10, 25, 50 μg/mL (33.3, 66.7, 166.7, 333.3 nM) antibodies for 24 h in MCF7. Cell lysates were immunoblotted to detect p-AKT (Ser473), total AKT, p-ERK (T202/Y204), total ERK, HER2, GAPDH."

This manuscript requires extensive proofreading, as it contains numerous spelling mistakes and typographical errors. Ensure that the co-authors thoroughly review the content.

We thank the reviewer for evaluating our manuscript and the constructive comments that are very helpful for improvement of the manuscript. We have clearly checked our manuscript and made several changes on this.

For example, in the sentence: "The final structural models were built with the HER2-trastuzumab-...", the reference for PDB entry #60GE may be [Ref. 10] rather than [Ref. 25].

Thanks for the reviewer's comment, we have changed the reference in the revised manuscript.

About the Figures:

Fig 2C: There is no statistically significant difference between r40 and trastuzumab in the NRG1 ligand treatment of MCF7 cells. Therefore, statements such as "Antibody r40 shows a more potent effect than trastuzumab..." should be avoided to prevent misinterpretation.

Indeed, the observed differences were not sufficiently pronounced. Consequently, we have discontinued this figure in the revised manuscript. Instead of reassessing proliferation under ligand-dependent conditions, we evaluated cell viability across three cell lines following 4 d treatment with antibody concentrations ranging from 0 to 100 nM in Figure 2A and Figure 2B of the revised manuscript.

Fig 3: The horizontal axes should be aligned to facilitate vertical comparisons, as demonstrated in Fig 1B. Additionally, the peak intensity of r40 in BT-474 appears to be very low. What could be the possible explanation?

We thank the reviewer for this query. The revised manuscript contains an updated version of this figure (see revised Figure 3). As the variable regions of m66, trastuzumab, and pertuzumab were all cloned into a human IgG1 backbone, these antibodies - along with the human IgG isotype control - were detected using anti-human fluorescent secondary antibodies. Conversely, r40 was constructed with a rabbit IgG backbone and was therefore detected with anti-rabbit secondary antibodies alongside its rabbit IgG isotype control.

Figs 4 and 5: Blots should be digitally quantified and, if possible, represented as graphs. Of course, images of the blots should be incorporated into the graphs. Ideally, statistical analysis should also be conducted, and the discussion should be based on those results.

Thanks for the reviewer's comment, In the revised manuscript, we have updated the Western blot analyses (see Figure 4 and 5), band intensity differences were sufficiently clear and interpretable, so we didn’t use quantification software.

Fig 6: It would be better to adjust the display order of the figures. Following the sequence in the Results section, the results for m66 and r40 should be presented separately for each, in the order of trimer, tetrameric complex, and superimposed structure.

Thanks for the reviewer's comment, we have sepetated the results of m66 and r40 in Figure 6 in the revised manuscript.

Reviewer #3: A nice in vitro study of selecting a new antibodies to HER2. Study is methodologically sound, data are well presented and manuscript as a whole is written in logical manner. Cryo EM part of study is especially commendable.

Thanks for the reviewer's comment.
