# Supplementary material for: Inhibition of HER2 signaling and breast cancer cell growth with a novel antibody targeting HER2 ECD III/IV
Source: PLoS One. 2026 Jan 15;21(1):e0338127. doi: 10.1371/journal.pone.0338127 (PMC12806845; doi:10.1371/journal.pone.0338127)
Supplement: Attachment — Submitted filename: PONE-D-25-13465_rev.docx [file pone.0338127.s010.docx]

PLOS ONE

Inhibition of HER2 signaling and breast cancer cell growth with a novel antibody targeting HER2 ECD III/IV

--Manuscript Draft--

| Manuscript Number: | PONE-D-25-13465 |
| --- | --- |
| Article Type: | Research Article |
| Full Title: | Inhibition of HER2 signaling and breast cancer cell growth with a novel antibody targeting HER2 ECD III/IV |
| Short Title: | Inhibition of breast cancer cell growth with a novel antibody |
| Corresponding Author: | Chunchun Liu  Fudan University Shanghai Cancer Center Shanghai, CHINA |
| Keywords: | HER2, breast cancer, antibody, cryo-EM |
| Abstract: | HER2 (Human epidermal growth factor receptor 2), a ligand-independent tyrosine kinase receptor of the EGFR family, functions as a key oncogenic factor in breast, gastric and several other solid malignancies. Despite the huge survival benefits from anti-HER2 treatment, particularly in HER2 overexpression breast cancer and gastric cancer, the emergence of treatment resistance and cancer recurrence persists as a significant challenge. Thus, the creation of innovative antibodies exhibiting complementary effects to the current anti-HER2 therapies could offer clinical benefits. Here, we describe a novel monoclonal antibody targeting HER2, termed r40, that demonstrates inhibitory activity on the proliferation of breast cancer cells. Antibody r40 suppresses the PI3K/AKT and MAPK signaling pathways via decreasing the level of HER2 protein. Furthermore, we report the cryo-EM structure determination of HER2- trastuzumab-pertuzumab-r40 tetrameric complex to overall 3.1 Å resolution. The structure indicates that the epitope of r40 is mapped within the HRE2 ECD III/IV region, unlike the epitopes of trastuzumab and pertuzumab. The findings of this study highlight that r40 may be considered as a promising candicate for the therapy of breast cancer with HER2 overexpression. |
| Order of Authors: | Chunchun Liu |
|  | Yulei Ren |
|  | Xuan Luo |
|  | Peiyun Zhou |
|  | Xiangjuan Du |
|  | Qianmin Wang |
|  | Xue Han |
|  | Yanhui Xu |
|  | Ping Wang |
|  | Dan Zhao |
|  | Huirong Yang |
| Additional Information: |  |
| Question | Response |
| Financial Disclosure  Enter a financial disclosure statement that describes the sources of funding for the work included in this submission. Review the [submission guidelines](http://journals.plos.org/plosone/s/submission-guidelines#loc-financial-disclosure-statement) for detailed | Yes |

*Powered by Editorial Manager® and ProduXion Manager® from Aries Systems Corporation*

| requirements. View published research articles from [PLOS ONE](https://journals.plos.org/plosone/) for specific examples.  This statement is required for submission and will appear in the published article if the submission is accepted. Please make sure it is accurate. |  |
| --- | --- |
| Funded studies  Enter a statement with the following details:   - Initials of the authors who received each award - Grant numbers awarded to each author - The full name of each funder - URL of each funder website - Did the sponsors or funders play any role in the study design, data collection and analysis, decision to publish, or preparation of the manuscript? |  |
| Did you receive funding for this work? |  |
| Please add funding details.  as follow-up to "Financial Disclosure  Enter a financial disclosure statement that describes the sources of funding for the work included in this submission. Review the [submission guidelines](http://journals.plos.org/plosone/s/submission-guidelines#loc-financial-disclosure-statement) for detailed requirements. View published research articles from [PLOS ONE](https://journals.plos.org/plosone/) for specific examples.  This statement is required for submission and will appear in the published article if the submission is accepted. Please make sure it is accurate. | Shanghai Natural Science Foundation of China (21ZR1407900) |

*Powered by Editorial Manager® and ProduXion Manager® from Aries Systems Corporation*

| Funded studies  Enter a statement with the following details:   - Initials of the authors who received each award - Grant numbers awarded to each author - The full name of each funder - URL of each funder website - Did the sponsors or funders play any role in the study design, data collection and analysis, decision to publish, or preparation of the manuscript? |  |
| --- | --- |
| Did you receive funding for this work?" |  |
| Please select the country of your main research funder (please select carefully as in some cases this is used in fee calculation).  as follow-up to "Financial Disclosure  Enter a financial disclosure statement that describes the sources of funding for the work included in this submission. Review the [submission guidelines](http://journals.plos.org/plosone/s/submission-guidelines#loc-financial-disclosure-statement) for detailed requirements. View published research articles from [PLOS ONE](https://journals.plos.org/plosone/) for specific examples.  This statement is required for submission and will appear in the published article if the submission is accepted. Please make sure it is accurate. | CHINA - CN |
| Funded studies  Enter a statement with the following details:   - Initials of the authors who received each award - Grant numbers awarded to each author - The full name of each funder - URL of each funder website - Did the sponsors or funders play any role in the study design, data collection and analysis, decision to publish, or preparation of the manuscript? |  |
| Did you receive funding for this work?" |  |
| Competing Interests | The authors have declared that no competing interests exist. |

*Powered by Editorial Manager® and ProduXion Manager® from Aries Systems Corporation*

| Use the instructions below to enter a competing interest statement for this submission. On behalf of all authors, disclose any [competing interests](http://journals.plos.org/plosone/s/competing-interests) that could be perceived to bias this work—acknowledging all financial support and any other relevant financial or non- financial competing interests.  This statement is required for submission and will appear in the published article if the submission is accepted. Please make sure it is accurate and that any funding sources listed in your Funding Information later in the submission form are also declared in your Financial Disclosure statement.  View published research articles from  [PLOS ONE](http://journals.plos.org/plosone/) for specific examples. |  |
| --- | --- |
| NO authors have competing interests  Enter: The authors have declared that no competing interests exist.  Authors with competing interests  Enter competing interest details beginning with this statement:  I have read the journal's policy and the authors of this manuscript have the following competing interests: [insert competing interests here] |  |
| * typeset |  |
| Ethics Statement  Enter an ethics statement for this submission. This statement is required if the study involved:   - Human participants - Human specimens or tissue - Vertebrate animals or cephalopods - Vertebrate embryos or tissues | N/A |

*Powered by Editorial Manager® and ProduXion Manager® from Aries Systems Corporation*

| - Field research   Write "N/A" if the submission does not require an ethics statement.  General guidance is provided below. Consult the [submission guidelines](http://journals.plos.org/plosone/s/submission-guidelines#loc-guidelines-for-specific-study-types) for detailed instructions. Make sure that all information entered here is included in the Methods section of the manuscript. |  |
| --- | --- |

*Powered by Editorial Manager® and ProduXion Manager® from Aries Systems Corporation*

| Format for specific study types  Human Subject Research (involving human participants and/or tissue)   - Give the name of the institutional review board or ethics committee that approved the study - Include the approval number and/or a statement indicating approval of this research - Indicate the form of consent obtained (written/oral) or the reason that consent was not obtained (e.g. the data were analyzed anonymously)   Animal Research (involving vertebrate animals, embryos or tissues)   - Provide the name of the Institutional Animal   Care and Use Committee (IACUC) or other relevant ethics board that reviewed the study protocol, and indicate whether they approved this research or granted a formal waiver of ethical approval   - Include an approval number if one was obtained - If the study involved non-human primates, add additional details about animal welfare and steps taken to ameliorate suffering - If anesthesia, euthanasia, or any kind of animal sacrifice is part of the study, include briefly which substances and/or methods were applied   Field Research  Include the following details if this study involves the collection of plant, animal, or other materials from a natural setting:   - Field permit number - Name of the institution or relevant body that granted permission |  |
| --- | --- |
|  |  |
| Data Availability  Authors are required to make all data underlying the findings described fully available, without restriction, and from the time of publication. PLOS allows rare exceptions to address legal and ethical concerns. See the [PLOS Data Policy](http://journals.plos.org/plosone/s/data-availability) and [FAQ](http://journals.plos.org/plosone/s/data-availability#loc-faqs-for-data-policy) for detailed information. | Yes - all data are fully available without restriction |

*Powered by Editorial Manager® and ProduXion Manager® from Aries Systems Corporation*

| A Data Availability Statement describing where the data can be found is required at submission. Your answers to this question constitute the Data Availability Statement and will be published in the article, if accepted. |  |
| --- | --- |
| Important: Stating ‘data available on request from the author’ is not sufficient. If your data are only available upon request, select ‘No’ for the first question and explain your exceptional situation in the text box. |  |
| Do the authors confirm that all data underlying the findings described in their manuscript are fully available without restriction? |  |
| Describe where the data may be found in full sentences. If you are copying our sample text, replace any instances of XXX with the appropriate details.   - If the data are held or will be held in a public repository, include URLs, accession numbers or DOIs. If this information will only be available after acceptance, indicate this by ticking the box below. For example: All XXX files are available from the XXX database (accession number(s) XXX, XXX.). - If the data are all contained within the manuscript and/or Supporting Information files, enter the following: All relevant data are within the manuscript and its Supporting Information files. - If neither of these applies but you are able to provide details of access elsewhere, with or without limitations, please do so. For example:   Data cannot be shared publicly because of [XXX]. Data are available from the XXX Institutional Data Access / Ethics Committee (contact via XXX) for researchers who meet the criteria for access to confidential data.  The data underlying the results presented in the study are available from (include the name of the third party | All relevant data are within the manuscript and its Supporting Information files. |

*Powered by Editorial Manager® and ProduXion Manager® from Aries Systems Corporation*

| and contact information or URL).   - This text is appropriate if the data are owned by a third party and authors do not have permission to share the data.   * typeset |  |
| --- | --- |
| Additional data availability information: |  |

*Powered by Editorial Manager® and ProduXion Manager® from Aries Systems Corporation*

Manuscript	[Click here to access/download;Manuscript;Manuscript.docx](https://www2.cloud.editorialmanager.com/pone/download.aspx?id=39670254&guid=930206bd-5a2b-4c2d-9444-8202f28543ca&scheme=1)[<https://www2.cloud.editorialmanager.com/pone/download.aspx?id=39670254&guid=930206bd-5a2b-4c2d-9444-8202f28543ca&scheme=1>](https://www2.cloud.editorialmanager.com/pone/download.aspx?id=39670254&guid=930206bd-5a2b-4c2d-9444-8202f28543ca&scheme=1)

# Inhibition of HER2 signaling and breast cancer cell growth with a novel antibody targeting HER2 ECD III/IV

Chunchun Liu 1, ¶, Yulei Ren 1, ¶, Xuan Luo 3, 4, ¶, Peiyun Zhou ^1^, Xiangjuan Du ^1^, Qianmin Wang ^1^,

Xue Han ^1^, Yanhui Xu 1, ^2^, Ping Wang ^6^, Dan Zhao 1, 5, ^*^, Huirong Yang 1, 2, *

1 Fudan University Shanghai Cancer Center, Institutes of Biomedical Sciences, Shanghai Key Laboratory of Radiation Oncology, and Shanghai Key Laboratory of Medical Epigenetics, Shanghai Medical College of Fudan University, Shanghai, China.

2 The International Co-laboratory of Medical Epigenetics and Metabolism, Ministry of Science and Technology, China, Department of Systems Biology for Medicine, School of Basic Medical Sciences, Shanghai Medical College of Fudan University, Shanghai, China.

3 Key Laboratory of Breast Cancer in Shanghai, Department of Breast Surgery, Fudan University Shanghai Cancer Center, Shanghai Medical College, Fudan University, Shanghai, China

4 Department of Oncology, Shanghai Medical College, Fudan University, Shanghai, China

5 School of Life Sciences, Greater Bay Area Institute of Precision Medicine (Guangzhou), Fudan University, Guangzhou, China

6 Molinsights Biotechnologies, Guangdong Medical Valley Life Science Park, Guangzhou, China.

* Corresponding author

E-mail: [danzhao5278@foxmail.com,](mailto:danzhao5278@foxmail.com) [yanghr@fudan.edu.cn](mailto:yanghr@fudan.edu.cn)

¶These authors contributed equally to this work.

# Abstract

~~HER2 (Human epidermal growth factor receptor 2),~~ Human epidermal growth factor 2 (HER2), a ligand-independent tyrosine kinase receptor of the EGFR family, functions as a key oncogenic factor in breast, gastric and several other solid malignancies. Despite the huge survival benefits from anti-HER2 treatment, particularly in ~~HER2 overexpression~~ breast cancer and gastric cancer that overexpress HER2, the emergence of treatment resistance and cancer recurrence persists as a significant challenge. Thus, the creation of innovative antibodies exhibiting complementary effects to the current anti-HER2 therapies could offer clinical benefits. Here, we describe a novel monoclonal antibody targeting HER2, termed r40, that demonstrates inhibitory activity on the proliferation of breast cancer cells. Antibody r40 suppresses the PI3K/AKT and MAPK signaling pathways via decreasing the level of HER2 protein. Furthermore, we report the cryo-EM structure determination of HER2-trastuzumab-pertuzumab-r40 tetrameric complex to overall 3.1 Å resolution. The structure indicates that the epitope of r40 is mapped within the HRE2 ECD III/IV region, unlike the epitopes of trastuzumab and pertuzumab. The findings of this study highlight that r40 may be considered as a promising ~~candicate~~ candidate for the therapy of breast cancer with HER2 overexpression.

# Introduction

The ~~EGFR (human epidermal growth factor receptor)~~ human epidermal growth factor receptor (EGFR) family comprises four members: EGFR (ERBB1, HER1), HER2 (ERBB2), HER3 (ERBB3) and HER4 (ERBB4). ~~All~~

~~these four~~ All four of these HER proteins are ~~considered as~~ oncogenic drivers, with EGFR and HER2 highly associated with tumorigenesis. HER2, characterized as a single-pass type I transmembrane protein, ~~is consisted~~ consists of four extracellular domains (ECD I-IV), a transmembrane domain and an intracellular tyrosine kinase domain from N-to-C terminus. Unlike other EGFR family members, HER2 has constitutive activity~~, which~~ that can initiate signaling pathways without a bound activating ligand[1, 2]. Overexpression of HER2 results in ligand-independent homodimerization and ligand-dependent heterodimerization of EGFR/HER2, HER2/HER3, HER2/HER4, causing HER2 phosphorylation, specifically activating the PI3K/AKT and MAPK signaling pathways, and triggering cell division and growth[1, 3].

Current clinical practice guidelines incorporate various anti-HER2 biologics, ranging from monoclonal antibodies to advanced antibody-drug conjugates (ADC) platforms, for managing cancers with amplified HER2 expression. Trastuzumab (Herceptin^®^) was approved for the therapeutic treatment of breast cancer and metastatic gastric cancer in patients with HER2 overexpression in 1998 and 2010, respectively. Trastuzumab binds to ECD IV of HER2~~, which~~ and triggers the internalization and degradation of HER2~~, causing~~. This results in the inhibition of downstream signaling pathways and the elimination of cytotoxic effector T cells via ~~ADCC (antibody-dependent cell-mediated cytotoxicity~~) antibody-dependent cell-mediated cytotoxicity (ADCC) mechanisms[4-6]. Combined with chemotherapy, trastuzumab has demonstrated significant therapeutic benefits in the treatment of HER2-driven breast malignancies. However, therapeutic resistance and disease relapse inevitably occurs after trastuzumab treatment[7]. Pertuzumab (Perjeta^®^) is the second approved anti- HER2 monoclonal antibody (mAb) drug that binds to ECD II of HER2[8, 9]. The binding epitopes of trastuzumab and pertuzumab are both situated at the dimerization interface of HER2[10]. Combinational use of trastuzumab and pertuzumab has been evaluated to exhibit enhanced antitumor effect for HER2 overexpressing breast cancer[11-13].

Other anti-HER2 agents, including small molecular drugs (lapatinib, neratinib), and ADC-based drugs (trastuzumab emtansine, trastuzumab deruxtecan) have been approved for HER2-overexpression breast cancers[14-18]. Trastuzumab and

trastuzumab deruxtecan (T-Dxd) are also approved for the therapy of HER2-positive gastric cancers[19-21]. Moreover, metastatic HER2-driven ~~maglignancies~~ malignancies inevitably develop refractoriness to the current approved HER2 therapies, leading to disease progression over time[22].

The observed synergistic anticancer effect of trastuzumab and pertuzumab targeting topographically distinct HER2 epitopes has catalyzed intensive exploration of novel antibodies recognizing new epitopes[23]. The combination of three anti-HER2 antibodies with non-overlapping epitopes showed higher antitumor efficacy than trastuzumab and pertuzumab combination[24]. Also, the combination of a monoclonal antibody targeting HER2 ECD III with trastuzumab and pertuzumab provided blockade of HER2 heterodimerization and cell signaling[25]. Although neither ECD I nor III is directly involved in HER2 dimerization, ECD I is critical for forming a binding pocket for the dimerization arm of other EGFR family members. ECD III, situated in the center of the whole extracellular domain, serves as a pivotal connector between ECD II and ECD IV, and ~~also~~ establishes contact~~s~~ with ECD I. Antibodies targeting ECD I and III can also ~~possess the potential to~~ modulate the conformational dynamics of the HER2 receptor, which in turn could impede the activation cascade of HER2 signaling[23].

In this study, we report a novel HER2 ECD III/IV specific antibody termed r40 by cryo- electron microscopy (cryo-EM) ~~study, which~~ that can suppress the growth of breast cancer cells by inhibiting ~~via inhibition of~~ the PI3K/AKT and MAPK signaling pathways. ~~Furhermore,~~ Furthermore, we demonstrate that r40 blocks ~~the~~ HER2 signaling through decreasing the level of HER2 protein.

# Results

## Discovery of antibodies targeting HER2 ECD

HER2 ECD specific antibodies were obtained by single cell VDJ sequencing (scVDJ- seq) from the spleens of ~~mouse~~ mice and rabbits immunized with HER2 ECD proteins (S1A Fig). The antibodies selected from B cells were synthesized and expressed *in vitro* and assessed for antigen binding affinity by ELISA and pull-down assays (data not shown). Finally, we ~~got~~ acquired two mouse antibodies (termed m66, m75) and thirty-eight rabbit antibodies among r1-r40 specifically targeting HER2 ECD.

## Antibody m66 competes with pertuzumab for binding to HER2

To characterize the epitopes of the two mouse antibodies, we purified trastuzumab, pertuzumab, m66, and m75 in two human IgG1 formats, both His-tagged and non-His- tagged forms (S1B Fig). Blocking ELISA was performed to assess the binding epitopes by incubating immobilized HER2 with supersaturating non-His-tagged IgGs followed by detecting the binding of His-tagged IgGs. As expected, each non-His-tagged IgG significantly blocked the binding of its own His-tagged IgG. In addition, supersaturation of m66 and m75 did not influence the binding of trastuzumab, indicating that m66 and m75 target epitopes distinct from that of trastuzumab. Antibody m66 binding was blocked by pertuzumab but not vice versa, indicating that the binding affinity of m66 ~~is~~ was weaker than that of pertuzumab. Antibody m75 blocked pertuzumab and pertuzumab blocked m75. These results indicate that m66 and m75 bind to distinct epitopes from that of trastuzumab, while they likely bind to epitopes overlapping with pertuzumab (Fig 1A). As a result, m66 antibody was selected for further investigation.

## Antibody r40 binds to a different eptiode from that of trastuzumab and pertuzumab

To characterize epitopes of rabbit antibodies, we first blocked HER2 with supersaturating trastuzumab and pertuzumab, then incubated cells with rabbit antibodies. Flow cytometry assay was conducted to determine whether rabbit antibodies could bind simultaneously. Primarily, we tested the binding ability of thirty-

eight rabbit antibodies, among which r22, r30, r35 ~~couldn’t~~ could not recognize cell surface HER2 (Fig 1B). When saturated with trastuzumab and pertuzumab, the mean fluorescence intensity of a rabbit antibody ~~is~~ was nearly identical to that of the control cells solely incubated with the rabbit mAb~~, which~~ . This suggests that the rabbit antibody can bind to HER2 with trastuzumab and pertuzumab simultaneously. Thus, we suspected that the six rabbit antibodies (r3, r4, r5, r29, r36 and r40) ~~might~~ may recognize epitopes distinct from that of trastuzumab and pertuzumab (Fig 1C, 1D).

## Antibody r40 inhibits breast cancer cell proliferation

~~We next~~ Next, we tested whether these six rabbit antibodies and m66 possess anti-proliferative activities on HER2-overexpressing breast cancer cell line BT-474. Without ligand stimulation, trastuzumab demonstrated more potent suppression of ~~shows a more potent effect in suppressing~~ BT-474 cell growth than pertuzumab. Among the tested antibodies, r40 exhibited an anti-proliferative effect comparable to pertuzumab in BT-474 cells, while the other five rabbit antibodies and mouse m66 showed very weak or undetectable effect on BT-474 cell proliferation (Fig 2A). Thus, r40 and m66 were chosen for further study. Another breast cancer cell line, SK-BR-3 with HER2 overexpression, was also used in the cell proliferation assay. Pertuzumab ~~was shown to~~ inhibited SK-BR-3 cell proliferation more effectively than trastuzumab. Antibody r40 ~~exhibits~~ exhibited ~~a far superior inhibitory effect~~ superior inhibition ~~than that of~~ compared to trastuzumab, while m66 ~~has~~ had an inhibitory effect that was ~~a~~ comparable ~~inhibition effect with~~ to trastuzumab (Fig 2B).

With or without the activation of the NRG1 ligand, both m66 and r40 antibodies significantly inhibited cell proliferation of MCF7 with low HER2 expression. The inhibitory effect of r40 was higher than that of pertuzumab in the absence of NRG1. Antibody r40 ~~shows~~ demonstrated a more potent inhibitory effect than trastuzumab ~~in inhibiting~~ on MCF7 cell proliferation with the activation of NRG1 ligand (Fig 2C).

The binding kinetics of trastuzumab, pertuzumab, m66 and r40 were evaluated by surface plasmon resonance (SPR), which showed high affinity for r40 (*KD* = 0.279 nM) and trastuzumab (*KD* = 0.283 nM), slightly lower binding affinity for pertuzumab (*KD*

= 2.3 nM), and modest affinity for m66 (*KD* = 86.4 nM) (Fig 2D).

## Antibody r40 and m66 could bind and detect cell-surface HER2 of breast cancer

**cells**

Next, we tested whether the rabbit antibodies could recognize the endogenous HER2 protein of the breast cancer cells via flow cytometry. Both m66 and r40 ~~could~~ detected similar levels of the endogenous HER2 proteins as trastuzumab and pertuzumab with regards to mean fluorescence intensity (Fig 3).

## Antibody r40 effectively blocks HER2-dependent signaling pathways

PI3K/AKT and MAPK are the main signaling pathways downstream of the EGFR family[1, 23]. Both trastuzumab and pertuzumab have been reported to inhibit ~~the~~ these two signaling pathways in the breast cancer cells[12, 26-28]. We evaluated some key downstream signaling molecules level upon treatment with antibodies for 6 h or 24 h. ~~With trastuzumab or r40 treatment,~~ There was ~~there is~~ a reduction in pAKT (S473) level in BT- 474 and SK-BR-3 cells when treated with trastuzumab or r40~~, whereas.~~ Contrastingly, both pertuzumab and m66 had weak or undetectable effects on pAKT (S473) level. In both BT-474 and SK-BR-3 cells, 24 h treatment with either trastuzumab or r40 reduced the protein level of HER2. The combinatory use of trastuzumab and pertuzumab markedly decreased pAKT (S473) and HER2 protein levels, which is consistent with their synergistic efficiency in clinical use (Fig 4A, 4B). In conclusion, without the activation of ligand, trastuzumab demonstrates greater efficacy than pertuzumab in disrupting the constitutive HER2 homodimer and heterodimer in the HER2-overexpressing cell lines.

Next, we measured the EGF-induced EGFR/HER2 heterodimerization and downstream signaling pretreated with antibodies. Upon EGF ligand activation, phosphorylation level of AKT and ERK increased largely compared to the control cells. Antibody r40 exhibited a more potent ability to downregulate pAKT (S473), pERK, and HER2 than pertuzumab in BT-474 and SK-BR-3 cells. For MCF7, m66 and r40 also exhibited pAKT inhibition effect, and trastuzumab and r40 ~~antibody treatment~~ ~~obviously~~ decreased HER2 protein level (Fig 5A). Furthermore, we investigated the effect of antibodies on NRG1-induced HER2/HER3 heterodimerization and downstream signaling in MCF7 cells. Both 6 h and 24 h treatment with pertuzumab completely blocked NRG1 activation, and r40 exhibited equal activity with trastuzumab in reducing the levels of pAKT (S473) and pERK compared to the control

NRG1 activated cells. Both 6 h and 24 h treatment with trastuzumab or r40 reduced HER2 levels, and after 24 h treatment with pertuzumab or m66, HER2 levels were slightly reduced (Fig 5B).

Taken together, in BT-474 and SK-BR-3 cells with overexpressing HER2, r40 ~~shows~~ showed a considerable signaling reduction effect in both EGF-dependent and EGF- independent manners. In low HER2-expressing MCF7 cells, r40 treatment reduced HER2, pAKT, and pERK in both EGF and NRG1-dependent manners.

## Complex assembly and structure determination

For the purposes of structural elucidation, antibodies initially in the IgG format were converted to the ~~Fab (fragment antigen-binding)~~ fragment antigen binding (Fab) format (S1B Fig, S1C Fig). To obtain the trimer comprising HER2, m66 and trastuzumab, size exclusion chromatography (SEC) was conducted to obtain the binary complex of HER2 and the m66 Fab. As indicated by the SEC profiles, HER2 protein fully engaged with m66 Fab to form a binary complex, with an additional peak corresponding to the unbound m66 Fab (S2A Fig). The peak of HER2-m66 binary complex was shifted forward with the addition of the trastuzumab Fab, indicating that HER2, m66 and trastuzumab formed a ternary complex (S2B-S2D Fig).

To obtain the tetrameric complex containing HER2, r40, trastuzumab, and pertuzumab, we first purified the binary complex containing HER2 and r40, followed by the addition of trastuzumab (S3A Fig, S3B Fig). The detectable shift of the SEC peak was observed with the addition of the pertuzumab Fab, indicating the presence of the larger tetrameric complex (S3C-S3E Fig).

Single-particle cryo-EM reconstruction was used to determine the structures of the two HER2-antibodies complexes (S4 Fig, S5 Fig). The cryo-EM maps of HER2-m66- trastuzumab and HER2-r40-trastuzumab-pertuzumab were refined to overall resolutions of 3.2 Å and 3.1 Å, respectively. The final structural models were built with the HER2-trastuzumab-pertuzumab structure (PDB: 6OGE)[25] as a template and Alphafold2 structure prediction. For the HER2-m66-trastuzumab complex, the three components could be clearly identified in the cryo-EM map, and a final high-resolution model was built and refined (Fig 6A). Trastuzumab binds ECD IV and m66 epitope

located in ECD I of HER2. We compared HER2-trastuzumab-pertuzumab (PDB: 6OGE) and HER2-m66-trastuzumab we determined with HER2 superimposed (Fig 6C). The binding site of pertuzumab is located at the dimerization interface of HER2. There would be a steric clash if m66 and pertuzumab were both bound, which explains why m66 and pertuzumab do not bind HER2 simultaneously. Superimposition of the HER2- HER3-NRG1 structure (PDB: 7MN5) and HER2-m66-trastuzumab structure shows that m66 is unable to suppress HER2 homodimerization and heterodimerization (Fig 6C).

In the tetrameric structure of HER2-r40-trastuzumab-pertuzumab, r40 targets the ECD III/IV of HER2, a site distinct from the dimerization interface of HER2 (Fig 6B, 6C). The superimposed structure of HER2-HER3-NRG1 (PDB: 7MN5) and HER2- r40-trastuzumab-pertuzumab structure ~~indicates~~ showed that the epitope of antibody r40 is opposite to the dimerization interfaces of HER2, which indicates r40 ~~dose~~ did not directly disturb the dimerization of HER2 (Fig 6C). The large interface between r40 and HER2 ~~indicates~~ establishes the high affinity and inhibitory activity of r40 on HER2.

# Discussion

Antibody drugs have revolutionized cancer treatment by specifically targeting tumor antigens, thereby enhancing therapeutic efficacy and reducing side effects, making them a cornerstone of modern oncology. Two approved anti-HER2 antibodies, trastuzumab and pertuzumab, specifically target distinct regions of the HER2 protein: trastuzumab targets the juxtamembrane region of ECD IV, while pertuzumab binds near the core of ECD II [10]. Till now, no antibody therapeutics have been identified that selectively target the ECD I and ECD III of HER2. Nevertheless, ongoing studies are exploring new HER2 epitopes to develop more effective treatment strategies.

To obtain antibodies targeting distinct epitopes from those recognized by trastuzumab and pertuzumab, we performed ELISA for mouse antibodies and flow cytometry for rabbit antibodies with saturated trastuzumab and pertuzumab. The data show that m66 competes with pertuzumab and r40 may recognize a different epitope from that of trastuzumab and pertuzmab. The Cryo-EM structures further confirm the epitopes of m66 and r40 antibodies.

In the absence of ligands, overexpressed HER2 can form homodimers[25]. EGF and NRG1 play pivotal roles in the activation of HER2 signaling. Upon binding to EGFR, EGF triggers a conformational change that extends EGFR from a bent form to an extended state, thereby revealing its dimerization arm. This arm of HER2 then engages with the binding pocket of EGFR, establishing a stable EGFR/HER2 heterodimer[29]. NRG1, through its EGF-like domain, binds to HER3 and HER4, thereby promoting the heterodimerization of HER2/HER3 and HER2/HER4[30, 31]. These three heterodimers induce activation of the downstream PI3K/AKT and MAPK signaling pathways, thereby drive the growth of cells. In our study, we demonstrated that r40 effectively inhibits the signaling pathways in both ligand-independent and ligand-dependent manners.

Trastuzumab has been shown to facilitate the internalization of HER2, directing it into endosomes, and subsequently routing it to lysosomes for degradation[32]. In both ligand-dependent and ligand-independent contexts, we observed that the r40 antibody, ~~similar to~~ like trastuzumab, effectively diminished the expression levels of HER2 protein. Conversely, the m66 antibody, similar to pertuzumab, ~~has~~ had no influence on HER2 protein levels (Fig 4, 5). We hypothesize that r40 induces the internalization of HER2,

whereas m66 does not. One potential reason is that r40 exhibits higher affinity than m66, and the stable antigen-antibody complex structure triggers the internalization process. Another possible explanation is that the binding of m66 to ECD I does not induce conformational changes, however the epitope targeted by the r40 antibody is situated at the interface between ECD III and IV, and conformational shifts within these domains are more prone to trigger the internalization of HER2.

In summary, we screened a panel of human HER2 antibodies and found ~~characterized~~ that r40 is a HER2 ECD III/IV-specific antibody~~, which~~ that downregulates HER2 protein level, disrupts PI3K/AKT and MAPK pathways and suppresses the growth of breast cancer cells. Antibody r40 is anticipated to enhance therapeutic efficacy when used in combination with trastuzumab and pertuzumab, suggesting its potential for HER2 overexpressing cancers. Nevertheless, to explore more functions and elucidate the complete mechanism, more detailed analyses and humanization of r40 will be needed in the future.

## References

1. Citri A, Yarden Y. EGF-ERBB signalling: towards the systems level. Nat Rev Mol Cell Biol. 2006;7(7):505-16. doi: 10.1038/nrm1962. PubMed PMID: 16829981.
2. Weisser NE, Sanches M, Escobar-Cabrera E, O'Toole J, Whalen E, Chan PWY, et al. An anti-HER2 biparatopic antibody that induces unique HER2 clustering and complement-dependent cytotoxicity. Nature communications. 2023;14(1):1394-. doi: 10.1038/s41467-023-37029-3. PubMed PMID:

36914633.

3. Tzahar E, Waterman H, Chen X, Levkowitz G, Karunagaran D, Lavi S, et al. A hierarchical network of interreceptor interactions determines signal transduction by Neu differentiation factor/neuregulin and epidermal growth factor. Mol Cell Biol. 1996;16(10):5276-87. doi: 10.1128/MCB.16.10.5276. PubMed PMID: 8816440; PubMed Central PMCID: PMC231527.
4. Junttila TT, Akita RW, Parsons K, Fields C, Lewis Phillips GD, Friedman LS, et al. Ligand-independent HER2/HER3/PI3K complex is disrupted by trastuzumab and is effectively inhibited by the PI3K inhibitor GDC-0941. Cancer Cell. 2009;15(5):429-40. doi: 10.1016/j.ccr.2009.03.020. PubMed PMID: 19411071.
5. Lewis GD, Figari I, Fendly B, Wong WL, Carter P, Gorman C, et al. Differential responses of human tumor cell lines to anti-p185HER2 monoclonal antibodies. Cancer Immunol Immunother. 1993;37(4):255-63. doi: 10.1007/BF01518520. PubMed PMID: 8102322.
6. Clynes RA, Towers TL, Presta LG, Ravetch JV. Inhibitory Fc receptors modulate in vivo cytotoxicity against tumor targets. Nat Med. 2000;6(4):443-6. doi: 10.1038/74704. PubMed PMID: 10742152.
7. Nahta R, Esteva FJ. Herceptin: mechanisms of action and resistance. Cancer Lett. 2006;232(2):123-38. doi: 10.1016/j.canlet.2005.01.041. PubMed PMID: 16458110.
8. Franklin MC, Carey KD, Vajdos FF, Leahy DJ, de Vos AM, Sliwkowski MX. Insights into ErbB signaling from the structure of the ErbB2-pertuzumab complex. Cancer Cell. 2004;5(4):317-28. doi: 10.1016/s1535-6108(04)00083-

2. PubMed PMID: 15093539.

9. Adams CW, Allison DE, Flagella K, Presta L, Clarke J, Dybdal N, et al.

Humanization of a recombinant monoclonal antibody to produce a therapeutic HER dimerization inhibitor, pertuzumab. Cancer Immunol Immunother. 2006;55(6):717-27. doi: 10.1007/s00262-005-0058-x. PubMed PMID: 16151804.

10. Hao Y, Yu X, Bai Y, McBride HJ, Huang X. Cryo-EM Structure of HER2- trastuzumab-pertuzumab complex. PLoS One. 2019;14(5):e0216095. doi: 10.1371/journal.pone.0216095. PubMed PMID: 31042744; PubMed Central PMCID: PMC6493747.
11. Scheuer W, Friess T, Burtscher H, Bossenmaier B, Endl J, Hasmann M. Strongly enhanced antitumor activity of trastuzumab and pertuzumab combination treatment on HER2-positive human xenograft tumor models. Cancer Res. 2009;69(24):9330-6. doi: 10.1158/0008-5472.CAN-08-4597. PubMed PMID:

19934333.

12. Nahta R, Hung MC, Esteva FJ. The HER-2-targeting antibodies trastuzumab and pertuzumab synergistically inhibit the survival of breast cancer cells. Cancer Res. 2004;64(7):2343-6. doi: 10.1158/0008-5472.can-03-3856. PubMed PMID: 15059883.
13. von Minckwitz G, Procter M, de Azambuja E, Zardavas D, Benyunes M, Viale G, et al. Adjuvant Pertuzumab and Trastuzumab in Early HER2-Positive Breast Cancer. N Engl J Med. 2017;377(2):122-31. doi: 10.1056/NEJMoa1703643. PubMed PMID: 28581356; PubMed Central PMCID: PMC5538020.
14. Bilancia D, Rosati G, Dinota A, Germano D, Romano R, Manzione L. Lapatinib in breast cancer. Ann Oncol. 2007;18 Suppl 6:vi26-30. doi: 10.1093/annonc/mdm220. PubMed PMID: 17591827.
15. Sonpavde G. Lapatinib plus capecitabine in breast cancer. N Engl J Med. 2007;356(14):1471; author reply -2. doi: 10.1056/NEJMc070182. PubMed PMID: 17409332.
16. Lopez-Tarruella S, Jerez Y, Marquez-Rodas I, Martin M. Neratinib (HKI-272) in the treatment of breast cancer. Future Oncol. 2012;8(6):671-81. doi: 10.2217/fon.12.66. PubMed PMID: 22764764.
17. Verma S, Miles D, Gianni L, Krop IE, Welslau M, Baselga J, et al. Trastuzumab emtansine for HER2-positive advanced breast cancer. N Engl J Med. 2012;367(19):1783-91. doi: 10.1056/NEJMoa1209124. PubMed PMID:

23020162; PubMed Central PMCID: PMC5125250.

18. Cortes J, Hurvitz SA, Im SA, Iwata H, Curigliano G, Kim SB, et al. Trastuzumab deruxtecan versus trastuzumab emtansine in HER2-positive metastatic breast cancer: long-term survival analysis of the DESTINY-Breast03 trial. Nat Med. 2024;30(8):2208-15. doi: 10.1038/s41591-024-03021-7. PubMed PMID:

38825627; PubMed Central PMCID: PMC11333275.

19. Oh DY, Bang YJ. HER2-targeted therapies - a role beyond breast cancer. Nature Reviews Clinical Oncology. 2020;17(1):33-48. doi: 10.1038/s41571- 019-0268-3. PubMed PMID: 31548601.
20. Shitara K, Bang YJ, Iwasa S, Sugimoto N, Ryu MH, Sakai D, et al. Trastuzumab deruxtecan in HER2-positive advanced gastric cancer: exploratory biomarker analysis of the randomized, phase 2 DESTINY-Gastric01 trial. Nat Med. 2024;30(7):1933-42. doi: 10.1038/s41591-024-02992-x. PubMed PMID:

38745009; PubMed Central PMCID: PMC11271396.

21. Okines AF, Cunningham D. Trastuzumab: a novel standard option for patients with HER-2-positive advanced gastric or gastro-oesophageal junction cancer. Therap Adv Gastroenterol. 2012;5(5):301-18. doi: 10.1177/1756283X12450246. PubMed PMID: 22973416; PubMed Central PMCID: PMC3437538.
22. Rexer BN, Arteaga CL. Intrinsic and acquired resistance to HER2-targeted therapies in HER2 gene-amplified breast cancer: mechanisms and clinical implications.	Crit	Rev	Oncog.	2012;17(1):1-16.	doi: 10.1615/critrevoncog.v17.i1.20. PubMed PMID: 22471661; PubMed Central PMCID: PMC3394454.
23. Liu X, Song Y, Cheng P, Liang B, Xing D. Targeting HER2 in solid tumors: Unveiling the structure and novel epitopes. Cancer Treat Rev. 2024;130:102826. doi: 10.1016/j.ctrv.2024.102826. PubMed PMID: 39270365.
24. Pedersen MW, Jacobsen HJ, Koefoed K, Dahlman A, Kjaer I, Poulsen TT, et al. Targeting Three Distinct HER2 Domains with a Recombinant Antibody Mixture Overcomes Trastuzumab Resistance. Mol Cancer Ther. 2015;14(3):669-80. doi: 10.1158/1535-7163.MCT-14-0697. PubMed PMID:

25612619.

25. Meng Y, Zheng L, Yang Y, Wang H, Dong J, Wang C, et al. A monoclonal

antibody targeting ErbB2 domain III inhibits ErbB2 signaling and suppresses the growth of ErbB2-overexpressing breast tumors. Oncogenesis. 2016;5(3):e211. doi: 10.1038/oncsis.2016.25. PubMed PMID: 26999718;

PubMed Central PMCID: PMC4815051.

26. Fu W, Wang Y, Zhang Y, Xiong L, Takeda H, Ding L, et al. Insights into HER2 signaling from step-by-step optimization of anti-HER2 antibodies. MAbs. 2014;6(4):978-90. doi: 10.4161/mabs.28786. PubMed PMID: 24838231;

PubMed Central PMCID: PMC4171031.

27. Tamaskovic R, Schwill M, Nagy-Davidescu G, Jost C, Schaefer DC, Verdurmen WP, et al. Intermolecular biparatopic trapping of ErbB2 prevents compensatory activation of PI3K/AKT via RAS-p110 crosstalk. Nat Commun. 2016;7:11672. doi: 10.1038/ncomms11672. PubMed PMID: 27255951;

PubMed Central PMCID: PMC4895728.

28. Kast F, Schwill M, Stuber JC, Pfundstein S, Nagy-Davidescu G, Rodriguez JMM, et al. Engineering an anti-HER2 biparatopic antibody with a multimodal mechanism of action. Nat Commun. 2021;12(1):3790. doi: 10.1038/s41467- 021-23948-6. PubMed PMID: 34145240; PubMed Central PMCID: PMC8213836.
29. Yu X, Wang L, Shen Y, Wang C, Zhang Y, Meng Y, et al. Targeting EGFR/HER2 heterodimerization with a novel anti-HER2 domain II/III antibody. Molecular Immunology. 2017;87:300-7. doi: 10.1016/j.molimm.2017.05.010. PubMed PMID: 28531814.
30. Diwanji D, Trenker R, Thaker TM, Wang F, Agard DA, Verba KA, et al. Structures of the HER2-HER3-NRG1beta complex reveal a dynamic dimer interface. Nature. 2021;600(7888):339-43. doi: 10.1038/s41586-021-04084-

z. PubMed PMID: 34759323; PubMed Central PMCID: PMC9298180.

31. Trenker R, Diwanji D, Bingham T, Verba KA, Jura N. Structural dynamics of the active HER4 and HER2/HER4 complexes is finely tuned by different growth factors and glycosylation. Elife. 2024;12. doi: 10.7554/eLife.92873. PubMed PMID: 38498590; PubMed Central PMCID: PMC10948148.
32. Baselga J, Albanell J. Mechanism of action of anti-HER2 monoclonal antibodies. Ann Oncol. 2001;12 Suppl 1:S35-41. doi: 10.1093/annonc/12.suppl_1.s35. PubMed PMID: 11521720.
33. Zheng SQ, Palovcak E, Armache JP, Verba KA, Cheng Y, Agard DA. MotionCor2: anisotropic correction of beam-induced motion for improved cryo-electron microscopy. Nat Methods. 2017;14(4):331-2. doi: 10.1038/nmeth.4193. PubMed PMID: 28250466; PubMed Central PMCID: PMC5494038.
34. Zhang K. Gctf: Real-time CTF determination and correction. J Struct Biol. 2016;193(1):1-12. doi: 10.1016/j.jsb.2015.11.003. PubMed PMID: 26592709;

PubMed Central PMCID: PMC4711343.

35. Scheres SH. RELION: implementation of a Bayesian approach to cryo-EM structure determination. J Struct Biol. 2012;180(3):519-30. doi: 10.1016/j.jsb.2012.09.006. PubMed PMID: 23000701; PubMed Central PMCID: PMC3690530.
36. Kimanius D, Forsberg BO, Scheres SH, Lindahl E. Accelerated cryo-EM structure determination with parallelisation using GPUs in RELION-2. Elife. 2016;5. doi: 10.7554/eLife.18722. PubMed PMID: 27845625; PubMed Central PMCID: PMC5310839.
37. Punjani A, Rubinstein JL, Fleet DJ, Brubaker MA. cryoSPARC: algorithms for rapid unsupervised cryo-EM structure determination. Nat Methods. 2017;14(3):290-6. doi: 10.1038/nmeth.4169. PubMed PMID: 28165473.
38. Pettersen EF, Goddard TD, Huang CC, Couch GS, Greenblatt DM, Meng EC, et al. UCSF Chimera--a visualization system for exploratory research and analysis. J Comput Chem. 2004;25(13):1605-12. doi: 10.1002/jcc.20084. PubMed PMID: 15264254.
39. Tunyasuvunakool K, Adler J, Wu Z, Green T, Zielinski M, Zidek A, et al. Highly accurate protein structure prediction for the human proteome. Nature. 2021;596(7873):590-6. doi: 10.1038/s41586-021-03828-1. PubMed PMID:

34293799; PubMed Central PMCID: PMC8387240.

40. Emsley P, Cowtan K. Coot: model-building tools for molecular graphics. Acta Crystallogr D Biol Crystallogr. 2004;60(Pt 12 Pt 1):2126-32. doi: 10.1107/S0907444904019158. PubMed PMID: 15572765.

## Acknowledgments

We thank the Center of Cryo-Electron Microscopy, Core Facility of Shanghai Medical College, Fudan University for the supports on cryo-EM data collection and data analyses. This work was supported by grants from the Shanghai Natural Science Foundation of China (21ZR1407900).

## Author contributions

H. Y. and Y. X. supervised the project; C. L. purified the antibodies and conducted the sample preparation for structural and biochemical studies; C. L. and X. L. performed the cell assay; Y. R. and Q. W. conducted cryo-EM data collection, data analyses and model building; D. Z., P. Z., X. D. screened the antibodies; X. H. helped the flow cytometry assay; C. L. and H. Y. performed data analysis, genetated the figures and drafted the manuscript with contributions from Y. X.

## Competing interests

Authors declare no competing interests.

## Data and materials availability

The cryo-EM density maps and corresponding atomic coordinates will be made available upon manuscript acceptance.

# Materials and Methods

## Cell culture

BT-474, SK-BR-3 and MCF7 were acquired from the American Type Culture Collection (ATCC). These cells were maintained in DMEM medium (Gibco) supplemented with 10% fetal bovine serum (NEWZERUM) and penicillin/streptomycin (Basalmedia) at the concentration of 100 μg/mL. Expi293 cells were cultured in Union 293 cell feed medium (Union) containing 100 μg/mL penicillin/streptomycin.

## Construction and expression

The DNA sequence encoding human HER2 ECD (residues 23-652) was amplified and inserted into a modified pCAG vector containing a signal peptide and an N-terminal 9× His tag, enabling the production of a secreted HER2 ECD fusion protein. There is a HRV 3C protease recognition site between the His tag and HER2 ECD sequence (S1A Fig). The DNA sequence encoding the 55 amino-acid EGF like domain of human NRG1 (GTSHLVKCAEKEKTFCVNGGECFMVKDLSNPSRYLCKCPNEFTGDRCQNYV

MASF) was synthesized and cloned into the same modified pCAG vector (S1A Fig). The fusion proteins were purified from the culture medium of Expi293 cells using Ni- NTA Sepharose Fast Flow (GE Healthcare)~~,~~ and subsequently cleaved with HRV 3C protease to remove the His tag.

## Kinetics and affinity measurement

Binding kinetics of antibodies with HER2 were determined by SPRi using a PlexArray system (Plexera). IgG format antibodies (50 μg/mL) were immobilized on an 3D optical crosslinking biosensor, and then a of serial dilutions of HER2 (1-128 nM) in PBS were subjected to a process of 285 s association and 930 s dissociation.

EC50 of m66 and r40 was measured with ELISA. In brief, ELISA plates (Corning) were coated with 0.2 ng/μL of HER2 solution overnight at 4 °C. After blocking, a serial of concentrations (2^-11^-2^3^ ng/μL) were added to the plate and incubated for another 1 h at 37 °C. Either HRP-conjugated goat anti-human IgG1 Fc antibody (Millipore, AP113P) or HRP-conjugated goat anti-rabbit IgG (H+L) antibody (Abclonal, AS014) was incubated for 30 min-1 h at 37 °C. After washing with PBST, TMB substrate

(Beyotime, P0206) was added. The reaction was quenched with 1 M HCl, followed by absorbance measurement at 450 nm using a microplate reader (Biotek). EC50 estimates were obtained by fitting ELISA binding curves in Graphpad Prism using the log versus response-variable slope model.

## Competitive binding assay

To map epitopes of mouse antibodies, immobilized HER2 was ~~blocking~~ blocked with saturating non-His-tagged IgG, the subsequent binding of His-tagged IgG protein was measured with anti-his-HRP (Genscript, A00612) using ELISA.

To characterize the epitopes of rabbit antibodies, Expi 293 cells were suspended at 1× 10^7^ cells/mL and incubated with or without saturating concentrations of trastuzumab and pertuzumab for 0.5 h. After washing, the cells were further incubated with rabbit monoclonal antibodies for additional 0.5 h. Cells were stained with AF488- conjugated goat anti-rabbit IgG secondary antibody (Invitrogen, A11008), and subsequently analyzed using a flow cytometer (Thermo Fisher Scientific) and the FlowJo software (state version of FlowJo used).

## Flow cytometric analysis

BT-474, SK-BR-3 and MCF7 were briefly trypsinized at 37°C, quenched with FBS- containing medium and removed by centrifugation. ~~A number of~~ 600,000 cells for each sample were suspended in FACS buffer (1× PBS containing 2% FBS), followed by incubation with 10 μg/mL trastuzumab, pertuzumab, m66, r40 for 0.5 h - 1 h at 4 °C. Human IgG isotype (Beyotime) and rabbit IgG isotype (Beyotime) were used as the isotype control. Then the cells were incubated for an additional 0.5 h with either AF647- conjugated goat anti-human secondary antibody (Invitrogen, A21445) or AF488- conjugated goat anti-rabbit secondary antibody. Cells were analyzed via flow ~~cytometery~~ cytometry after staining, and histograms of anti-HER2 antibodies were compared with their respective isotype controls.

## Cell proliferation assay

9×10^3^ BT-474, 3×10^3^ SK-BR-3 and 5×10^3^ MCF7 cells were plated in ~~the~~ flatbottomed 96-well cell culture plates and incubated overnight. Gradient concentrations of antibodies (100, 20, 4, 0.8, 0.16, 0 μg/mL) were added to the wells. Cell proliferation was quantitatively determined using Cell Counting Kit-8 (Beyotime, C0042) following

an additional 4-day incubation.

## Immunoblotting

BT-474, SK-BR-3 and MCF7 cells were treated with 50 μg/mL trastuzumab, pertuzumab, m66, r40 or the combination of trastuzumab and pertuzumab (each at 50 μg/mL) for either 6 h or 24 h. Recombinant human NRG1 or EGF (Acro biosystems, EGF-H52H3) were supplemented at the final concentration of 0.5 nM and 5 nM respectively for 10 min. Following washing with ice-cold 1× PBS, the cells were lysed and the cell lysates were harvested and analyzed using reducing 4-20% gradient SDS- PAGE, followed by immunoblotting with antibodies targeting GAPDH (Beyotime, AF0006), Tubulin (Abclonal, AC012), AKT (Cell Signaling Technology, 9272), phospho-AKT (Ser473) (Cell Signaling Technology, 4058), p44/42 MAPK (Erk1/2) (Cell Signaling Technology, 4695), phospho-p44/42 MAPK (Erk1/2) (Cell Signaling Technology, 4370) or HER2 (Cell Signaling Technology, 4290).

## Fab purification and complex formation

The heavy chain and light chain expression cassettes of four Fabs (trastuzumab, pertuzumab, m66 and r40) were assembled into a redesigned pCAG vector. One 9× His tag was incorporated into the C-terminal of heavy chain constant domain. Fab fragments were obtained by co-transfection of light chain and heavy chain into suspension Expi 293 cells using PEI (Yeasen, 40816ES08). Following Ni-NTA purification, eluent was dialyzed to remove imidazole.

For HER2-m66-trastuzumab complex, HER2 was pretreated with an excess amount of m66 Fab. The resulting mixture was ~~seperated~~ separated by SEC using a Superdex 200 10/300 column (GE Healthcare) in 20 mM HEPES pH 7.4, 150 mM NaCl. The HER2- m66 binary complex was collected and further incubated with excessive trastuzumab Fab to form the HER2-m66-trastuzumab complex. HER2-r40-trastuzumab-pertuzumab complex was also prepared using a similar procedure.

## Cryo-EM sample preparation and data collection

For cryo-EM grid preparation, 3 μL of 1.5 mg/mL HER2-m66-trastuzumab and HER2- r40-trastuzuamb-pertuzumab were loaded onto the freshly glow-discharged Quantifoil R1.2/1.3 Au grids. Samples were vitrified by a FEI Vitrobot Mark IV with one second blot time at 100% humidity. Subsequently, the grids were applied to a Titan Krios G3i

or G4 transmission electron microscope (Thermo Fisher Scientific) with 300 kV accelerating voltage for data collection and characterization. In total, 992 micrographs of HER2-r40-trastuzumab-pertuzumab were automatically recorded via a Titan Krios G3i transmission electron microscope equipped with Gatan K3 direct electron detector for characterization. Micrographs of HER2-r40-trastuzumab-pertuzumab were captured with Serial-EM software in the super-resolution counting mode. The instrument was operated at a ~~norminal~~ nominal? magnification of 81,000× in the EFTEM mode, leading to a super-resolution pixel size of 0.532 Å on the image plane. The defocus values spanned a range of -1.5 μm to -2.5 μm. The micrograph stack was dose-divided into 40 individual frames for dose-per-frame, with each frame receiving approximately 50 e-/ Å^2^ and 2.3 s exposure time. The other images were captured automatically using a Titan Krios G4 transmission electron microscope equipped with a Falcon 4i direct electron detector. The micrographs were recorded in super-resolution counting mode using the EPU software at a ~~norminal~~ nominal magnification of 130,000× in the NPTEM mode. This configuration achieved a super-resolution pixel size of 0.466 Å on the image plane. With defocus values spanning between -1.5 μm to -2.5 μm, each micrograph stack was dose-fractionated into 1080 frames, receiving approximately 50 e-/ Å^2^ total electron dose, under 3.51 s exposure time. For further processing, 2,439 and 2,965 micrographs were systematically acquired for the datasets of HER2-m66-trastuzumab and HER2- r40-trastuzumab-pertuzumab, respectively.

## Cryo-EM data processing

The processing of all cryo-EM datasets involved addressing and correcting drift and beam-induced motion corrections to achieve whole-frame movie alignment via MotionCor2[33]. The micrographs from the Titan Krios G3i were subjected to a binning process with a factor of 2 to achieve a calibrated pixel size of 1.064 Å/pixel, while the Titan Krios G4 datasets were binned similarly and down to 0.932 Å/pixel. Defocus values were determined using Gctf[34] on summed image data, with dose weighting not applied. Subsequent cryo-EM data were processed using RELION v3.1[35, 36] and cryoSPARC v2[37], employing dose-weighted images for analysis.

For HER2-m66-trastuzumab dataset, 2,017,757 particles were automatically picked in RELION with no reference and subsequently subjected to reference-free 2D classification in cryoSPARC. In total, 466,601 particles were selected from good 2D

classes for the 3D initial model. In all, 584,245 were selected from high-quality 3D classes and utilized for heterogeneous refinement in cryoSPARC. The refined particles were loaded to RELION again for 2D and 3D classification. A total of 282,644 particles were extracted from well-defined 3D class assignments and subjected to heterogeneous refinement in cryoSPARC, with CTF correction, achieving the HER2-m66-trastuzumab complex at 3.2 Å resolution.

For the dataset of HER2-r40-trastuzumab-pertuzumab acquired using the Titan Krios G3i, an automatic particle selection process yielded 1,490,027 particles, followed by 2D classification analysis using RELION. From the well-characterized 2D classes, 56,640 particles were chosen to initiate the 3D classification process. Ultimately, 24,229 particles were extracted from high-quality 3D classes and subjected to 3D auto- refinement, achieving a 9.1 Å resolution reconstruction of the HER2-r40-trastuzumab- pertuzumab complex.

For the dataset of HER2-r40-trastuzumab-pertuzumab from Titan Krios G4, leveraging the previously achieved 9.1 Å resolution structure as a template, 2,293,703 particles were automatically selected and underwent 2D classification using RELION. From the well-defined 2D classes, a selection of 1,973,712 particles was made to proceed with the preliminary 3D classification analysis. A total of 450,219 particles were extracted from high-quality 3D classes and subsequently utilized for 3D auto- refinement. After undergoing Bayesian polishing, post-processing and CTF refinement, this process achieved a 3.1 Å resolution reconstruction of the HER2-r40-trastuzumab- pertuzumab complex.

All observed resolutions were established in accordance with the gold-standard Fourier shell correlation (FSC) criterion of 0.143 and the gold-standard FSC curves were validated to address the effects of a soft mask with high-resolution noise substitution. The cryo-EM maps were processed within the cryoSPARC software using module-based tools for B-factor optimization. 3D volume mapping was visualized and evaluated using UCSF Chimera or UCSF ChimeraX[38], with local resolution variations determined via RELION analysis.

## Model building and structure refinement

With the structure of HER2-trastuzumab-pertuzumab (PDB: 6OGE)[10] serving as the

initial structural template, the cryo-EM maps were utilized for model fitting. The HER2-trastuzumab ~~compenent~~ spelling? Component? and the m66 structure from AlphaFold prediction[39] were docked into corresponding cryo-EM maps of HER2-m66-trastuzumab via rigid- body fitting in UCSF Chimera[38]. The HER2-trastuzumab-pertuzumab structure (PDB: 6OGE) and the r40 structure predicted by AlphaFold were incorporated into the cryo-EM maps of HER2-r40-trastuzumab-pertuzumab using UCSF Chimera for rigid-body fitting. Structural models were generated using COOT[40]. Maps and models in the figures were created using PyMOL (https://pymol.org/), UCSF Chimera, or UCSF ChimeraX. Statistics of the map reconstruction and model refinement with detailed parameters are available in S1 Table.

## Figure Legends

**Fig. 1. Relative epitopes were assessed by ELISA and flow cytometry.** A. Binding of His-tagged IgG to immobilized HER2 was assessed via ELISA under conditions with or without saturating concentrations of non-His-tagged IgG. B. The binding of rabbit antibodies to HER2 on the cell surface of Expi293 cells was evaluated, with results compared to those obtained using isotype control IgG alone. C. Flow cytometry results for six rabbit antibodies, with or without saturating concentrations of trastuzumab and pertuzumab. D. Rabbit antibodies epitope mapping by flow cytometry. The x-axis represents the clone identification of rabbit antibodies, while the y-axis shows the ratio of the mean fluorescence, comparing the presence and absence of saturating concentrations of trastuzumab and pertuzumab. Error bars, SD. *, P < 0.05; **, P < 0.01; ***, P < 0.001; ****, P

< 0.0001, T-test.

**Fig. 2. HER2-expressing tumor cell proliferation inhibition and KD measurement of m66 and r40.** A. Cell proliferation inhibition activity of nine antibodies and the combination of trastuzumab and pertuzumab were measured by CCK8 assay in BT-474. B. The inhibitory effects on cell proliferation induced by antibodies were conducted in SK-BR-3. C. NRG1-independent and NRG1- dependent tumor cell proliferation was conducted in MCF7 cells. Results are depicted as the percentage relative of control cell proliferation. C, control; T, trastuzumab; P, pertuzumab. Error bars, SD. *, P < 0.05; **, P < 0.01; ***, P < 0.001; ****, P < 0.0001, T-test. D. Dissociation constant measurement of trastuzumab, pertuzumab, m66 and r40 against HER2 by SPR assay.

**Fig. 3. Binding profiles of trastuzumab, pertuzumab, m66 and r40 to tumor cell lines expressing HER2.** BT-474, SK-BR-3 and MCF7 were incubated with 10 μg/mL of the antibodies, followed by staining with fluorescence-labeled secondary antibodies. The samples were analyzed via flow cytometry.

**Fig. 4. Antibody r40 inhibits ligand-independent HER2 signaling in BT-474 and SK-BR-3.** Immunoblots assessing HER2 signaling upon treatment with 50 μg/mL antibodies for 6 h or 24 h in BT-474 (A) and SK-BR-3 (B). For the combination treatment of trastuzumab and pertuzumab, the final concentration of each antibody is 50 μg/mL.

**Fig. 5. Antibody r40 inhibits ligand-dependent signaling.** A. Immunoblots assessing HER2 downstream signaling in BT-474, SK-BR-3 and MCF7 upon incubation with 50 μg/mL antibodies for 24 h under 5 nM EGF stimulation. B. Immunoblots assessing HER2 downstream signaling in MCF7 cells with 50 μg/mL antibodies treatment for 6 h or 24 h in the presence of 0.5 nM NRG1.

**Fig. 6. Cryo-EM structures of HER2-m66-trastuzumab and HER2-r40-trastuzumab- pertuzumab are determined.** A. Cryo-EM map depicting ~~the~~ the complex composed of three components: HER2 (sky blue), m66 Fab (orange), and trastuzumab Fab (pink), and the final model (grey surface) incorporating the three components into the map. B. Cryo-EM map ~~illustrating~~ illustrated the tetrameric complex consisting of four components: HER2 (sky blue), r40 Fab (green), trastuzumab Fab (pink) and pertuzumab Fab (yellow), and the final construction (grey surface) with the four proteins fitted into the map. C. Superposition of HER2-trastuzumab- pertuzumab structure (PDB: 6OGE) and HER2-m66-trastuzumab structure. Superposition of HER2-HER3-NRG1 structure (PDB: 7MN5) and HER2-m66-trastuzumab structure. Overall structure of HER2-r40-trastuzumab-pertuzumab. Superposition of HER2-HER3-NRG1 structure (PDB: 7MN5) and HER2-r40-trastuzumab-pertuzumab complex.

Figure 1	[Click here to access/download;Figure;Fig 1.png](https://www2.cloud.editorialmanager.com/pone/download.aspx?id=39661521&guid=7ca4dab9-652c-407c-8da6-ff16e9237947&scheme=1)[<https://www2.cloud.editorialmanager.com/pone/download.aspx?id=39661521&guid=7ca4dab9-652c-407c-8da6-ff16e9237947&scheme=1>](https://www2.cloud.editorialmanager.com/pone/download.aspx?id=39661521&guid=7ca4dab9-652c-407c-8da6-ff16e9237947&scheme=1)

Figure 2	[Click here to access/download;Figure;Fig 2.png](https://www2.cloud.editorialmanager.com/pone/download.aspx?id=39661522&guid=e2baeb23-e7da-4584-b60d-5cc7eb6f9088&scheme=1)[<https://www2.cloud.editorialmanager.com/pone/download.aspx?id=39661522&guid=e2baeb23-e7da-4584-b60d-5cc7eb6f9088&scheme=1>](https://www2.cloud.editorialmanager.com/pone/download.aspx?id=39661522&guid=e2baeb23-e7da-4584-b60d-5cc7eb6f9088&scheme=1)

Figure 3	[Click here to access/download;Figure;Fig 3.png](https://www2.cloud.editorialmanager.com/pone/download.aspx?id=39661523&guid=e15ee792-d40b-4e8d-b8a6-b72ed7a04d62&scheme=1)[<https://www2.cloud.editorialmanager.com/pone/download.aspx?id=39661523&guid=e15ee792-d40b-4e8d-b8a6-b72ed7a04d62&scheme=1>](https://www2.cloud.editorialmanager.com/pone/download.aspx?id=39661523&guid=e15ee792-d40b-4e8d-b8a6-b72ed7a04d62&scheme=1)

Figure 4	[Click here to access/download;Figure;Fig 4.png](https://www2.cloud.editorialmanager.com/pone/download.aspx?id=39661524&guid=d6d76ae5-21bd-49de-a916-673052b0edb1&scheme=1)[<https://www2.cloud.editorialmanager.com/pone/download.aspx?id=39661524&guid=d6d76ae5-21bd-49de-a916-673052b0edb1&scheme=1>](https://www2.cloud.editorialmanager.com/pone/download.aspx?id=39661524&guid=d6d76ae5-21bd-49de-a916-673052b0edb1&scheme=1)

Figure 5	[Click here to access/download;Figure;Fig 5.png](https://www2.cloud.editorialmanager.com/pone/download.aspx?id=39661526&guid=f11c850c-c7f3-453c-af00-32b921532bb9&scheme=1)[<https://www2.cloud.editorialmanager.com/pone/download.aspx?id=39661526&guid=f11c850c-c7f3-453c-af00-32b921532bb9&scheme=1>](https://www2.cloud.editorialmanager.com/pone/download.aspx?id=39661526&guid=f11c850c-c7f3-453c-af00-32b921532bb9&scheme=1)

Figure 6	[Click here to access/download;Figure;Fig 6.png](https://www2.cloud.editorialmanager.com/pone/download.aspx?id=39661528&guid=c1cd22be-a655-4361-b165-4e078653dc91&scheme=1)[<https://www2.cloud.editorialmanager.com/pone/download.aspx?id=39661528&guid=c1cd22be-a655-4361-b165-4e078653dc91&scheme=1>](https://www2.cloud.editorialmanager.com/pone/download.aspx?id=39661528&guid=c1cd22be-a655-4361-b165-4e078653dc91&scheme=1)

Supporting Information

[Click here to access/download](https://www2.cloud.editorialmanager.com/pone/download.aspx?id=39670257&guid=db737b26-abd7-4fad-bc7b-4c9a0e197d85&scheme=1)

[Supporting Information Supplement.docx](https://www2.cloud.editorialmanager.com/pone/download.aspx?id=39670257&guid=db737b26-abd7-4fad-bc7b-4c9a0e197d85&scheme=1)

S1 figure

[Click here to access/download](https://www2.cloud.editorialmanager.com/pone/download.aspx?id=39661579&guid=a016d4a1-9605-4410-a13a-760e516855c0&scheme=1)

[Supporting Information S1 Fig.png](https://www2.cloud.editorialmanager.com/pone/download.aspx?id=39661579&guid=a016d4a1-9605-4410-a13a-760e516855c0&scheme=1)

S2 figure

[Click here to access/download](https://www2.cloud.editorialmanager.com/pone/download.aspx?id=39661580&guid=45780f95-fc8c-4353-848e-889f61eed4c9&scheme=1)

[Supporting Information S2 Fig.png](https://www2.cloud.editorialmanager.com/pone/download.aspx?id=39661580&guid=45780f95-fc8c-4353-848e-889f61eed4c9&scheme=1)

S3 figure

[Click here to access/download](https://www2.cloud.editorialmanager.com/pone/download.aspx?id=39661581&guid=8ddfde85-d9c9-48c2-a21d-1a94712138c1&scheme=1)

[Supporting Information S3 Fig.png](https://www2.cloud.editorialmanager.com/pone/download.aspx?id=39661581&guid=8ddfde85-d9c9-48c2-a21d-1a94712138c1&scheme=1)

S4 figure

[Click here to access/download](https://www2.cloud.editorialmanager.com/pone/download.aspx?id=39661582&guid=80af5815-0b6a-4aa3-84e2-5e1f45b0bde0&scheme=1)

[Supporting Information S4 Fig.png](https://www2.cloud.editorialmanager.com/pone/download.aspx?id=39661582&guid=80af5815-0b6a-4aa3-84e2-5e1f45b0bde0&scheme=1)

S5 figure

[Click here to access/download](https://www2.cloud.editorialmanager.com/pone/download.aspx?id=39661583&guid=4ac494da-73c1-4ace-adc2-e6ce8e96074a&scheme=1)

[Supporting Information S5 Fig.png](https://www2.cloud.editorialmanager.com/pone/download.aspx?id=39661583&guid=4ac494da-73c1-4ace-adc2-e6ce8e96074a&scheme=1)

Fig 4 raw images

[Click here to access/download](https://www2.cloud.editorialmanager.com/pone/download.aspx?id=39671577&guid=27409c3d-f79e-48c7-aaf9-4c725bfac6cf&scheme=1)

[Supporting Information Fig 4_raw_images.pdf](https://www2.cloud.editorialmanager.com/pone/download.aspx?id=39671577&guid=27409c3d-f79e-48c7-aaf9-4c725bfac6cf&scheme=1)

Fig 5 raw images

[Click here to access/download](https://www2.cloud.editorialmanager.com/pone/download.aspx?id=39671487&guid=6ff53446-66ef-4557-ae71-5065205fdc5e&scheme=1)

[Supporting Information Fig 5_raw_images.pdf](https://www2.cloud.editorialmanager.com/pone/download.aspx?id=39671487&guid=6ff53446-66ef-4557-ae71-5065205fdc5e&scheme=1)

S1 figure raw images

[Click here to access/download](https://www2.cloud.editorialmanager.com/pone/download.aspx?id=39671488&guid=619e90cf-8807-4e8f-beaf-76ebf0b57962&scheme=1)

[Supporting Information S1_raw_images.pdf](https://www2.cloud.editorialmanager.com/pone/download.aspx?id=39671488&guid=619e90cf-8807-4e8f-beaf-76ebf0b57962&scheme=1)
