# Supplementary material for: Inhibition of HER2 signaling and breast cancer cell growth with a novel antibody targeting HER2 ECD III/IV
Source: PLoS One. 2026 Jan 15;21(1):e0338127. doi: 10.1371/journal.pone.0338127 (PMC12806845; doi:10.1371/journal.pone.0338127)
Supplement: Attachment — Submitted filename: Response to Reviewers.docx [file pone.0338127.s011.docx]

1. **Antibody generation:**
   a. **What formats were the recombinant antibodies produced using the selected sequences from B cells?**

For cryo-EM structural characterization, we employed Fab fragments; all functional experiments utilized full-length IgG antibodies.

b. **Protocols for ELISA and “pull-down” screening assays have to be provided.**

Thanks for the kind reminder, we have provided the protocols for ELISA and pull-down as follows:

ELISA

HER2 ECD protein was diluted to 0.2 µg/mL in 1× PBS and incubated overnight at 4°C. After blocking, the medium of recombinant expression IgG was diluted 1:100 in blocking buffer (PBST + 3% BSA) and incubated for 1 h. After washing, the binding of the recombinant expression IgGs was measured with HRP-conjugated goat anti-human IgG1 Fc antibody (Millipore, AP113P) or HRP-conjugated goat anti-rabbit IgG (H+L) antibody (Abclonal, AS014).

Pull-down

20 μL of 20% slurry rProtein A/G magnetic beads (SMART, SM015010), pre-washed with washing buffer (PBS + 0.1% CHAPS), were incubated with 10 μg of antibody at room temperature for 1 h. The beads were then washed three times with washing buffer and aliquoted equally into two portions. One portion served as a negative control, the other portion was incubated with 20 μg HER2 ECD protein at room temperature for 1 h and washed three times with washing buffer, followed by SDS-PAGE analysis.

Fig. 1. A. SDS-PAGE of mouse antibody pull-down. B-D. SDS-PAGE of rabbit antibody pull-down.

In Figure 1, panel A demonstrates pull-down assay results for mouse antibodies m66 and m75, while panel B-D show results for the 39 rabbit antibodies. All antibodies except r35 demonstrated antigen-positive binding.

c. **How did the authors confirmed selectivity and specificity of the antibodies?**

We assessed the selectivity and specificity of the antibodies by ELISA, pull-down, SPRi and flow cytometry assays, which show that the screened antibodies are specific for HER2.

2. **Competition assay**
   a. **What is “supersaturating IgG” [concentration] – how it was determined? What was the concentration of His-tagged IgG? There was no single dose-response binding curve for any reported antibody in the whole manuscript.**

Fig. 2. The EC50 values of trastuzumab, pertuzumab, m66, m75 and r40 with immobilized HER2 ECD protein was determined by ELISA.

The EC_50_ values of trastuzumab, pertuzumab, m66, m75 were all at the ng/mL level (Figure 2). Therefore, 2 μg/mL non-His-tagged IgG is sufficient to completely block all corresponding binding epitopes on HER2 ECD protein.

To map epitopes of mouse antibodies by ELISA, HER2 ECD protein was diluted to 0.2 µg/mL in 1× PBS and incubated overnight at 4°C. After blocking, non-His-tagged IgG was diluted to 2 µg/mL in blocking buffer and incubated with the immobilized HER2 at 37°C for 1 h. Without discarding the non-His-tagged IgG, 2 µg/mL His-tagged antibodies was immediately added to all wells. Incubation continued at 37°C for 30 min with agitation. After washing, the subsequent binding of His-tagged IgG protein was measured with anti-his-HRP (Genscript, A00612) using ELISA.

b. **Why mouse antibodies were tested via ELISA, and rabbit – via flow cytometry?**

Both ELISA and flow cytometry can be employed to determine antibody epitopes. Given the limited number of mouse antibodies (only two in this study), ELISA was selected for its practical advantages in small-scale analysis. Conversely, the high quantity of rabbit antibodies made flow cytometry a more streamlined approach for large-scale screening.

In the revised manuscript, we have supplemented epitope binning experiments for both mouse and rabbit antibodies via flow cytometry. Because the variable regions of the two mouse antibodies were also cloned into a human IgG1 backbone, they could not be detected with human secondary antibodies to assess binding to trastuzumab/pertuzumab-saturated MCF7 cells. Thus, we directly conjugated both the mouse and rabbit antibodies with AF647 fluorescent dye using NHS ester chemistry.

c. **Flow cytometry competitive experiment was not described adequately in the Results section. If the authors used transient transfection, how was the efficiency of the transfection tested? What was the concentrations for all used antibodies? How were these concentrations determined?**

Since we can detect endogenous HER2 protein expression in Expi293 cells, we didn’t transiently transfect HER2 plasmid for flow cytometry assay.

We titrated the required concentrations of trastuzumab and pertuzumab to saturate HER2 on Expi293 cells, and found that neither 10 μg/mL nor even 20 μg/mL antibodies could fully saturate all HER2 proteins on the cell surface (Figure 3). Given that Expi293 is neither a breast cancer cell line nor an established model for cell-surface HER2 expression, we switched to MCF7 breast cancer cells (with low HER2 expression) for epitope binning in the revised manuscript.

Fig. 3. The concentration of trastuzumab and pertuzumab required to saturate HER2 on Expi293 cells was determined using flow cytometry.

We titrated trastuzumab and pertuzumab on MCF7 cells to determine saturation levels and found that 100 nM sufficiently saturated all HER2 proteins on the MCF7 cell surface (Figure 4 in this response letter, same with Figure 1B in the revised manuscript). In subsequent flow cytometry experiments, cells were blocked with 267 nM (40 μg/mL) trastuzumab and pertuzumab, followed by assessment of 267 nM (40 μg/mL) mouse and rabbit antibody binding.

Fig. 4. The concentration of trastuzumab and pertuzumab required to saturate HER2 on MCF7 cells was determined using flow cytometry.

Although 10 μg/mL antibodies did not achieve complete HER2 saturation on Expi293 cells, the results remain biologically informative. Based on the Expi293 epitope binning data, we selected one negative control antibody (r35) and six antibodies (r3, r4, r5, r29, r36, r40) with epitopes distinct from trastuzumab/pertuzumab for validation in MCF7 cells. The binding profiles ultimately aligned with results from Expi293 cells (Figure 1E, Figure S2C in the revised manuscript).

**Inhibition breast cancer cell proliferation assay**

d. **There was no figure that allows to compare the levels of HER2 expression in three cell line used. Binding dose-response curves with tested antibodies (including clinical references trastuzumab and pertuzumab) are required for each of the cell lines before the other experiments are performed. The data presented on Figure 3 are not sufficient do describe the tested antibodies binding abilities.**

We didn’t compare the levels of HER2 expression in the three cell lines we used because we can get the expression information from these aspects. First, the Figure 1 of this paper (doi: 10.1155/2012/486568) and Table 1 and Supplementary Table 4 of the paper (doi: 10.1038/s41467-023-37029-3) also can give the conclusion that HER2 protein level is lower in MCF7 cells than BT-474 and SKBR3 cells.

Figure 1 of the paper (doi: 10.1155/2012/486568)

Table 1 and Supplementary Table 4 of the paper (doi: 10.1038/s41467-023-37029-3)

Fig. 5. Binding profiles of trastuzumab, pertuzumab, m66 and r40 to three tumor cell lines expressing HER2.

Further, the MFI of the flow cytometry analysis also revealed significantly lower HER2 protein levels in MCF7 cells compared to both BT-474 and SK-BR-3 cells (Figure 5 in this response letter, same with Figure 3 in the revised manuscript).

e. **What concentration of the tested antibodies did induce the inhibitory effect and what were the EC_50_ s for the inhibition? What criteria did the authors use to compare the inhibitory effect for all 4 antibodies? It looks that the effective concentrations for the tested antibodies were way too high (100 µg/mL equal 670 nM).**

We adjusted antibody concentrations using threefold serial dilutions starting from 100 nM (15 ug/mL) and re-assessed proliferation across three breast cancer cell lines. These results are presented in Figure 2A and 2B of the revised manuscript. And the EC₅₀ values were determined by fitting dose-response curves from CCK-8 assays in these three breast cancer cells (Table 1).

|  | **T** | **P** | **m66** | **r40** | **T+P** | **T+P+r40** |
| --- | --- | --- | --- | --- | --- | --- |
| **BT-474** | 5.04 | 2.217 | 2.15 | 2.277 | 1.984 | 1.51 |
| **SK-BR-3** | 3.099 | 2.297 | Unstable | 2.66 | 7.899 | 1.585 |
| **MCF7** | 0.02602 | 1.041 | 1.436 | 0.00002711 | 1.075 | 1.122 |

Table 1. Antibody EC₅₀ values were determined by fitting dose-response curves from CCK-8 assays in breast cancer cells

The EC_50_ s of trastumab, pertuzumab, m66 and r40 with immobilized HER2 ECD protein are 4.837 ng/mL (32.247 pM), 6.954 ng/mL (46.36 pM), 13.59 ng/mL (90.6 pM) and 4.146 ng/mL (27.64 pM) by ELISA analysis (Figure 6).

Fig. 6. EC₅₀ of selected antibodies were determined by ELISA analysis.

f. **Why the data presented in a different manner for different cells (Figure 2).**

Figure 2 has been updated in the revised manuscript. We initially assessed the effects of trastuzumab, pertuzumab, mouse monoclonal antibody m66, and six rabbit monoclonal antibodies (1-100 nM) on BT-474 cell proliferation. Combinations including trastuzumab + pertuzumab and triple antibodies (trastuzumab + pertuzumab + r40) were also evaluated. Antibodies and combinations showing inhibitory activity were subsequently validated in SK-BR-3 and MCF7 cells. These results are presented in Figure 2A and 2B of the manuscript.

g. **Why NRG1-independent and NRG1-dependent proliferation experiment was performed only in MCF7 cells?**

In the revised manuscript, the effect of antibodies on cell proliferation under ligand-dependent conditions was not assessed. We assessed antibody-mediated proliferation inhibition under ligand-independent conditions across all three breast cancer cell lines. The r40 antibody demonstrated inhibitory effects comparable to or greater than trastuzumab and pertuzumab, and the inhibitory effect of m66 was weaker than r40.

The triple combination (trastuzumab + pertuzumab + r40) exhibited inhibition equivalent to trastuzumab + pertuzumab in MCF7 cells, while showing superior efficacy at concentrations below 1 nM in both BT-474 and SK-BR-3 cells. These results are presented in Figure 2A and Figure 2B of the manuscript.

3. **HER2 signaling**
   a. **Again, what was the rationale to use one or another cell line in different experiments? The authors either need a good rationale to use one cell liner, or use all three and show the results for all three.**

In the revised manuscript, we assessed the effects of individual antibodies, trastuzumab + pertuzumab dual combination, and triple combination (trastuzumab + pertuzumab + r40) on HER2 signaling pathways across three cell lines under three ligand conditions: ligand-free, EGF-stimulated, and NRG1-stimulated. These results are presented in Figure 4 and Figure 5 of the manuscript.

b. **What was a rationale for using 50µg/mL of the antibodies? This is unusually high concentration for an antibody, especially for the antibodies with the reported high affinity – in this manuscript, the novel antibodies exhibit sub-nanomolar (r40) and low nanomolar (m66) affinity.**

We tested four antibody concentrations (5, 10, 25, and 50 μg/mL) in MCF7 cells and observed minimal variation in HER2 protein downregulation across these doses (Figure 4A in the revised manuscript). As concentrations between 10-50 μg/mL are standard in published literatures, we selected 10 μg/mL for subsequent Western blotting experiments. Some of the previously published literatures are listed as follows.

c. **Western blot images need quantification.**

Thanks for the reviewer's comment. In the revised manuscript, we have updated the Western blot analyses (Figure 4 and Figure 5), band intensity differences were sufficiently clear and interpretable， so we didn’t use quantification software.

d. **Interestingly, r40 affects the AKT pathway in SK-BR-3 cell line but not in BT-474 (Figure 4). Is it a cell-line dependent effect, or there is a rational reason for it?**

Thanks for the reviewer's comment. We observed that r40 reduced p-AKT (S473) levels in BT-474 cells, though this reduction was less pronounced than in SK-BR-3 cells. In the revised manuscript, we have updated the ligand-independent Western blotting results (Figure 4 in the revised manuscript). The r40 antibody did not reduce pAKT (S473) phosphorylation in MCF7 cells, whereas pAKT (S473) levels were significantly reduced in BT-474 and weakly reduced in SK-BR-3 cells. So we think it is a cell-line dependent effect.

e. **For the ligand-dependent signaling, r40 affected AKT pathway in BT-474, and, to least extent, in SK-BR-3 but not in MCF7. Did the authors consider this difference?**

In the revised manuscript, we stimulated three breast cancer cell lines with EGF and NRG1 ligands separately.

Under EGF stimulation: r40 exhibited the most pronounced reduction of p-AKT (S473) in BT-474 cells. The triple antibody combination (trastuzumab + pertuzumab + r40) reduced p-AKT (S473) more effectively than the dual combination in both BT-474 and SK-BR-3 cells (Figure 5A-5C of the revised manuscript).

Under NRG1 stimulation: r40 showed weaker activity. The triple combination demonstrated superior efficacy (Figure 5D-5F of the revised manuscript).

These differential responses are attributed to cellular heterogeneity across the different kinds of cells.

f. **Finally, pharmacological inhibitors of the AKT and ERK are needed as controls for these type of the experiments**

We used AKT inhibitor LY294002 and ERK inhibitor U0126 for the kinase assay in MCF7. As shown below, the two inhibitors clearly decreased the levels of p-AKT (S473) and p-ERK (T202/Y204) (Figure 7).

Fig.7. Effects of ligands and small-molecule inhibitors on the HER2 signaling pathway in MCF7 cells

5. Review Comments to the Author

Please use the space provided to explain your answers to the questions above. You may also include additional comments for the author, including concerns about dual publication, research ethics, or publication ethics. (Please upload your review as an attachment if it exceeds 20,000 characters)

Reviewer #1: These authors sought to identify a novel antibody specific to HER2-expressing cancers that could combat treatment resistance and cancer recurrence. They found that r40 could inhibit several key pathways, including cell proliferation, by modulating several cellular pathways such as PI3K/AKT and MAPK.

I found this paper very interesting to read and I congratulate the authors on their novel work. My main comments are around sentence structure and grammatical/spelling errors. I found that the authors would often switch between past and present tense when discussing their results. For clarity and readability, it is important to pick one tense and stick to it throughout. I also found that the authors sometimes abbreviated terms with the acronym coming before the term. For example:

"The EGFR (human epidermal growth factor receptor) family comprises four members..."

However, this should read:

"The human epidermal growth factor receptor (EGFR) family comprises four members..."

Other times the authors correctly put the acronym in brackets afterwards. Please ensure this is consist across the manuscript. I have attached my revisions in a word doc. Again, I would like to congratulate the authors on a novel study that address an urgent gap in clinical care for patients with HER2 cancers.

We thank the reviewer for evaluating our manuscript and the constructive comments that are very helpful for improvement of the manuscript. We have clearly checked our manuscript and the placement of abbreviations relative to full terms has been standardized. And also we made several changes on the tense, as shown in page 5 of the revised manuscript “indicating that the binding affinity of m66 was weaker than that of pertuzumab”, “is” has been changed to “was”; “The superimposed structure of HER2-HER3-NRG1 (PDB: 7MN5) and HER2-r40-trastuzumab-pertuzumab structure showed that the epitope of antibody r40 was opposite to the dimerization interfaces of HER2” in page 10 of the revised manuscript, “indicates” has been corrected to “showed” and “is” has been corrected to “was”.

Reviewer #2: This manuscript demonstrated the selection of mouse antibody m66 and rabbit antibody r40, which target distinct sites on HER2 that are different from those bound by trastuzumab and pertuzumab. It also examined their effects on inhibiting HER2-dependent signaling pathways.
Unfortunately, this manuscript does not include data from cancer cell proliferation inhibition assays using these antibodies in combination. As a result, the insights gained from the structural analysis of the antibody-HER2 complexes are not fully utilized, diminishing the impact of the report.

We thank the reviewer for evaluating our manuscript and the constructive comments that are very helpful for improvement of the manuscript. We have supplemented the effects of the triple-antibody combination on cell proliferation and the HER2 signaling pathway in Figure 2，Figure 4 and Figure 5 of our revised manuscript.

Additionally, while the experiments were conducted properly and yielded new findings, the results were presented in a manner that could lead to erroneous conclusions. Consequently, it has been determined that, in its current form, this manuscript is insufficient for publication.

Throughout the manuscript, the initial citations of figures are not appropriately placed. Figures should be referenced within specific explanatory contexts. For example, Fig 1A should be cited in the sentence: "As expected, each non-His-tagged IgG significantly blocked...".
Similar citation issues appear throughout the manuscript.
We thank the reviewer for evaluating our manuscript and the constructive comments that are very helpful for improvement of the manuscript. We have clearly checked our manuscript and made several changes on this. “As expected, each non-His-tagged IgG significantly blocked the binding of its own His-tagged IgG (Fig 1A)”.

There are numerous parts of the text that are insufficiently explained. The authors should seek feedback from colleagues by having them review the manuscript.
Below are some examples:

- In the Abstract and Introduction sections, both r40 and m66 should be mentioned, along with a clarification that they are monoclonal antibodies derived from rabbit and mouse, respectively.

We have changed the sentence in the Abstract, "Here, we describe a novel monoclonal antibody targeting HER2, termed r40 " to " Here, we describe two novel HER2-targeting monoclonal antibodies, m66 (mouse-derived) and r40 (rabbit-derived), which inhibit breast cancer cell proliferation in vitro" in the revised manuscript.

- The sentence, "The binding kinetics of trastuzumab, pertuzumab…" should specify the binding target, possibly HER2.

We have changed the sentence: "The binding kinetics of trastuzumab, pertuzumab, m66 and r40 were evaluated by surface plasmon resonance (SPR)" to "The binding kinetics of trastuzumab, pertuzumab, m66 and r40 with HER2 were evaluated by surface plasmon resonance Imaging (SPRi)" in the page 7 in the reviesed manuscript.

- A reference for PDB entry #7MN5 should be provided.

We have provided the reference (2021, Nature) for PDB #7MN5 in the page 10 in the reviesed manuscript.

- Figure legends should provide more specific details to allow the experiments to be understood without referring to the Materials and methods section.

We have provided more detailed figure legends in the revised manuscript, for example the legend of Fig 1B is as follows, “Saturating concentrations of trastuzumab and pertuzumab for binding to HER2 on live MCF7 cells were determined. MCF7 cells were incubated with trastuzumab and pertuzumab (0-400 nM) for 30 min, then stained with AF647-conjugated goat anti-human secondary antibody for flow cytometry analysis.” The legend of Fig 4A is as follows, “Immunoblots assessing HER2 signaling upon treatment with 5, 10, 25, 50 μg/mL (33.3, 66.7, 166.7, 333.3 nM) antibodies for 24 h in MCF7. Cell lysates were immunoblotted to detect p-AKT (Ser473), total AKT, p-ERK (T202/Y204), total ERK, HER2, GAPDH.”

This manuscript requires extensive proofreading, as it contains numerous spelling mistakes and typographical errors. Ensure that the co-authors thoroughly review the content.

We thank the reviewer for evaluating our manuscript and the constructive comments that are very helpful for improvement of the manuscript. We have clearly checked our manuscript and made several changes on this.

For example, in the sentence: "The final structural models were built with the HER2-trastuzumab-...", the reference for PDB entry #60GE may be [Ref. 10] rather than [Ref. 25].

Thanks for the reviewer's comment, we have changed the reference in the revised manuscript.

About the Figures:

Fig 2C: There is no statistically significant difference between r40 and trastuzumab in the NRG1 ligand treatment of MCF7 cells. Therefore, statements such as "Antibody r40 shows a more potent effect than trastuzumab..." should be avoided to prevent misinterpretation.

Indeed, the observed differences were not sufficiently pronounced. Consequently, we have discontinued this figure in the revised manuscript. Instead of reassessing proliferation under ligand-dependent conditions, we evaluated cell viability across three cell lines following 4 d treatment with antibody concentrations ranging from 0 to 100 nM in Figure 2A and Figure 2B of the revised manuscript.

Fig 3: The horizontal axes should be aligned to facilitate vertical comparisons, as demonstrated in Fig 1B. Additionally, the peak intensity of r40 in BT-474 appears to be very low. What could be the possible explanation?

We thank the reviewer for this query. The revised manuscript contains an updated version of this figure (see revised Figure 3). As the variable regions of m66, trastuzumab, and pertuzumab were all cloned into a human IgG1 backbone, these antibodies - along with the human IgG isotype control - were detected using anti-human fluorescent secondary antibodies. Conversely, r40 was constructed with a rabbit IgG backbone and was therefore detected with anti-rabbit secondary antibodies alongside its rabbit IgG isotype control.

Figs 4 and 5: Blots should be digitally quantified and, if possible, represented as graphs. Of course, images of the blots should be incorporated into the graphs. Ideally, statistical analysis should also be conducted, and the discussion should be based on those results.

Thanks for the reviewer's comment, In the revised manuscript, we have updated the Western blot analyses (see Figure 4 and 5), band intensity differences were sufficiently clear and interpretable, so we didn’t use quantification software.

Fig 6: It would be better to adjust the display order of the figures. Following the sequence in the Results section, the results for m66 and r40 should be presented separately for each, in the order of trimer, tetrameric complex, and superimposed structure.

Thanks for the reviewer's comment, we have sepetated the results of m66 and r40 in Figure 6 in the revised manuscript.

Reviewer #3: A nice in vitro study of selecting a new antibodies to HER2. Study is methodologically sound, data are well presented and manuscript as a whole is written in logical manner. Cryo EM part of study is especially commendable.

Thanks for the reviewer's comment.
