# Supplementary material for: Inhibition of HER2 signaling and breast cancer cell growth with a novel antibody targeting HER2 ECD III/IV
Source: PLoS One. 2026 Jan 15;21(1):e0338127. doi: 10.1371/journal.pone.0338127 (PMC12806845; doi:10.1371/journal.pone.0338127)
Supplement: Attachment — Submitted filename: Response_to_Reviewers_auresp_2.docx [file pone.0338127.s013.docx]

Journal Requirements:

If the reviewer comments include a recommendation to cite specific previously published works, please review and evaluate these publications to determine whether they are relevant and should be cited. There is no requirement to cite these works unless the editor has indicated otherwise. 

Additional Editor Comments:
There are a few discrepancies between the dose response curves and tabulated EC_50_ values (Fig. 2 and 6). Please check the numbers, values etc to make sure there is a correspondence in the data.

Fig. 1. The ELISA EC_50_ values as presented in GraphPad Prism 9 software.

Fig. 2. Determination of EC₅₀ values for trastuzumab, pertuzumab, m66, m75, and r40 binding to immobilized HER2 ECD by ELISA.

Thank you for the kind reminder. We have checked the values in the GraphPad Prism 9 (Fig. 1). The antibody concentrations used were 2^-11^-2^3^ ng/μL, with log values ranging from -11 to 3. We have also corrected the unit in corresponding table from "ng/mL" to "ng/μL" and “nM” (Fig. 2). Based on the ELISA analysis, the EC_50_ values for trastuzumab, pertuzumab, m66, m75 and r40 binding to immobilized HER2 ECD are as follows: 0.004837 ng/μL, 0.006954 ng/μL, 0.01359 ng/μL, 0.009973 ng/μL and 0.004146 ng/μL (Fig. 2).

Expi293 cells do not express HER2 naturally, please clarify the experimental details and conclusions. Regarding the saturation concentration experiments, ususally the EC_50_ binding values are considered to be saturating.

Fig.3. HER2 RNA levels in MCF7, HEK293, BT-474 and SK-BR-3 cells based on The Human Protein Atlas.

Thanks for the editor's comment. According to data from the Human Protein Atlas, HER2 RNA expression levels are comparable between MCF7 and HEK293 cell lines, with nTPM values of 47.1 and 36.4, respectively (Fig. 3). In contrast, BT-474 and SK-BR-3 cells show substantially higher HER2 expression, with nTPM values of 1979.6 and 2448.4, respectively (Fig. 3).

We initially cloned the full-length HER2 protein and expressed it in Expi293 cells to evaluate antibody binding. However, we observed that untransfected Expi293 cells also exhibited binding to the HER2 antibodies. Hence, we proceeded with subsequent experiments without transfection.

Fig.4. Flow cytometry analysis of rabbit anti-HER2 antibody binding compared to isotype control in Expi293 cells

As shown in Fig. 4, multiple rabbit monoclonal antibodies bound to untransfected Expi293 cells, with fluorescence intensities ranging from 10^3^ to 10^4^, compared to the rabbit IgG isotype control. Consistent with this, Fig. 3 of the revised manuscript shows that HER2-high-expressing BT-474 and SK-BR-3 cells exhibited fluorescence intensities around 10^5^, while HER2-low-expressing MCF7 cells showed values between 10^3^ and 10^4^. These results demonstrate that Expi293 cells express low-levels of HER2 protein on the cell surface.

Although we detected low levels of HER2 protein expression on the surface of Expi293 cells via flow cytometry, this cell line is not a breast cancer model and is therefore considered less relevant in this field of research. Shall we omit the Expi293-based epitope validation data from the revised manuscript? Instead, we have focused on the results obtained from the breast cancer cell line MCF7 for epitope characterization.

The Western blots images must be quantified and normalized to the levels of house keeping proteins (in particular, because the levels of the latters are not even).

We appreciate the editor’s comment. In response, we have quantified the Western blot signals and normalized them as follows: HER2 levels were normalized to GAPDH, p-AKT (S473) to total AKT, and p-ERK (T202/Y204) to total ERK. The normalized quantitative data are now provided in the revised Fig. 4 and Fig. 5.

It is not quite clear why the authors didn't see the dose response effect when tested HER2 signaling - it may indicate that the effect is not induced by the antibodies. The authors should re-test the dose response to cover the bigger concentration range (i.e. 1: 5 dilution, 6-8 points). Could it also be that the effect is time-dependent, and was not tested at the optimal time point? Also, why the levels of HER2 are so different for untreated MCF-7 (Fig.4)?

Thanks for the editor's comment. The reduced HER2 signal in the control sample of the 5 μg/mL antibody treatment group in the original Fig. 4A is likely attributable to an edge effect. This occurred because 14 samples were loaded on a single SDS-PAGE gel, and the large membrane size combined with the sample’s position at the outer edge may have led to diminished transfer efficiency or uneven antibody binding.

In Fig. 4B of the publication (doi: 10.4161/mabs.28786), dose-response assays were performed in MCF7 cells using trastuzumab and pertuzumab across a broad concentration range (0.01 to 50 nM, serially diluted at 1:2 or 1:3 ratios) under 0.5 nM NRG1 stimulation for 10 min. As demonstrated, both antibodies at 50 nM (7.5 μg/mL) markedly reduced the phosphorylation levels of AKT and MAPK.

Fig. 4B of the paper (doi: 10.4161/mabs.28786)

The MCF7 cell line is classified as an HR+ (ER+/PR+), HER2-low breast cancer model. This indicates that its basal level of HER2 protein expression is relatively low, with minimal formation of HER2 homodimers or heterodimers (e.g., with EGFR or HER3). Consequently, the basal activity of the PI3K/AKT and MAPK signaling pathways in these cells is likely not primarily driven by HER2. Therefore, treatment with HER2 antibody alone is unlikely to produce significant inhibitory effects on these signaling pathways，as demonstrated by our experiment (Fig. 4A of the revised manuscript). The addition of EGF or NGR1 is intended to "create" a sensitive system enabling the evaluation of HER2 antibody efficacy (Fig. 5A, 5D of the revised manuscript).

Therefore, using MCF7 cells in the absence of ligand stimulation to test antibody concentration gradients was not ideal. Accordingly, in the revised Fig. 4A of the manuscript, we have retained only the Western blot data for the 10 μg/mL antibody concentration.

In previously published literatures investigating the effects of trastuzumab and pertuzumab treatment on signaling pathway alterations in breast cancer cell lines via Western blot, the antibody concentrations commonly used by researchers range from 7.5 μg/mL to 15 μg/mL, with a treatment duration of 24 hours in most cases (Fig. 5). Therefore, we selected a concentration of 10 μg/mL to treat the three breast cancer cell lines and harvested the samples after 24 hours.

Fig.5. Previously published literatures investigating the effects of trastuzumab and pertuzumab treatment on signaling pathway alterations in breast cancer cell lines via Western blot

Figure 2 is confusing. What is plotted on the X axis - concentration of the antibody or the logarithm of concentration?

Thanks for the editor's comment. We would like to confirm that the X-axis represents antibody concentration (μg/mL) with a logarithmic (log₁₀) scale, as shown in Fig. 6.

Fig.6. The Format Axes as presented in GraphPad Prism 9.

[Note: HTML markup is below. Please do not edit.]

Reviewers' comments:

Reviewer's Responses to Questions

**Comments to the Author**

1. If the authors have adequately addressed your comments raised in a previous round of review and you feel that this manuscript is now acceptable for publication, you may indicate that here to bypass the “Comments to the Author” section, enter your conflict of interest statement in the “Confidential to Editor” section, and submit your "Accept" recommendation.

Reviewer #1: All comments have been addressed

Reviewer #2: (No Response)

2. Is the manuscript technically sound, and do the data support the conclusions?

The manuscript must describe a technically sound piece of scientific research with data that supports the conclusions. Experiments must have been conducted rigorously, with appropriate controls, replication, and sample sizes. The conclusions must be drawn appropriately based on the data presented.

Reviewer #1: Yes

Reviewer #2: Yes

3. Has the statistical analysis been performed appropriately and rigorously?

Reviewer #1: Yes

Reviewer #2: Yes

4. Have the authors made all data underlying the findings in their manuscript fully available?

The [PLOS Data policy](http://track.editorialmanager.com/CL0/http:%2F%2Fwww.plosone.org%2Fstatic%2Fpolicies.action%23sharing/1/010f0198efaafde1-e345a1f4-8fee-4686-8b7e-37d903c7b577-000000/oUHqmBajOhruIKcmgc7vwgUxFF-cmqsbZdGXE24zRis=226) requires authors to make all data underlying the findings described in their manuscript fully available without restriction, with rare exception (please refer to the Data Availability Statement in the manuscript PDF file). The data should be provided as part of the manuscript or its supporting information, or deposited to a public repository. For example, in addition to summary statistics, the data points behind means, medians and variance measures should be available. If there are restrictions on publicly sharing data—e.g. participant privacy or use of data from a third party—those must be specified.

Reviewer #1: Yes

Reviewer #2: Yes

5. Is the manuscript presented in an intelligible fashion and written in standard English?

PLOS ONE does not copyedit accepted manuscripts, so the language in submitted articles must be clear, correct, and unambiguous. Any typographical or grammatical errors should be corrected at revision, so please note any specific errors here.

Reviewer #1: Yes

Reviewer #2: No

6. Review Comments to the Author

Please use the space provided to explain your answers to the questions above. You may also include additional comments for the author, including concerns about dual publication, research ethics, or publication ethics. (Please upload your review as an attachment if it exceeds 20,000 characters)

Reviewer #1: All feedback from previous reviews was successfully implemented. I found some spelling/grammatical errors in this round, and have highlighted these (green) in the manuscript. Other than this, I found this work to be very interesting and well communicated, and believe it is ready for publication once these errors have been corrected. Congratulations to the authors

Reviewer #2: I acknowledge the addition of new data in the revised manuscript, which contributes to strengthening the overall conclusions.
However, the data presentation remains confusing and poorly structured, making the manuscript, in its current form, unsuitable for publication.
To improve clarity and impact, revisions are needed.
The following are examples of suggestions for improvement.

1. Antibody screening should be described in the Materials and Methods section.
- To Supplemental data: ELISA (no data provided?), pull-down assay (Response Letter Fig. 1), and FCM using Expi293 cells (Fig. S2A)

We thank the reviewer for the valuable suggestion. In response, we have prepared the corresponding figures (Fig. 7 and Fig. 8) and detailed experimental protocols for both the ELISA and pull-down assays. Would it be appropriate to include descriptions of these assays in the Materials and Methods section? Additionally, would you recommend including these two figures in the Supplementary Materials?

ELISA

HER2 ECD protein was diluted to 0.2 µg/mL in PBS and coated overnight at 4°C. After blocking, the medium of recombinant expressed IgG was diluted 1:100 in blocking buffer (PBST containing 3% BSA) and incubated for 1 h. Following washing steps, bound IgG was detected using the HRP-conjugated goat anti-human IgG1 Fc antibody (Millipore, AP113P) for trastuzumab, or HRP-conjugated goat anti-rabbit IgG (H+L) antibody (Abclonal, AS014) for rabbit monoclonal antibodies.

Fig. 7. ELISA Characterization of rabbit monoclonal antibodies.

Pull-down

A 20 μL volume of a 20% slurry rProtein A/G magnetic beads (SMART, SM015010) was pre-equilibrated with washing buffer (PBS containing 0.1% CHAPS) and incubated with 10 μg of antibody at room temperature for 1 h. After three washes with washing buffer, the beads were divided into two equal aliquots. One aliquot was used as the negative control; the other was incubated with 20 μg of HER2 ECD protein for 1 h at room temperature, washed three times with washing buffer, followed by SDS-PAGE analysis.

Fig. 8. Characterization of rabbit monoclonal antibodies by pull-down assay.

- To M&M: T&P saturation data (Fig. 1B) should be explained as the basis for determining T&P concentrations at 267 nM.

We thank the reviewer for the valuable suggestion. We have added “As shown in the saturation curve, the saturating concentrations for both antibodies were determined to be approximately 133.3 nM (Fig. 1B)” at page 6 of the revised manuscript. And “As expected, the binding of AF647-conjugated trastuzumab and pertuzumab was abolished in MCF7 cells pre-saturated with 267 nM (twice the saturation concentration) of unlabeled trastuzumab and pertuzumab (Fig. 1C)" in the following paragraph.

2. The Results section should describe the selection process in comparison to T&P.
- Data: mouse antibody ELISA (Fig. 1A), rabbit antibody FCM using Expi293 cells (Fig. S2B), and FCM using MCF7 cells (Fig. 1C–E*, S2C)

We thank the reviewer for the valuable suggestion.

For mouse antibody ELISA (Fig. 1A), the selection process in comparison to T&P is described as follows, “Furthermore, pre-saturation with m66 or m75 did not impair trastuzumab binding, suggesting that both antibodies bind to epitopes distinct from that of trastuzumab. Interestingly, the binding of m66 was impaired by pertuzumab, whereas pertuzumab binding was not affected by m66, indicating that m66 has lower binding affinity than pertuzumab and may bind an overlapping or adjacent epitope (Fig. 1A). Antibody m75 reduced the binding of pertuzumab, and conversely, pertuzumab impaired the binding of m75 (Fig. 1A). These findings suggest that m66 and m75 recognize epitopes distinct from that of trastuzumab, yet likely overlap with the binding site of pertuzumab.”

For rabbit antibody FCM using Expi293 cells (Fig. S2B), the selection process in comparison to T&P is described as follows, “To characterize rabbit antibody epitopes through competitive binding, we pre-incubated Expi293 cells with both trastuzumab and pertuzumab at the concentration of 10 μg/mL (66.7 nM). The cells were then incubated with individual rabbit antibodies and analyzed by flow cytometry to evaluate simultaneous binding. For the antibodies that did bind, pre-incubation with trastuzumab and pertuzumab did not reduce the mean fluorescence intensity (MFI) compared to the control (cells incubated with the rabbit antibody alone). This indicated that the binding of these rabbit antibodies to HER2 did not compete with the epitopes targeted by trastuzumab and pertuzumab. Based on these results, we concluded that six rabbit antibodies (r3, r4, r5, r29, r36, and r40) might recognize epitopes distinct from those of trastuzumab and pertuzumab (S2B Fig).”

For FCM using MCF7 cells (Fig. 1C–D), the selection process in comparison to T&P is described as follows, “Subsequently, trastuzumab, pertuzumab, m66, and m75 were conjugated with NHS-AF647 dye. As expected, the binding of AF647-conjugated trastuzumab and pertuzumab was abolished in MCF7 cells pre-saturated with unlabeled trastuzumab and pertuzumab (Fig. 1C). Similarly, the binding of both m66-AF647 and m75-AF647 was blocked exclusively by pre-saturation with unlabeled pertuzumab, but only minimally affected by trastuzumab saturation (Fig. 1D). These results further support that m66 and m75 recognize epitopes overlapping with that of pertuzumab, consistent with the ELISA data (Fig. 1A). Notably, increased m66-AF647 binding was observed in cells pre-saturated with trastuzumab, suggesting that trastuzumab binding induces conformational changes in HER2 that enhance the accessibility or affinity for m66 (Fig. 1D).”

For FCM using MCF7 cells (Fig. 1E), the selection process in comparison to T&P is described as follows, “The epitope recognition profiles of r3, r4, r5, r29, r36, and r40 were subsequently characterized and compared with those of trastuzumab and pertuzumab using the low-HER2-expressing MCF7 cells (Fig. 1E). Pre-saturation of HER2 with trastuzumab and pertuzumab did not impede the subsequent binding of AF647-conjugated r3, r4, r5, r29, or r40, suggesting that their epitopes are distinct from those targeted by these two therapeutic antibodies (Fig. 1E). In contrast, r36 exhibited enhanced binding signal on cells pre-saturated with trastuzumab and pertuzumab, suggesting that therapeutic antibody binding induces conformational changes in HER2 that facilitate r36 recognition (Fig. 1E).”

*The r35 data in Fig. 1E is not required.

Thanks for the reviewer's comment. The r35 data has been removed in Fig. 1E in the revised manuscript.

*Legends should be standardized to “non-saturated cells (or MCF7)” and “T&P-, T-, or P-saturated cells (or MCF7)”
Thanks for the reviewer's comment. Legends have been standardized to “non-saturated cells” and “T&P-, T-, or P-saturated cells” in the revised Fig. 1C-1E.

3. Emphasize the results of the CCK-8 assay (Fig. 2A, B*) and HER2 signaling analysis (Figs. 4, 5**).

*Including EC50 values (Response Letter Table 1) would be helpful, although the graph curves do not appear to support reliable EC50 estimation.

We appreciate the reviewer’s suggestion. In the revised Supplementary Fig. S2C, we have incorporated the EC₅₀ values of the relevant antibodies—determined via CCK-8 assay across all three cell lines. These data provide further quantitative support for the anti-proliferative effects described in the study.

**Consider placing the HER2 blot at the top of the figure panel

Thanks for the reviewer's suggestion. We have placed the HER2 blot at the top of the figure panels in the revised Fig. 4 and Fig. 5.

4. Summarize the characteristics of m66 and r40.
- To Results or Supplemental data: SPR (Fig. 2C), FCM (Fig. 3*)

We thank the reviewer for this suggestion. We have revised the section describing the SPRi results (Fig. 2C), in which we summarized the characteristics of m66 and r40 and compared them with trastuzumab and pertuzumab, as follows:

The binding kinetics of trastuzumab, pertuzumab, m66 and r40 to HER2 were evaluated by Surface Plasmon Resonance Imaging (SPRi). Notably, r40 exhibited high affinity (*K_D_* = 0.279 nM), comparable to that of trastuzumab (*K_D_* = 0.283 nM). In contrast, m66 showed the modest binding affinity (*K_D_* = 86.4 nM), which was considerably lower than that of both r40 and the therapeutic antibodies trastuzumab and pertuzumab (*K_D_* = 2.3 nM) (Fig. 2C). These results indicate that r40 possesses excellent HER2-binding properties, whereas m66 demonstrates relatively weak binding under the tested conditions.

We have also revised the section describing the FCM (Fig. 3) results as follows:

We next evaluated the binding capacity of m66 and r40 to the endogenous HER2 protein in breast cancer cells using flow cytometry. The m66 antibody, which incorporates variable regions on a human IgG1 backbone, can be detected using an anti-human secondary antibody, similar to trastuzumab and pertuzumab (Fig. 3A-3C). In contrast, r40 is fully rabbit-derived and therefore requires a rabbit-specific secondary antibody for detection. Importantly, m66 showed binding intensity comparable to that of trastuzumab and pertuzumab. BT-474 and SK-BR-3 cells showed a significantly greater MFI than MCF7 cells, consistent with their higher HER2 expression levels (Fig. 3A-3C).

*Cite the paper (doi: 10.1155/2012/486568, Response Letter Fig. 1) to support the low HER2 expression in MCF-7 cells.

We thank the reviewer for this suggestion. The paper (doi: 10.1155/2012/486568) was published in *Evidence-Based Complementary and Alternative Medicine*, which was removed from the SCIE database in March 2023. Therefore, we cite a 2023 *Nature Communications* study (doi: 10.1038/s41467-023-37029-3), which explicitly identifies MCF7 as a HER2-low breast cancer cell line. The citation is included in the following sentence, “To map antibody epitope via flow cytometry, we used the low-HER2-expressing breast cancer cell line MCF7.”

*Include units and labels on the y-axis?

The Y-axis represents cell count. Since the original cell numbers differed, leading to variations in peak heights, we adjusted the Y-axis mode to "Modal" in FlowJo software to normalize the peak heights (Fig. 8). The Y-axis is labeled as "Normalized to Mode" in the Fig. 3 in the revised manuscript.

Fig.8. Graph definition of FlowJo 10.8.1 software.

We have also added “Genometric mean” to our figures as the Supplementary Fig. 5d of this paper (10.1038/s41467-023-37029-3).

Supplementary Fig. 5d of 10.1038/s41467-023-37029-3

5. In Fig. 6, divide the panels into A (m66 group) and B (r40 group), and label each panel with consistent headings such as:
"Cryo-EM map", "High-resolution model", "Superimposed with HER2-T-P", and "Superimposed with HER2-HER3-NRG1".

Thanks for the reviewer's comment. We have revised Fig. 6 accordingly based on the suggestions provided above.

Revised Fig. 6 of the manuscript

6. The manuscript should be carefully reviewed to ensure internal consistency across all parts. Several inconsistencies remain between the revised main text and other sections such as the Materials and Methods and figure captions.
Examples:
- The ELISA EC50 data (Response Letter Fig. 1) are not presented, yet the Materials and Methods section still describes them.

Thanks for the reviewer's comment. In the revised Supplementary Fig. 1D, we have included the EC₅₀ values of the antibody as determined by ELISA. In the Materials and Methods section, we have detailed the ELISA-based method used to determine the EC₅₀ values.

- The caption for Fig. 6 does not correspond to the actual figure content

Thanks for the reviewer's comment. The caption for Fig. 6 has been revised to “Cryo-EM maps and structures of HER2-m66-trastuzumab and HER2-r40-trastuzumab-pertuzumab”.

- The statement "When cells were pre-incubated with trastuzumab and pertuzumab, the mean fluorescence intensity (MFI) of the rabbit monoclonal antibody remained comparable to control cells incubated with the rabbit antibody alone" lacks supporting data

Thanks for the reviewer's comment. This sentence could lead to confusion, and has been revised as follows. “For the antibodies that did bind, pre-incubation with trastuzumab and pertuzumab did not reduce the mean fluorescence intensity (MFI) compared to the control (cells incubated with the rabbit antibody alone). This indicated that the binding of these rabbit antibodies to HER2 did not compete with the epitopes targeted by trastuzumab and pertuzumab.”

However, as Expi293 cells are not widely recognized for cell surface HER2 protein expression and are not commonly used in breast cancer research, shall we remove the corresponding experimental data and figures regarding HER2 antibody epitope determination in Expi293 cells from the revised manuscript?

- In Fig. 5C, the claim that "m66 inhibited p-AKT (S473) more potently than r40" is not clearly supported by the band intensity shown.

Thanks for the reviewer's comment. Based on the quantification of Western blot data, the ratios of p-AKT(S473) to total AKT for the m66 and r40 antibodies were 1.7 and 1.9, respectively. Since the difference between them was minimal, this statement has been removed from the revised manuscript.
- The term "AlphaFold2" is used in the Results section, whereas "AlphaFold" appears in the Materials and Methods section—this should be clarified

Thanks for the reviewer's comment. We have uniformly adopted "AlphaFold 2" throughout the manuscript, as this tool was used for all structural predictions in our study.

7. A thorough proofreading is required. Numerous typographical and grammatical errors are present throughout the manuscript. Please ensure that basic language editing is completed prior to submission.
Examples:
- "While antibody m75 blocked pertuzumab and pertuzumab blocked m75 (Fig. 1A)." — This sentence is grammatically incomplete and unclear.

Thanks for the reviewer's comment. We have revised this sentence to “Antibody m75 reduced the binding of pertuzumab, and conversely, pertuzumab impaired the binding of m75 (Fig. 1A).”

- "..., which explained why m66 and pertuzumab didn’t not bind HER2 simultaneously."

Thanks for the reviewer's comment. The “not” behind “didn’t” should be removed, and the sentence has been revised to “The observed inability of m66 and pertuzumab to bind HER2 simultaneously can be explained by a steric clash that would occur if both antibodies were bound.”

- "..., and then a of serial dilutions of HER2 (1-128 nM) in PBS ..."

Thanks for the reviewer's comment. We acknowledge the error in this statement. It has been corrected to: " A series of HER2 dilutions in PBS, ranging from 1 to 128 nM, underwent 285 s of association followed by 930 s of dissociation. "

- "In summary, we screened a panel of human HER2 antibodies and ..." — This sentence may give the misleading impression that the authors screened human antibodies.
To avoid confusion, it should be revised to: "a panel of (mouse and rabbit) antibodies targeting human HER2."

Thanks for the reviewer's comment. We have revised this sentence to “In summary, we screened a series of mouse- and rabbit-derived antibodies against human HER2 and identified r40 as an ECD III/IV-specific antibody that disrupted PI3K/AKT and MAPK signaling pathways and suppressed the growth of breast cancer cells”.

7. PLOS authors have the option to publish the peer review history of their article ([what does this mean?](https://track.editorialmanager.com/CL0/https:%2F%2Fjournals.plos.org%2Fplosone%2Fs%2Feditorial-and-peer-review-process%23loc-peer-review-history/1/010f0198efaafde1-e345a1f4-8fee-4686-8b7e-37d903c7b577-000000/1JipM5PWG5lYNiI1SJ-bGgmhmuN06_59oSsr3wwhxbw=226)). If published, this will include your full peer review and any attached files.


If you choose “no”, your identity will remain anonymous but your review may still be made public.


**Do you want your identity to be public for this peer review?** For information about this choice, including consent withdrawal, please see our [Privacy Policy](https://track.editorialmanager.com/CL0/https:%2F%2Fwww.plos.org%2Fprivacy-policy/1/010f0198efaafde1-e345a1f4-8fee-4686-8b7e-37d903c7b577-000000/HB97-n5Y48MXXFdWZm-3ZXBFUzVoZVFvSqWD7CUo3_Q=226).

Reviewer #1: No

Reviewer #2: No

[NOTE: If reviewer comments were submitted as an attachment file, they will be attached to this email and accessible via the submission site. Please log into your account, locate the manuscript record, and check for the action link "View Attachments". If this link does not appear, there are no attachment files.]

While revising your submission, please upload your figure files to the Preflight Analysis and Conversion Engine (PACE) digital diagnostic tool, [https://pacev2.apexcovantage.com/](https://track.editorialmanager.com/CL0/https:%2F%2Fpacev2.apexcovantage.com%2F/1/010f0198efaafde1-e345a1f4-8fee-4686-8b7e-37d903c7b577-000000/gcmbThZl1Qvwtpp0_OuXhv_JfYGs6kigynL478ApWXo=226). PACE helps ensure that figures meet PLOS requirements. To use PACE, you must first register as a user. Registration is free. Then, login and navigate to the UPLOAD tab, where you will find detailed instructions on how to use the tool. If you encounter any issues or have any questions when using PACE, please email PLOS at [figures@plos.org](mailto:figures@plos.org). Please note that Supporting Information files do not need this step.
