# Supplementary material for: Inhibition of HER2 signaling and breast cancer cell growth with a novel antibody targeting HER2 ECD III/IV
Source: PLoS One. 2026 Jan 15;21(1):e0338127. doi: 10.1371/journal.pone.0338127 (PMC12806845; doi:10.1371/journal.pone.0338127)
Supplement: Attachment — Submitted filename: Response_to_Reviewers_auresp_3.docx [file pone.0338127.s014.docx]

Reviewers' comments:

Reviewer's Responses to Questions

**Comments to the Author**

1. If the authors have adequately addressed your comments raised in a previous round of review and you feel that this manuscript is now acceptable for publication, you may indicate that here to bypass the “Comments to the Author” section, enter your conflict of interest statement in the “Confidential to Editor” section, and submit your "Accept" recommendation.

Reviewer #1: All comments have been addressed

Reviewer #2: (No Response)

2. Is the manuscript technically sound, and do the data support the conclusions?

The manuscript must describe a technically sound piece of scientific research with data that supports the conclusions. Experiments must have been conducted rigorously, with appropriate controls, replication, and sample sizes. The conclusions must be drawn appropriately based on the data presented.

Reviewer #1: Yes

Reviewer #2: Yes

3. Has the statistical analysis been performed appropriately and rigorously?

Reviewer #1: Yes

Reviewer #2: Yes

4. Have the authors made all data underlying the findings in their manuscript fully available?

The [PLOS Data policy](http://track.editorialmanager.com/CL0/http:%2F%2Fwww.plosone.org%2Fstatic%2Fpolicies.action%23sharing/1/010f019a2397a122-604d5675-3e13-4c60-8160-79d17d0c5e54-000000/v6utHnEoDtR8sdYJUtCFhZXL3PlNS3SvTQLHxh1NWJg=234) requires authors to make all data underlying the findings described in their manuscript fully available without restriction, with rare exception (please refer to the Data Availability Statement in the manuscript PDF file). The data should be provided as part of the manuscript or its supporting information, or deposited to a public repository. For example, in addition to summary statistics, the data points behind means, medians and variance measures should be available. If there are restrictions on publicly sharing data—e.g. participant privacy or use of data from a third party—those must be specified.

Reviewer #1: Yes

Reviewer #2: Yes

5. Is the manuscript presented in an intelligible fashion and written in standard English?

PLOS ONE does not copyedit accepted manuscripts, so the language in submitted articles must be clear, correct, and unambiguous. Any typographical or grammatical errors should be corrected at revision, so please note any specific errors here.

Reviewer #1: Yes

Reviewer #2: Yes

6. Review Comments to the Author

Please use the space provided to explain your answers to the questions above. You may also include additional comments for the author, including concerns about dual publication, research ethics, or publication ethics. (Please upload your review as an attachment if it exceeds 20,000 characters)

Reviewer #1: I am satisfied that all previous suggestions have been implemented into the manuscript and I have no further recommendations. I believe this manuscript is ready for acceptance. Congratulations to the authors on their interesting work.

Reviewer #2: The revised manuscript is significantly more readable and easier to follow. I would like to express my appreciation for the authors' dedicated efforts and commend their hard work.
Below are five points of concern regarding the revision.
If these are addressed, I believe the manuscript will be suitable for publication.

1. The modification of the two mouse antibodies into the human IgG format is not clearly explained. Since the purification of HER2 is described in the Materials and Methods section, it would be beneficial to include a comparable description of the antibody preparation, including whether the antibodies are in human IgG format and whether they are His-tagged.

Thank you for the kind reminder. The methodology for IgG plasmid construction and purification has been included in the Materials and Methods section of the revised manuscript as follows.

**Plasmid construction and IgG expression**

The variable heavy (VH) and light (VL) chains of trastuzumab, pertuzumab, m66, and m75 were cloned into the modified pCAG vectors containing the constant regions of human IgG1, with an HRV 3C protease cleavage site and a 9× His tag incorporated at the C-terminus of the heavy chain vector. The VH and VL regions of 39 rabbit antibodies were cloned into the similarly modified pCAG vectors carrying the rabbit IgG constant regions. Each IgG was obtained by co-transfecting of light chain and heavy chain plasmids into Expi293 cells. Following Ni-NTA affinity chromatography, His-tagged IgGs were directly eluted with imidazole, whereas non-His-tagged IgGs were released by HRV 3C protease cleavage following purification.

2. The citation of Figure 3 in the following sentence appears to be unnecessary:
"The m66 antibody, which incorporates variable regions on a human IgG1 backbone, can be detected using an antihuman secondary antibody, similar to trastuzumab and pertuzumab."

We appreciate the reviewer’s comment. We have removed the citation of Figure 3 in the revised manuscript.

3. The methods used for quantification and normalization (as explained in this Response Letter) of the Western blot bands should be included in the Materials and Methods section. Additionally, the legends for Figures 4 and 5 should clarify that the numerical values shown in the figures represent normalized quantitative data.

Thank you for the kind reminder. We have added the methods used for quantification and normalization of the Western blot bands in the Immunoblotting part of the Materials and Methods section as follows. “Western blot signals were quantified using Fiji software and normalized as follows: HER2 levels were normalized to GAPDH, p-AKT (S473) to total AKT, and p-ERK (T202/Y204) to total ERK” in the revised manuscript.

And we have added this sentence “The numerical values shown in the figures represent normalized quantitative data” in the legends of Fig. 4 and Fig. 5.

4. For the description of ECD I, III, and the III/IV interface, appropriate references should be added in the following sentences:
- In Introduction: "...ECD I plays a key role in forming a binding pocket for the dimerization arm of other EGFR family members. ECD III, located centrally within the extracellular domain, serves as a critical structural bridge between ECD II and ECD IV and interacts directly with ECD I."
- In Discussion: "...the ECD III/IV interface, a region where structural rearrangements are more likely to induce HER2 internalization."

Thank you for the kind reminder. The citations [26-28] have been placed after the following sentence in Introduction.

“Although neither ECD I nor ECD III is directly involved in HER2 dimerization, ECD I plays a key role in forming a binding pocket for the dimerization arm of other EGFR family members; ECD III, located centrally within the extracellular domain, serves as a critical structural bridge between ECD II and ECD IV and interacts directly with ECD I[26-28].”

According to this paper (doi: 10.1016/j.ctrv.2024.102826), antibodies directed against HER2 ECD I and IV are both capable of mediating HER2 internalization. The greater potency of the r40 antibody in reducing HER2 levels is more likely due to its higher affinity compared to the lower affinity of the m66 antibody. For this reason, the sentence “Alternatively, while m66 binding to ECD I may not elicit significant conformational changes, the epitope recognized by r40 lies at the ECD III/IV interface, a region where structural rearrangements are more likely to induce HER2 internalization” In Discussion has been removed in the revised manuscript.

5. In the following sentence, the citation [41] should be placed immediately after the term "AlphaFold 2":
"...the HER2-trastuzumab module and the AlphaFold 2[41] predicted structure of m66..."

Thank you for the kind reminder. The citation has been placed immediately after the term "AlphaFold 2" in the sentence.

Response to the authors' inquiry:

>> Would it be appropriate to include descriptions of these assays in the Materials and Methods section? Additionally, would you recommend including these two figures in the Supplementary Materials?

There is no issue as long as the methods and results of the ELISA and pull-down assays are properly described in the supplementary materials.
However, it is essential to cite them explicitly in the main text, specifically in the following sentence:
"...and evaluated for binding affinity using enzyme-linked immunosorbent assay (ELISA) and pull-down assays."
Furthermore, descriptions of ELISA methods, including EC50 and competitive assays, should be harmonized throughout the manuscript.

We appreciate the reviewer's valuable comment. To address this point, we have expanded the Materials and Methods section to include the experimental details for the ELISA and pull-down assays. The relevant figures have been moved to the supplementary information and are now presented as S1D-S1F Fig. Furthermore, the descriptions of ELISA methods, including EC50 and competitive assays, have been harmonized throughout the manuscript.

>> However, as Expi293 cells are not widely recognized for cell surface HER2 protein expression and are not commonly used in breast cancer research, shall we remove the corresponding experimental data and figures regarding HER2 antibody epitope determination in Expi293 cells from the revised manuscript?

This dataset provides evidence of an antibody selection process targeting epitopes distinct from those of T&P, and therefore should not be removed. Although the suitability of using Expi293 cells may be debatable, the experimental conditions are clearly described in the revised manuscript, so no further changes are necessary.

OK, thank you very much.
